# Island life in the Cretaceous - faunal composition, biogeography, evolution, and extinction of land-living vertebrates on the Late Cretaceous European archipelago

**DOI:** 10.3897/zookeys.469.8439

**Published:** 2015-01-08

**Authors:** Zoltán Csiki-Sava, Eric Buffetaut, Attila Ősi, Xabier Pereda-Suberbiola, Stephen L. Brusatte

**Affiliations:** 1 Department of Geology, Faculty of Geology and Geophysics, University of Bucharest, 1 N. Bălcescu Blvd, 010041 Bucharest, Romania; 2 Centre National de la Recherche Scientifique, UMR 8538, Laboratoire de Géologie de l’Ecole Normale Supérieure, 24 rue Lhomond, 75231 Paris Cedex 05, France; 3 MTA-ELTE Lendület Dinosaur Research Group, Pázmány Péter sétány 1/c, Budapest, 1117, Hungary; 4 Universidad del País Vasco/Euskal Herriko Unibertsitatea, Facultad de Ciencia y Tecnología, Departamento de Estratigrafía y Paleontología, Apartado 644, 48080 Bilbao, Spain; 5 School of GeoSciences, University of Edinburgh, Grant Institute, King’s Buildings, West Mains Road, Edinburgh EH9 3JW, UK

**Keywords:** Late Cretaceous, Europe, island, faunal evolution, paleobiogeography, extinction

## Abstract

The Late Cretaceous was a time of tremendous global change, as the final stages of the Age of Dinosaurs were shaped by climate and sea level fluctuations and witness to marked paleogeographic and faunal changes, before the end-Cretaceous bolide impact. The terrestrial fossil record of Late Cretaceous Europe is becoming increasingly better understood, based largely on intensive fieldwork over the past two decades, promising new insights into latest Cretaceous faunal evolution. We review the terrestrial Late Cretaceous record from Europe and discuss its importance for understanding the paleogeography, ecology, evolution, and extinction of land-dwelling vertebrates. We review the major Late Cretaceous faunas from Austria, Hungary, France, Spain, Portugal, and Romania, as well as more fragmentary records from elsewhere in Europe. We discuss the paleogeographic background and history of assembly of these faunas, and argue that they are comprised of an endemic ‘core’ supplemented with various immigration waves. These faunas lived on an island archipelago, and we describe how this insular setting led to ecological peculiarities such as low diversity, a preponderance of primitive taxa, and marked changes in morphology (particularly body size dwarfing). We conclude by discussing the importance of the European record in understanding the end-Cretaceous extinction and show that there is no clear evidence that dinosaurs or other groups were undergoing long-term declines in Europe prior to the bolide impact.

## Introduction

The most iconic picture of a Late Cretaceous terrestrial ecosystem is probably *Tyrannosaurus* attacking *Triceratops* on the vast, fertile floodplains of North America, as a suite of smaller dinosaurs, mammals, crocodyliforms, turtles, and pterosaurs look on. This vignette has been repeated often in movies and museum exhibits, and for good reason: the terrestrial fossil record of the latest Cretaceous in North America is the richest and most complete of anywhere in the world (Weishampel et al. 2004). For this reason, much of our understanding of how dinosaurs and other organisms were living, interacting, and evolving during the final few million years before the K-Pg extinction comes from careful study of the North American record.

In recent years, however, the fossil record of the latest Cretaceous in Europe has improved tremendously. Large-scale fieldwork programs in France, Hungary, Portugal, Romania, and Spain have revealed a wealth of new taxa, ranging from carnivorous, duck-billed, and long-necked dinosaurs to mammals, crocodyliforms, turtles, pterosaurs, squamates, and numerous kinds of fishes. The phylogenetic relationships and paleobiology of many of these taxa have been studied in detail, leading to a better understanding of their evolution and behavior, and how they interacted with each other to form complex terrestrial ecosystems during the final stages of the Age of Dinosaurs. As we learn more about the European faunas, it is becoming increasingly clear that their evolution, paleogeographic composition, and ecologies were complex, and have an important story to tell in regards to how dinosaurs and other organisms were changing before the end-Cretaceous bolide impact.

**Figure 1.**
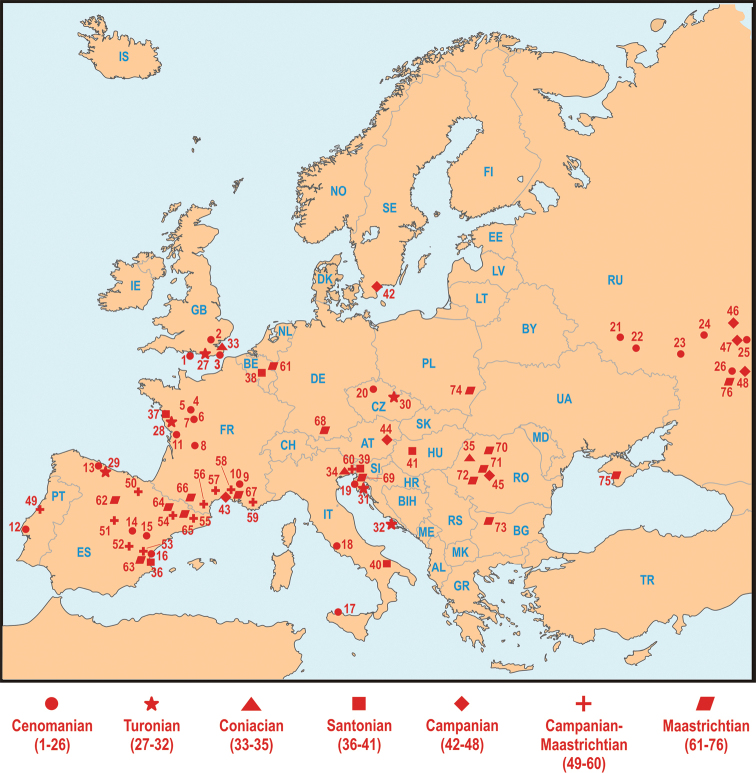
Late Cretaceous vertebrate localities from Europe (including European part of Russia), plotted and listed by age (see text for details and references; localities italicized are detailed in Fig. 4 and in the text, as indicated). Note that one data-point on the map can represent several fossil localities and sites, especially for the Campanian and Maastrichtian. **Cenomanian:** **1** Isle of Wight, southeastern UK **2** Cambridgeshire, southeastern UK **3** Kent, southeastern UK **4** Sarthe, western France **5** Maine-et-Loire, western France **6** Vienne, western France **7** Indre-et-Loire, western France **8** Dordogne, western France **9** Vaucluse, southern France **10** Gard, southern France **11** Charente-Maritime, Charente, western France **12** Carenque, southwestern Portugal **13** Asturias, northern Spain **14** Guadalajara, central Spain **15** Teruel, central-eastern Spain **16** Valencia, eastern Spain **17** Sicily, Italy **18** Lazio, central Italy **19** Istria, Croatia **20** Czech Republic **21** Kursk Oblast, Russia **22** Belgorod Oblast, Russia **23** Voronezh Oblast, Russia **24** Tambov Oblast, Russia **25** Saratov Oblast, Russia **26** Volgograd Oblast, Russia. **Turonian:** **27** East Sussex, southeastern UK **28** Vendée, western France **29** Asturias, northern Spain **30** Czech Republic **31** Istria, Croatia **32** Dalmatia, Croatia. **Coniacian:** **33** Kent, southeastern UK **34** Gorizia, northeastern Italy **35** *Bihor, western Romania* (see section **F**, Fig. 4). **Santonian:** **36** Valencia, eastern Spain **37** Vendée, western France **38** Lonzée, central Belgium **39** Kras, Slovenia **40** Bari, southern Italy **41** *Iharkút, western Hungary* (see section **B**, Fig. 4). **Campanian:** **42** Scania, southern Sweden **43** *Villeveyrac, Languedoc, southern France* (see section **D**, Fig. 4) **44** *Muthmannsdorf, eastern Austria* (see section **C**, Fig. 4) **45** *northwestern Transylvania, Romania* (see section **F**, Fig. 4) **46** Penza Oblast, Russia **47** Saratov Oblast, Russia **48** Volgograd Oblast, Russia. **Late Campanian–Maastrichtian:** **49** *Aveiro-Coimbra districts, Portugal* **50** *Condado de Treviño, northern Spain* **51** *Segovia, central Spain* **52** *Cuenca, central Spain* **53** *Valencia, eastern Spain* **54** *Huesca, northeastern Spain* **55** *Lleida, northeastern Spain* (for **49–55** see section **E**, Fig. 4) **56** *Ariège and Aude, southern France* **57** *Hérault and Gard, southern France* **58** *Bouches-du-Rhône, southern France* **59** *Var, southeastern France* (for **56–59** see section **D**, Fig. 2) **60** Trieste, northeastern Italy. **Maastrichtian:** **61** Limburg, northeastern Belgium, southeastern Netherlands **62** *Burgos, northern Spain* **63** *Valencia, eastern Spain* **64** *Huesca, northeastern Spain* **65** *Lleida, northeastern Spain* (for **62–65** see section **E**, Fig. 4) **66** *Haute-Garonne, Aude, southern France* **67** *Bouches-du-Rhône, southern France* (for **66–67** see section **D**, Fig. 4) **68** Bavaria, southern Germany **69** Kras, Slovenia **70** *northwestern Transylvanian Basin, Romania* **71** *southwestern Transylvania, Romania* **72** *Hațeg and Rusca Montană Basins, Romania* (for **70–72** see section **F**, Fig. 4) **73** Vratsa Province, northwestern Bulgaria **74** Roztocze, southeastern Poland **75** Krymskaya Oblast, Ukraine **76** Volgograd Oblast, Russia.

In this paper, we review the current state of the European Late Cretaceous terrestrial fossil record (Fig. 1). We begin with a paleogeographic overview of Europe during this time, which describes the island archipelago layout of Europe during the high sea levels of the terminal Cretaceous. We then outline the major faunas from Hungary, France, Iberia, and Romania, introduced by a brief overview of the lesser-known faunas from elsewhere in Europe. Next, we discuss the paleogeographic history and assembly of the European faunas, showing that they are a mixture of an endemic ‘core’ augmented with various immigrants from northern and southern continents. This is followed by a discussion about what the European faunas tell us about insular, island communities and evolution during the Mesozoic. Finally, we briefly review the relevance of the European faunas for understanding the end-Cretaceous extinction, and argue that although Europe had experienced some ecological reorganization during the waning years of the Cretaceous, there is no strong evidence that dinosaurs and other organisms gradually wasted away to extinction. In fact, there is now evidence that non-avian dinosaurs were present in Europe within 400,000 years of the K-Pg boundary, the finest resolution permitted by the current fossil record.

Institutional abbreviations: EME – Transylvanian Museum Society, Cluj-Napoca, Romania; HUE – ‘Lo Hueco’ collection, Museo de las Ciencias de Castilla-La Mancha, Cuenca, Spain; LPB (FGGUB) – Laboratory of Paleontology, Faculty of Geology and Geophysics, University of Bucharest, Bucharest, Romania; MC – Musée de Cruzy, Cruzy, France; MCDRD – Muzeul Civilizaţiei Dacice şi Romane, Deva, Romania; MCNA – Museo de Ciencias Naturales de Álava/Arabako Natur Zientzien Museoa, Vitoria-Gasteiz, Spain; MDE – Musée des Dinosaures d’Espéraza, Espéraza, France; MFGI – Geological and Geophysical Institute of Hungary, Budapest, Hungary; MGUV – Museo de Geología de la Universidad de Valencia, Burjassot, Spain; MHNAix – Muséum d’Histoire Naturelle d’Aix-en-Provence, Aix-en-Provence, France; MPZ – Museo de Ciencias Naturales (formerly Museo Paleontológico) de la Universidad de Zaragoza, Zaragoza, Spain; MTM – Hungarian Natural History Museum, Budapest, Hungary; NHMUK – Natural History Museum, London, UK; PIUW – Paläontologisches Institut, University of Wien, Wien, Austria; UBB – Paleontological Collection, Faculty of Biology and Geology, Babeş-Bolyai University, Cluj-Napoca, Romania.

## Europe in the Late Cretaceous: Paleogeography and Paleotectonics of an Ancient Island Archipelago

One widely acknowledged key feature of Late Cretaceous Europe is its extremely discontinuous continental paleogeography, a two-fold consequence of early Mesozoic supercontinent break-up. Fragmentation of Pangea started in the Triassic, but sped up starting with the Jurassic–Early Cretaceous (e.g., Golonka and Bocharova 2000; Seton et al. 2012). This process led to increased rates of seafloor spreading, and development – including in the Mediterranean Tethys area – of several second-order extensional areas (‘oceanic throughs’) that split off continental crust slivers from the major continental landmasses. Sea floor spreading peaked during the ‘mid’-Cretaceous, and steered some of the most important Phanerozoic sea-level highstands (e.g., Seton et al. 2009). The resulting high sea levels that characterized the Cretaceous Period, and especially the Late Cretaceous (e.g., Haq et al. 1987; Miller et al. 2003; Seton et al. 2009; Haq 2014), led to widespread inundation of cratonic areas and significant expansion of epicontinental seas, as well as to drowning of the different intra-oceanic carbonate platforms that formed through sea-floor spreading during the first half of the Mesozoic.

Together, spreading areas and transgressions transformed Europe into an extensive island archipelago for the second half of the Cretaceous, with an important north-south spatial division. In the north, the old consolidated cratonic areas of Europe were covered by epicontinental seaways that divided it into an archipelago of uplifted and emergent pre-Alpine massifs. Towards the south, in the main Tethyan area, the action of raising sea-levels was amplified by active tectonic processes, within a complex mosaic of spreading centers that separated partly emergent continental crustal blocks, subduction zones building chains of volcanic islands, and collisional areas uplifting newly consolidated orogenic chains. And, to complete the picture, in the southwest of Europe, the Iberian fig evolved alternatively as isolated crustal block or as part of cratonic Europe during the Late Cretaceous (e.g., Martín-Chivelet et al. 2002).

**Figure 2.**
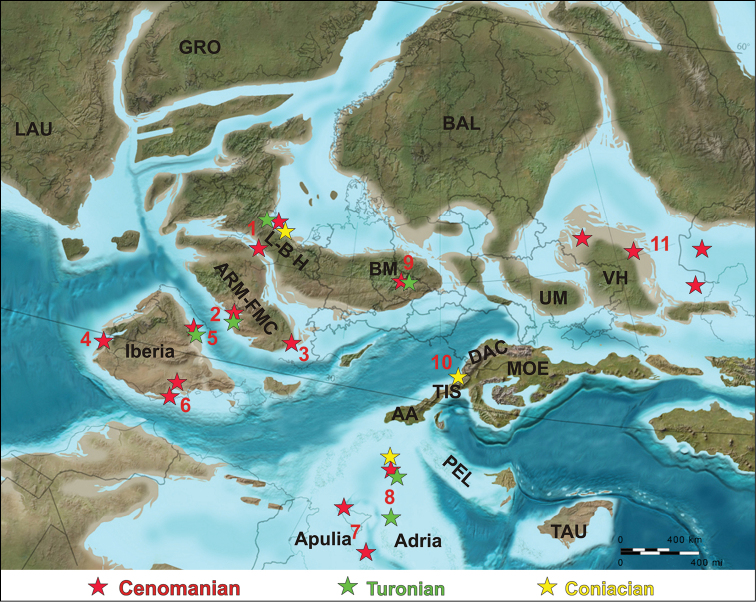
Paleogeographic distribution of the early Late Cretaceous (Cenomanian–Coniacian) European continental vertebrate assemblages (base map for earliest Cenomanian, ~ 100 Mya, courtesy of R. Blakey). Abbreviations: **1** southeastern England **2** western France **3** southern France **4** western Iberia (Portugal) **5** northern Iberia (northern Spain) **6** east-central Iberia (eastern Spain) **7** Apulia (Sicily, central Italy) **8** Adriatic-Dinaric Carbonate Platform (Croatia, Slovenia) **9** Czech Republic **10** northwestern Romania **11** southern Russia (for details, see also Fig. 1 and text); **AA** Austroalpine Domain; **ARM-FMC** Armorica-French Central Massif; **BAL** Baltic Landmass; **BM** Bohemian Massif; **DAC** Dacia Block; **GRO** Greenland; **LAU** Laurentian Shield; **L-B H** London-Brabant High; **MOE** Moesian Platform; **PEL** Pelagonian Domain; **TAU** Taurus Block; **TIS** Tisia Block; **UM** Ukrainian Massif; **VH** Voronezh High.

**Figure 3.**
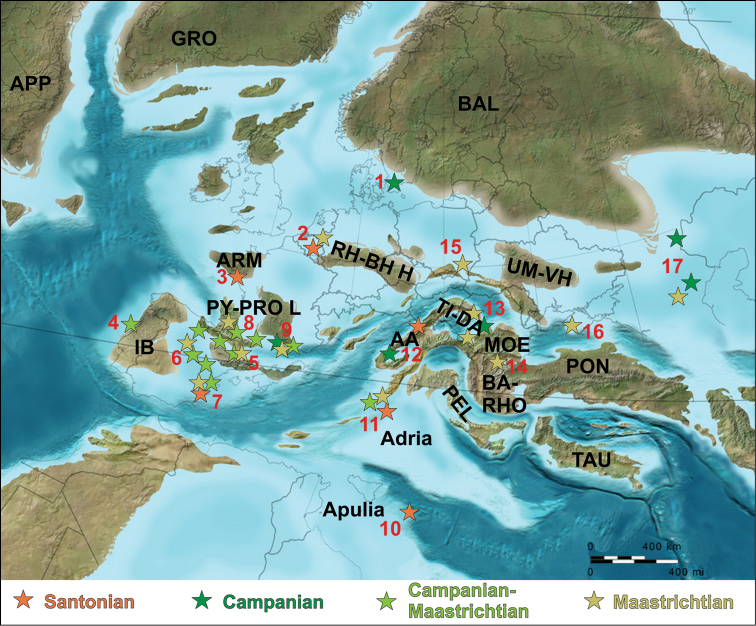
Paleogeographic distribution of the late Late Cretaceous (Santonian–Maastrichtian) European continental vertebrate assemblages (base map for late Campanian, ~ 75 Mya, courtesy of R. Blakey). Abbreviations: **1** Scania (southern Sweden) **2** Belgium-The Netherlands **3** western France **4** western Iberia (Portugal) **5** Cantabrian-southern Pyrenean region (northern Spain) **6** central Iberian region (central Spain) **7** eastern Iberian region (eastern Spain) **8** Languedoc (western southern France) **9** Provence (eastern southern France) **10** Apulia (southern Italy) **11** Adriatic-Dinaric Carbonate Platform (eastern Italy, Slovenia) **12** Austroalpine region (eastern Austria, western Hungary) **13** Transylvania (northwestern Romania) **14** northern Bulgaria **15** southeastern Poland **16** Crimea **17** southern Russia (for details, see also Figs 1, 4 and text); **AA** Austroalpine Domain; **APP** Appalachia; **ARM** Armorican Massif; **BA-RHO** Balkans-Rhodope Orogen; **BAL** Baltic Landmass; **GRO** Greenland; **IB** Iberian Landmass; **MOE** Moesian Platform; **PEL** Pelagonian Domain; **PON** Pontides Orogen; **PY-PRO L** Pyrenean-Provencal Landmass; **RH-BH H** Rhenish-Bohemian High; **TAU** Taurus Block; **TI-DA** Tisia-Dacia Block; **UM**-**VH** Ukrainian Massif-Voronezh High. Note that emergent land was more extensive during Maastrichtian times than represented in the map, with most Spanish-French localities situated in purely continental setting (compare with Fig. 11).

The complexity of paleogeographic and tectonic control factors on the Late Cretaceous evolution of Europe created a very dynamic archipelago-type paleogeography, unlike any other major continental bioprovince of the epoch. Details of the configuration and evolution of this European island archipelago are rather well-known, both from tectonic (Dercourt et al. 1986, 1993, 2000; Ziegler 1988; Csontos and Vörös 2004; Golonka 2004; Golonka et al. 2006; Márton et al. 2008; Schmid et al. 2008; Handy et al. 2010) and paleogeographic-paleoenvironmental (Tyson and Funnell 1987; Vakhrameev 1987; Camoin et al. 1993; Philip et al. 1993; Smith et al. 1994; Dercourt et al. 2000; Bosellini et al. 2002; Martín-Chivelet et al. 2002; Baraboshkin et al. 2003; Zarcone et al. 2010) points of view, and only a few salient features will be synthesized here (Figs 2, 3).

In the northern, cratonic Europe, the main continental areas were represented by the Fennosarmatian landmass, corresponding to emergent areas of the Baltic Shield and Eastern European Platform, the oldest consolidated areas of Europe. Towards the southeast, parts of this old craton were also temporarily emergent as the Ukrainian Massif and the Voronezh High, separated from Fennoscandia by the Polish-Russian Basin. The Russian Basin, connecting for the largest part of the Late Cretaceous the Arctic, Tethyan and West Siberian marine areas, separated the European archipelago from the emergent parts of the Ural Mountains and (farther to the east, across the Turgai Strait) those of Middle Asia. In western and central Europe, the main emergent areas (‘islands’) corresponded to several Caledonian- and Hercynian-aged massifs: the Bohemian Massif in the Czech Republic; the Rhenish Massif in central Germany; the London-Brabant Massif connecting areas of Belgium to the eastern parts of South England across the English Channel; the Irish, Scottish and Cornubian massifs in the British Isles; the Armorican Massif in western France; and the French Central Massif in south-central France. Also parts of the cratonic European archipelago, although separated from the main body of the continent for brief extensional episodes, were the Iberian Meseta in western Spain and Portugal, and the Ebro Massif in eastern Spain.

These major emergent areas expanded and shrunk continuously during the Late Cretaceous, the changes being controlled mainly by eustatic sea-level changes. Although the massifs remained at least partly emerged throughout the entire Late Cretaceous, their dimensions and contours varied; occasionally different landmasses coalesced during periods of significant sea-level drop such as that recognized during the late Maastrichtian, forming more extensive emergent lands. Towards the Santonian–Campanian, the previously detached Iberian fig approached the southern margin of cratonic Europe, and continental convergence started in the Pyrenean trough (Sibuet et al. 2004), followed by local emergence and installation of the oldest continental deposits in the south-Pyrenean domain by the late Campanian (Villalba-Breva and Martín-Closas 2013). The initiation of the Pyrenean mountain building allowed merging of the previously isolated continental areas of the Iberian Meseta, Ebro Massif and French Central Massif, and led to the creation of the most important latest Cretaceous southern European ‘island’, the Ibero-Armorican landmass.

South of the stable, Hercynian-consolidated Europe, the early Mesozoic Tethyan (s.l.) extensional events detached a series of continental crust-floored blocks from the marginal areas of both the European and the African cratons. These blocks were dragged subsequently into the active spreading area of the Tethys Ocean, and each one of them underwent a partly independent tectono-sedimentary evolution, controlled by the combined effects of eustatic sea-level changes and tectonic movements. A large number of such semi-independent blocks were identified and named within the area of the “Mediterranean Seuil” including the Apulian, Austro-Alpine (ALCAPA), Adriatic-Dinaric, Tisia, Dacia, Pelagonian and Rhodope ‘microfigs’; their number, dimension, shape and relative position changed along the Late Cretaceous, but, as a rule of thumb, these emergent areas (‘islands’) were less stable in space and time than those known from cratonic Europe. Starting with the ‘mid’-Cretaceous, the Africa-Europe convergence imprinted a general compressional kinematics to the Mediterranean Seuil area, and previously isolated blocks started to merge and become uplifted through local collisional events to ultimately form the different segments of the Alpine orogenic chains (Alps, Carpathians, Dinarides, Balkans, Appenines) stretching across southern Europe.

The timespan and spatial extent of these Tethyan islands is hard to estimate due to their transient nature and lack of continuous statigraphic sequences. In the Bakony Mountains (Iharkút; part of the Austro-Alpine block), pre-Santonian sediments are represented by thick bauxites, for which a deposition time of a few million years was calculated (Birkeland 1984; Retallack 1990; Mindszenty et al. 1996). These bauxites are overlain by the vertebrate-yielding Santonian Csehbánya Formation. Underneath the bauxites, the latest, well-dated marine sediments are Cenomanian marls (Szives et al. 2007) whose deposition ended no later than the late Cenomanian–earliest Turonian, after which the uppermost beds were removed by erosion (János Haas 2013, pers, comm. to A.Ő.). This means that the „Iharkút landmass” appears to have been existed continuously from the late Turonian up to the middle Santonian, as the presence of the Santonian/Campanian boundary has been pointed out within the Polány Marl Formation that covers the Jákó Marl and the Csehbánya formations (Bodrogi and Fogarasi 1995), thus for a period of approximately 4–6 million years. After the Santonian–early Campanian, most of the Austro-Alpine landmass (Eastern Alps and adjacent areas) was already subsiding and soon became covered by shallow- to deep-water marine basins (Willingshofer et al. 1999; Faupl and Wagreich 2000; Schuller 2004), thus ending the subaerial exposure period of this ‘island’. In Romania, the history of the Transylvanian landmass was even more complex, with smaller or larger emergent areas exposed here starting the Early–Late Cretaceous boundary, and with a significant expansion of the continental areas beginning with the latest Campanian (Benton et al. 2010; Vremir et al. 2014).

To conclude, the unusual paleogeographic setting of Europe during the Late Cretaceous – a fluctuating, tectonically extremely active island archipelago that incorporated both cratonic and intra-oceanic continental islands – makes this province unique during this epoch, corresponding to the last phases of non-avian dinosaurian (and continental vertebrate) existence right before the Cretaceous–Paleogene boundary. As such, it can offer a useful insight into patterns and trends of continental vertebrate evolution during the Late Cretaceous within a setting controlled by entirely different ecological and evolutionary factors than those in action in larger, more contiguous landmasses such as North America.

## The Late Cretaceous Continental Vertebrate Faunas of Europe

### A. General Overview

Europe boasts one of the most complete stratigraphic records of the continental Mesozoic anywhere in the world. Vertebrate fossils are preserved in many portions of this sequence, although this fossil record is highly discontinuous and therefore overshadowed by the more complete, and diverse (but not neccesarily continuous) Mesozoic continental vertebrate assemblages of North America and Asia (e.g., Buffetaut 1997; Evans 2003; Kielan-Jaworowska et al. 2004; Weishampel et al. 2004). The uneven nature of the European Mesozoic continental fossil record is due to the spatial and temporal patchiness of the potentially fossiliferous units, the relatively poor quality of many available outcrops (which are often covered by cities, agricultural land, or vegetation), and the often poor preservational state of the fossils.

The Cretaceous continental vertebrate record of Europe is particularly patchy. Most problematic, there is a remarkable gap corresponding to the first half of the Late Cretaceous epoch that was, until recently, almost completely devoid of significant fossil occurrences (e.g., Buffetaut et al. 1981; Buffetaut and Le Loeuff 1991a; Buffetaut 1997; Hirayama et al. 2000; Kielan-Jaworowska et al. 2004; Weishampel et al. 2004; Barrett et al. 2008; Gardner and Böhme 2008; Martin and Delfino 2010; Ezcurra and Agnolín 2011, 2012; Mannion and Upchurch 2011; Benson et al. 2013; Rage 2013; Roček 2013; Fig. 2). Although isolated occurrences of continental vertebrate fossils were occasionally reported from the Cenomanian to lower Santonian of Europe, these were mainly from marginal marine deposits (e.g., Buffetaut and Pouit 1994). Only very recently have more diverse and well-preserved early Late Cretaceous faunal assemblages been discovered, especially in the Cenomanian of western Europe (see below). This marked early Late Cretaceous faunal gap is a consequence of the substantial reduction in emergent land areas that began in the late Albian–Cenomanian with a major sea-level rise that transformed Europe into an island archipelago (e.g., Smith et al. 2001; Benson et al. 2013). The wildly fluctuating paleogeography of this archipelago was subsequently controlled by sea-level changes and tectonic events affecting the Mediterranean Tethys region during the early formation of the Alpine orogenic chains (Tyson and Funnell 1987; Dercourt et al. 2000; Bosellini 2002; Csontos and Vörös 2004; Golonka 2004; Pereda-Suberbiola 2009; Benton et al. 2010; Weishampel et al. 2010; Zarcone et al. 2010).

Due to this extremely poor early Late Cretaceous continental fossil record from Europe, most previous faunal and paleobiogeographic analyses of Late Cretaceous vertebrates focused on the much better known late Late Cretaceous, especially the Campanian–Maastrichtian taxa (e.g., Buffetaut and Le Loeuff 1991a; Le Loeuff 1991; Le Loeuff and Buffetaut 1995; Pereda-Suberbiola 2009; Fig. 3). During this time, progressive sea-level retreat and collisional events led to the emergence of widespread land areas, which resulted in a much more extensive continental rock record and a much improved vertebrate fossil record (Benson et al. 2013; Table 1).

**Table 1.** Synthetic distribution list of the major continental vertebrate groups in the most important latest Cretaceous European assemblages.

Taxon		Western Hungary	Eastern Austria	Iberian Peninsula	Southern France	Romania
**Fishes**	Pycnodontiformes	X		X		
	Lepisosteiformes	X		X	X	X
	Acipenseriformes					X
	Characiformes				X	X
	Mawsoniidae				X	
	Phyllodontidae			X		
	Palaeolabridae			X		
	Amiidae			X		
	Albulidae			X		
	Osteoglossidae			X		
	Sparidae				X	
**Amphibians**	Albanerpetontidae	X		X	X	X
	Neobatrachia indet. *Hungarobatrachus*	X				
	Discoglossidae	X		X	X	X
	Palaeobatrachidae	X		X	X	
	Pelobatidae	X		X		
	Batrachosauroididae				X	
	Salamandridae			X	X	
**Squamates**	Paramacellodidae			?X		X
	Polyglyphanodontinae	X				X
	Chamopsiidae	X				
	Iguanidae			X	X	
	?Amphisbaenia/Anguidae			X	X	
	Varanoidea			X	X	
	Mosasauroidea	X			X	
	Madtsoiidae			X	X	X
	Alethinophidia			X		
**Turtles**	‘Kallokibotioninae’	X	X			X
	Solemydidae			X	X	
	Bothremydidae	X		X	X	
	Dortokidae	X	X	X	X	X
**Choristoderes**	Champsosauridae		X			
**Crocodyliformes**	Sebecosuchia	X	X	X		X
	Hylaeochampsidae	X		X	X	X
	‘*Allodaposuchus*’	X		X	X	X
	‘*Theriosuchus*’ (Atoposauridae)	X		?X	X	X
	Alligatoroidea			X	X	
	Gavialoidea				X	
	Crocodyloidea			X		
	Eusuchia indet.		X		X	
**Pterosaurs**	Azhdarchidae	X	X	X	X	X
**Saurischian dinosaurs (incl. birds)**	Titanosauria			X	X	X
	basal Tetanurae	X	X			X
	Dromaeosauridae	X		X	X	X
	Abelisauroidea	X		X	X	
	Alvarezsauridae					?X
	Troodontidae			?X		X
	Ornithomimosauria			?X		
	Coelurosauria indet.	X		X	X	X
	Enantiornithes	X		?X	X	X
	Ornithuromorpha			X		X
**Ornithischian dinosaurs**	Nodosauridae	X	X	X	X	X
	Rhabdodontidae	X	X	X	X	X
	Hadrosauroidea			X	X	X
	Ceratopsia	X				
**Mammals**	Multituberculata					X
	Zhelestidae			X	X	
	?Palaeoryctidae			?X	?X	

**Figure 4.**
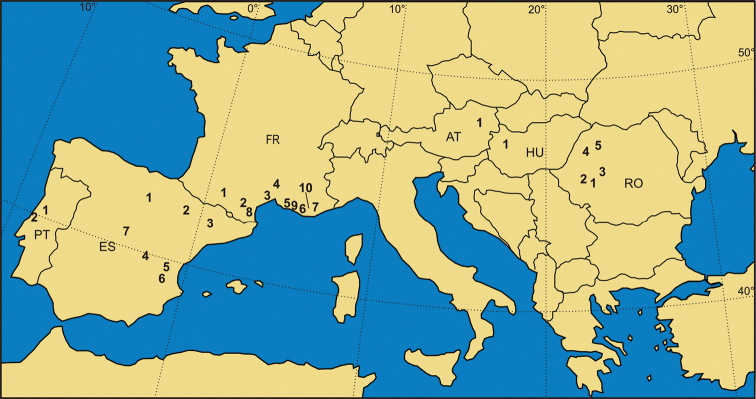
The most important latest Cretaceous (Santonian–Maastrichtian) continental vertebrate localities in Europe. **France:** **1** Lestaillats, Haute-Garonne (Laurent et al. 2002a) **2** Campagne-sur-Aude, Aude Valley (Buffetaut et al. 1989; Le Loeuff 2005) **3** Villeveyrac, Cruzy (Buffetaut et al. 1996, 1999; Buffetaut and Le Loeuff 1991b) **4** Serviers, Champ-Garimond (Buffetaut et al. 1997a; Sigé et al. 1997) **5** Roques-Hautes (Le Loeuff et al. 1992; Allain and Taquet 2000) **6** Beausset Syncline (Le Loeuff and Buffetaut 1991) **7** Fox Amphoux (Le Loeuff et al. 1992) **8** Fontjoncouse (Le Loeuff et al. 1994) **9** Vitrolles-la-Plaine (Valentin et al. 2012) **10** Jas Neuf Sud (Tortosa et al. 2014). **Spain:** **1** Laño (Astibia et al. 1999; Pereda-Suberbiola et al. 2000) **2** Arén (Torices et al. 2004; Weishampel et al. 2004) **3** Tremp (López-Martínez et al. 2001) **4** Lo Hueco, Fuentes (Barroso-Barcenilla et al. 2009; Pérez-García et al. 2012b) **5** Chera (Company et al. 2009b) **6** La Solana (Company et al. 1998, 1999, 2013) **7** Armuña (Buscalioni and Martínez-Salanova 1990; Corral Hernández et al. 2007). **Portugal:** **1–2** Viso, Aveiro, Taveiro (Antunes and Sigogneau-Russell 1991; Antunes and Mateus 2003). **Austria:** **1** Muthmannsdorf (Seeley 1881). **Hungary:** **1** Iharkút and Ajka (Ősi et al. 2012b). **Romania:** **1** Rusca Montană Basin (Vasile and Csiki 2011; Codrea et al. 2012) **2** Haţeg Basin (Grigorescu 2005, 2010; Weishampel et al. 2010) **3** Alba Iulia-Sebeș (southwestern Transylvanian Basin; Codrea et al. 2010a,c; Vremir 2010) **4** Borod Basin (Nopcsa 1902b) **5** Jibou (northwestern Transylvanian Basin; Codrea and Godefroit 2008; Codrea et al. 2010c).

Although recent discoveries have helped to fill some of the early Late Cretaceous fossil gap, there is still a clear dichotomy between the quality of continental fossil record from the early Late and late Late Cretaceous of Europe. Fossiliferous units from the last ~20 million years of the Cretaceous are still yielding the most diverse and well-preserved European continental vertebrate faunas (Fig. 4). Chief among these are deposits from the Santonian of Hungary (see below, section **B**), the lower Campanian of Austria (section **C**), the Campanian–Maastrichtian of southern France (section **D**) and of the Iberian Peninsula (section **E**), as well as the uppermost Campanian–Maastrichtian of Romania (section **F**). Accordingly, these major European faunal assemblages will be described in detail separately in this paper. Before presenting these descriptions, however, we first briefly review the remaining European Late Cretaceous continental vertebrate fossil record in chronostratigraphically ascending order.

#### Cenomanian

The best documented pre-Santonian Late Cretaceous continental vertebrate assemblages in Europe derive from Cenomanian deposits. These occurrences stretch from England in the west to Croatia and European Russia in the east, respectively from the Czech Republic to the north to Iberia and Sicily in the south (Figs 1, 2). Despite seemingly large areal coverage, most of the occurrences are preserved in littoral or shallow marine deposits, and are merely isolated and often very fragmentary skeletal remains (e.g., Buffetaut et al. 1981; Weishampel et al. 2004), with the notable exception of the newly described faunal assemblages from the Charentes region of western France (e.g., Néraudeau et al. 2003; Vullo and Néraudeau 2008) and the Asturias in northern Spain (Vullo et al. 2009). Moreover, a great number of the known Cenomanian fossils are tetrapod (mostly dinosaur) footprints, which are also preserved in tidal and littoral deposits (e.g., Dalla Vecchia 2001, 2008; Nicosia et al. 2007). The dominance of trace and body fossils in shallow marine deposits is a consequence of the expansion of shallow marine habitats during the Cenomanian sea-level highstand, which drowned previously emergent land areas (Miller et al. 2003; Galeotti et al. 2009).

Two particular biases affect studies of the Cenomanian European continental vertebrate record. First, as mentioned above, there is a taphonomic bias, in that most specimens (trace and body fossils) are found in nearshore marine deposits, and very few specimens are found in strictly terrestrial rocks. Second, both the trace and body fossil record is almost completely dominated by one group, dinosaurs, which is surely an artificial skew. As a result of these biases, very little is known about the detailed composition, ecology, possible spatial and temporal heterogeneity, or biogeographic affinities of the European Cenomanian continental vertebrate faunas. This will hopefully change in the future with continued exploration of potentially fossiliferous areas, as shown by the recent discoveries of more diverse and well-preserved Cenomanian continental faunas in Charentes (France) and Asturias (Spain).

The first known Cenomanian continental vertebrate fossils from Europe were described from southeastern England, and western and southern France (Buffetaut et al. 1991; Weishampel et al. 2004; Rodrigues and Kellner 2013). These assemblages are dominated by archosaurs, in particular crocodyliforms, pterosaurs, and dinosaurs. In southeastern England, the shallow-marine deposits of the Grey Chalk Subgroup (= Chalk Marl; Hopson 2005) have yielded isolated remains of pterosaurs, including the lonchodraconid *Lonchodraco* and the pteranodontoid *Cimoliopterus*, along with many taxa considered *nomen dubia* (Rodrigues and Kellner 2013). These are associated with dinosaur fossils, including the indeterminate nodosaurid ‘*Acanthopholis horridus*’ (Pereda-Suberbiola and Barrett 1999) and the non-hadrosaurid hadrosauroid ‘*Iguanodon hilli*’ (Dalla Vecchia 2009b). The basalmost, glauconite- and phosphate-bearing siliciclastic lower Cenomanian deposits of the Grey Chalk Subgroup (Glauconitic Marls Member; Hopson 2005) are more richly fossiliferous, and have produced many fossils of pterosaurs (e.g., Unwin 2001; Rodrigues and Kellner 2013) and dinosaurs (including sauropods, nodosaurids, and derived euornithopods; Le Loeuff 1993; Pereda-Suberbiola and Barrett 1999; Blows 2014). These specimens are usually regarded as reworked from the underlying Albian beds and will not be discussed further here.

Across the English Channel, shallow-water marine deposits that represent similar lithofacies as those from England outcrop in the Sarthe, Indre-et-Loire, Maine-et-Loire, and Vienne departments of southwestern France. Until recently they have yielded only fragmentary remains of turtles, crocodyliforms, and various dinosaurs (Buffetaut et al. 1981, 1991; Buffetaut 1989c; Buffetaut and Pouit 1994; Buffetaut and Brignon 1999). Although most vertebrate remains are taxonomically indeterminate, the presence of titanosauriform sauropods, nodosaurids, and iguanodontoids suggests that this assemblage is largely similar in composition to that known from southeastern England. Even scrappier material has been reported from the lignite-bearing Cenomanian deposits of southern France, most notably a single isolated theropod tooth from Gard (assigned to ‘*Megalosaurus*’) and a sauropod humerus from Vaucluse, both of which are now lost and whose exact affinities are uncertain (Buffetaut et al. 1991).

Recently, however, a diverse and well-preserved continental vertebrate assemblage was discovered from lower Cenomanian paralic and littoral deposits of the Charentes region in western France, first mentioned by Néraudeau et al. (2003). Although most of these fossils are found in microvertebrate bonebeds (e.g., Vullo et al. 2010), macrovertebrate-dominated sites are also known. Fieldwork over the last decade has revealed a surprisingly diverse continental vertebrate assemblage (associated with a rich marine assemblage) at the beginning of the Late Cretaceous in Europe. Additionally, these sites document one of the major paleobiogeographic changes in Cretaceous Europe: the transition from continental-dominated to purely marine assemblages (Vullo and Néraudeau 2008), corresponding to the advancement of the Cenomanian transgression over the margins of the French Central Massif.

A short review of the Charentes assemblage is warranted. Freshwater fishes, represented by lepisosteiforms (Vullo and Néraudeau 2008), are very rare at Charentes and are far outnumbered by marine taxa. Marine tetrapods are, on the other hand, far less common than continental species. Anuran remains are exceedingly rare (Vullo et al. 2011). Turtles are represented by indeterminate solemydids and dortokids, along with possible charettochelydids (Vullo et al. 2010). Squamate assemblages are diverse, but are dominated by aquatic (most probably marine) taxa, with a lesser presence of terrestrial or potentially terrestrial forms of mainly indeterminate affinity (including a scincomorph; Vullo et al. 2011). Crocodyliforms are represented mainly by families known from older, Lower Cretaceous deposits of Europe, such as the atoposaurids, bernissartiids, goniopholidids, and pholidosaurids (Vullo and Néraudeau 2008). Vullo et al. (2005) also noted the occurrence of a possible ziphosuchian reminiscent of the North African *Hamadasuchus*. Isolated teeth suggest the presence of an indeterminate ornithocheirid pterosaur (Vullo and Néraudeau 2009).

The dinosaurian fauna from Charentes is relatively diverse. The taxic composition of this fauna mirrors the largely fragmentary remains from elsewhere in the Cenomanian of Europe, in that it includes titanosauriform sauropods (including possible brachiosaurids), nodosaurids, iguanodontians, and theropods (Vullo et al. 2007; Vullo and Néraudeau 2010). Iguanodontian postcranial remains were first reported as belonging to an iguanodontid (Néraudeau et al. 2003), but subsequent discovery of isolated teeth instead suggested that this taxon is an indeterminate (probably basal) hadrosauroid (Vullo et al. 2007). Theropod dinosaurs are particularly diverse, with indeterminate dromaeosaurids, troodontids and carcharodontosaurids identified based on isolated teeth. Along with these teeth, the theropod record also includes an isolated body contour feather, but it is unclear whether this belongs to a feathered non-avian theropod or an early bird (Vullo et al. 2013). No other potential bird fossils have been found at Charentes to date.

The last major component of the Charentes assemblage is mammals. Néraudeau et al. (2005) first reported mammals from this area based on a taxonomically indeterminate premolar. Subsequently, more diagnostic isolated fragmentary molars were described as the marsupialiform (stem-marsupial) *Arcantiodelphys* (Vullo et al. 2009b), considered the oldest unequivocal representative of Theria in Europe.

Similar to the case with Charentes, recent discoveries from Spain have substantially improved the Cenomanian continental vertebrate record from southern Europe. Up until very recently, only a few isolated fossils of continental taxa were known from the Cenomanian of the Iberian Peninsula. These were almost entirely limited to dinosaurian footprints: Cuenca-Bescós et al. (1999) reported the presence of middle-sized theropod footprints in Teruel Province, and Antunes and Mateus (2003) mentioned the occurrence of sauropod and potential theropod footprints in southwestern Portugal, near Lisbon. Ruiz-Omeñaca et al. (2009) listed additional Cenomanian tracksites from Spain, but most of them remain undescribed to date; these include a few isolated occurrences in the lowermost and middle to upper Cenomanian of Asturias, in northern Spain, and the Cenomanian of Valencia, in eastern Spain. The presence of Cenomanian theropods in Iberia was further supported by an isolated tooth, possibly of a carcharodontosaurid, reported from the lower-middle Cenomanian of northern Spain by Ruiz-Omeñaca et al. (2009).

The meager record of Cenomanian vertebrates from Iberia was dramatically improved by the discovery of relatively rich fossil assemblages in near-shore, shallow-marine deposits. In northern Spain (Asturias), the tidally influenced coastal lagoon sediments of the basal La Cabaña Formation have yielded a mixed marine-continental assemblage. This fauna includes indeterminate turtles, possible alligatoroid eusuchians, ornithocheirid pterosaurs, and titanosaurian sauropods (Vullo et al. 2009a). The late Cenomanian Asturias assemblage shares several marine taxa with fully marine assemblages from Charentes that are slightly younger than those that yielded the continental vertebrates. The Asturias deposits are particularly important in that they yield the last definitive body-fossil records of titanosaurs in western Europe prior to the late Campanian. Rocks roughly synchronous with those of Asturias are known from Guadalajara, in central Spain. These deposits of the Utrillas Sandstone Formation accumulated as intertidal-subtidal coastal bars and channels. The vertebrate fossils found in these beds are predominantly continental, and include turtles (including solemydids and indeterminate eupleurodirans), derived neosuchian crocodyliforms, and carcharodontosaurid theropods (Torices et al. 2012).

The Iberian continental vertebrate faunas inhabited the marginal areas of an emergent land (the Iberian Meseta Island) that was slowly submerging towards the end of the Cenomanian, as shown by the purely marine deposits covering the vertebrate-bearing coastal units in both northern and central Spain. The other, isolated Iberian occurrences also formed along the margins of the same emergent area. Meanwhile, the Charentes assemblage from France populated a more northern emergent area (the Central Massif Island), the two landmasses being separated by the actively opening Biscay Gulf.

Compared to Iberia and France, continental vertebrate fossils from the Cenomanian of central Europe are much rarer and less studied, and they are represented mainly by trace fossils. The rarity of fossils here reflects the highly fragmented paleogeography of this region, which was mostly underwater in the Cenomanian and located in the northern Tethyan realm (e.g., Dercourt et al. 2000; Bosellini 2002). In Sicily, a dinosaur bone fragment (considered to represent an indeterminate theropod) was discovered in a upper Cenomanian limestone succession formed in marginal, peritidal-to-lagoonal areas of the Africa-related Panormide Carbonate Platform (Garilli et al. 2009). In central Italy, three superposed track-bearing levels with numerous small-sized sauropod and medium-sized theropod footprints were reported from Cenomanian tidal flat to inner lagoon limestone deposits showing dessication cracks and other signs of frequent and recurrent emersion events (Nicosia et al. 2007). These deposits formed in near-coastal settings of another intra-Tethyan carbonate platform, the Lazio-Abruzzi-Campania (or Apenninic) Carbonate Platform, part of the Africa-derived Apulia microfig (Vlahović et al. 2005). More to the north, several dinosaur tracksites of Cenomanian-age have been reported from shallow-water, subtidal-lagoonal-to-intertidal limestone deposits of the Istria Peninsula of Croatia. The tracks were made by small-to-medium-sized theropods and small-sized sauropods (Dalla Vecchia 1998, 2008; Mezga and Bajraktarevič 1999; Dalla Vecchia et al. 2001; Mezga et al. 2006b). These dinosaurs inhabited short- or long-term emergent areas of the Adriatic-Dinaric Carbonate Platform, the northernmost (in present-day geographic direction) fragment of the Apulian microfig (Vlahović et al. 2005).

North of the Alpine Tethyan areas, only a single isolated continental vertebrate fossil is known from the Cenomanian of Central Europe. This specimen is an isolated, rather well preserved femur of a small iguanodontian ornithopod dinosaur, discovered in the upper Cenomanian rudist-bearing sandy beach deposits of the Peruc−Korycany Formation, in the Czech Republic (Fejfar et al. 2005). This dinosaur would have lived on one of the nearby emergent landmasses: the Rhenish-Bohemian Massif, the Kutná Hora ‘Island’ or the East-Sudetan Block.

The easternmost occurrences of Cenomanian continental vertebrates of Europe have been recorded from the southern part of European Russia, from across a large area stretching from the border with Ukraine in the west to northwest of the Caspian Sea in the east. Here, the phosphorite-bearing sandy coastal sediments of the Cenomanian Sekmenovsk and Melovatka formations, as well as several correlative deposits (in Belgorod, Voronezh, Tambov, Saratov and Volgograd oblasts), have yielded several isolated fragmentary specimens of ornithocheirid, lonchodectid, and possibly istiodactylid pterosaurs, as well as indeterminate pterodactyloids (Averianov 2004, 2007a; Averianov et al. 2005; Averianov and Kurochkin 2010). In addition to pterosaurs, Cenomanian dinosaurs are also known from European Russia. Kiprijanow (1883) described extremely fragmentary remains from the Sekmenovsk Formation in Kursk Oblast as a theropod ‘*Poikilopleuron schmidti*’. Although not diagnostic at the species level, these fossils were provisionally accepted as belonging to theropods by Carrano et al. (2012). A fragmentary bone identified as theropod, as well as possible basal hadrosauroid remains (including a tooth) were reported from the same marginal marine deposits of the Sekmenovsk Formation in Belgorod Oblast by Nessov (1995); the hadrosauroid remains were described subsequently by Arkhangelsky and Averianov (2003). Finally, the middle Cenomanian deposits of the Melovatka Formation in Volgograd Oblast also yielded a well-preserved avian brain mold, first described as belonging to the possible enantiornithine *Cerebavis* (Kurochkin et al. 2006), but later reinterpreted as a basal ornithurine (Kurochkin et al. 2007).

In summary, despite the highly fragmentary nature of the Cenomanian continental vertebrate fossil record of Europe, it paints a general picture of the terrestrial dinosaur-dominated faunas from this time. Some basic conclusions about these faunas can be summarized, although they must be regarded with caution due to the patchy Cenomanian fossil record. First, tectonics- and eustasy-driven paleogeographic fragmentation apparently did not cause regional faunal differences, as demonstrated by the largely similar English, French and Spanish faunas. Even the geographically distant Russian faunas seem to have similarities at broad taxonomic levels with those of southeastern England and western France (Averianov 2007a; Vullo et al. 2007; Averianov and Kurochkin 2010). Second, the same general continental faunas are found in the late Early Cretaceous and the Cenomanian of Europe, demonstrating ecological continuity between these time intervals. This is in contrast to the marked endemic character of European continental faunas developed later in the Late Cretaceous (e.g., Pereda-Suberbiola 2009; Weishampel et al. 2010; Ősi et al. 2012b). With that said, there are some new taxa that appear in the Cenomanian record in Europe that may give insight into broader biogeographic patterns. These include troodontids, basal hadrosauroids, and marsupialiforms, which may suggest Laurasian paleogeographic connections (Vullo et al. 2007, 2009b), respectively, eupleurodires and carcharodontosaurids, which may reflect Gondwanan influences (Vullo et al. 2007; Torices et al. 2012). Finally, a notable feature of the dinosaurian fossil record of the European Cenomanian is an apparent trend towards reduction in body size in some taxa, compared to close relatives from outside of Europe. This is especially apparent in the geographically fragmented southern carbonate platforms of the Tethyan areas (Dalla Vecchia 2003, 2008), and the hadrosauroid from Belgorod Oblast, Russia, is also reported to have been particularly small in size (2 m height, according to Nessov 1995).

#### Turonian

The European continental vertebrate fossil record is virtually non-existent for the Turonian (Figs 1, 2). This is a consequence of rising sea-levels all across Europe during this time, which inundated previously emergent land areas and wiped out habitats frequented by dinosaurs and other land-living vertebrates. This inundation was at a maximum around the Cenomanian–Turonian boundary (Miller et al. 2003; Fig. 2). The transition from terrestrial to marine sedimentation is seen in many local sections that preserve the Cenomanian vertebrate fossils described above. For example, in southern England deposition of the Cenomanian Gray Chalk Subgroup was replaced by that of the more open marine White Chalk Subgroup (Hopson 2005). Similar stratigraphic sequences are seen in Charentes in France (Vullo and Néraudeau 2008), as well as in Asturias (Vullo et al. 2009a) and Guadalajara (Torices et al. 2012) in Spain, and in the Bohemian Cretaceous Basin of the Czech Republik (Fejfar et al. 2005). Drowning of the previously tidal-dominated shallow carbonate platform environments is also documented in the Adriatic-Dinaric Carbonate Platform (Vlahović et al. 2005) and the Apulian Platform (Galeotti et al. 2009) in the latest Cenomanian–early Turonian. Although inner platform environments were re-established later during the Turonian, the Cenomanian–Turonian sea-level rise most likely caused a serious evolutionary bottleneck for the vertebrate assemblages inhabiting the previously larger emergent areas (see also Dalla Vecchia 2003).

The Cenomanian–Turonian sea-level rise led to a serious reduction in emergent land across all of Europe (e.g., Dercourt et al. 2000), and a correlated reduction in continental vertebrate-bearing units (Mannion and Upchurch 2011; Benson et al. 2013). This is reflected in the substantially poorer continental fossil record in the Turonian compared to the preceding Cenomanian, in terms of quality of preservation, taxonomic diversity, and areal coverage (Fig. 1). Nonetheless, a small handful of Turonian continental vertebrates have been found in Europe.

In western Europe, continental vertebrate remains have been reported from the basal White Chalk Subgroup, including isolated, fragmentary remains of ornithocheirid pterosaurs. Meanwhile, in the northeastern Czech Republic, pterosaurs are represented by a possible azhdarchoid (*Cretornis*) reported from the Jizera Formation (= Middle Iser Shales; Averianov 2014). In western France, the only Turonian continental vertebrate fossils are from the upper Turonian shallow-water calcareous limestones of Vendée (Buffetaut et al. 1991; Buffetaut and Pouit 1994). These specimens, represented mainly by isolated, worn teeth, document the presence of indeterminate turtles, tribodont crocodylians, and possibly theropod dinosaurs. In Iberia, a still undescribed tracksite from Asturias, northern Spain, made by unidentified dinosaurs, was listed by Ruiz-Omeñaca et al. (2009).

In the southern, Tethyan areas of Europe, Turonian continental vertebrates are represented by dinosaur footprints discovered in the shallow marine carbonates of the Adriatic-Dinaric Carbonate Platform. Mauko and Florjančič (2003) reported small to medium-sized tridactyl (ornithopod or theropod) footprints from shallow marine rudist-bearing limestones in Istria (Croatia). Later, Mezga et al. (2006a) mentioned the occurrence of tracks and trackways of rather large sauropods (most probably titanosaurs; Mannion and Upchurch 2011) preserved in limestones of the Gornji Humac Formation (Dalmatia, Croatia), which were deposited in intertidal-to-shallow subtidal zones of a protected carbonate platform. Both of these occurrences are dated as late Turonian–early Coniacian.

Due to the scarcity of available data, not much can be said about the composition and spatial heterogeneity of the European continental faunas of this age. It appears that ornithocheirids survived in Europe until the Turonian whereas they were replaced by toothless derived pterodactyloids in Asia and North America (Barrett et al. 2008). The Adriatic footprint record undermines the ‘European sauropod hiatus’ hypothesis (Le Loeuff 1993; Le Loeuff and Buffetaut 1995) according to which sauropods went extinct in Europe during the Cenomanian and were reintroduced, supposedly through immigration from Gondwana during the Campanian (see also Mannion and Upchurch 2011). It also weakens the support for the hypothesis of ‘herbivorous replacement’ put forward by Dalla Vecchia (2003), who suggested that sauropods were replaced by hadrosauroids as the dominant herbivores of the Adriatic-Dinaric Carbonate Platform around the time of (and due to) the late Cenomanian–early Turonian sea level highstand and platform drowning event. The Adriatic dinosaur track record shows that while large-sized sauropods were still present locally around the Turonian–Coniacian boundary, the large-sized and broad-footed tridactyl tracks of hadrosauroids do not appear to have been present yet.

#### Coniacian

The Coniacian was a period of high relative sea-level standstill (Miller et al. 2003; Haq 2014), although local variations in relative sea-level have been documented, especially in the more volatile Tethyan areas (Galeotti et al. 2009). Accordingly, most of Europe remained submerged during this time, meaning that the continental vertebrate fossil record is poor and restricted to isolated fragments recovered from shallow marine deposits (Figs 1, 2).

In western Europe, a possible azhdarchoid pterosaur was described by Martill et al. (2008) from Coniacian deposits of the White Chalk Subgroup, England. It represents the youngest record of the group currently known from the British Isles. Another skeletal element, discovered in Coniacian chalk beds of northern France, was once referred to a pterosaur but was recently shown to belong to a marine vertebrate, most probably a turtle (Buffetaut 2008).

Additional Coniacian continental vertebrate remains have been reported from southern Europe, in Gorizia Province of northeastern Italy, and in Romania (the Romanian occurrence will be covered in section **F**). In Gorizia Province, shallow marine, carbonate platform limestones of the Mt. San Michele Limestone Formation have yielded an assemblage dominated by fishes, associated with numerous well-preserved plant impressions. A few non-marine turtle bones and two isolated crocodyliform teeth were also found (Dalla Vecchia and Tentor 2004). The fossil beds are Coniacian–Santonian in age and were interpreted as having formed in a restricted lagoonal environment with tidal influences. Although most of the continental vertebrate fossils are indeterminate, one ziphodont conical tooth was tentatively referred to *Doratodon* by Dalla Vecchia and Tentor (2004). The specimen was later re-described in detail by Dalla Vecchia and Cau (2011) as an as yet unnamed, possibly new notosuchian taxon of Gondwanan affinities. Finally, it should be mentioned that Weishampel et al. (2004) listed a southern Italian tracksite (Altamura) as Coniacian–Santonian in age; however, this site is now dated as Santonian and will be thus discussed below.

The poor Coniacian (and Turonian) fossil record of European continental vertebrates is especially frustrating because the middle part of the Late Cretaceous apparently corresponded to a time of profound faunal restructuring in Europe. Although the origins of the latest Cretaceous faunas of Europe can be traced back to those of the Early Cretaceous (e.g., Weishampel et al. 2010; Ősi et al. 2012b), they are derived in many respects compared to their predecessors and were also affected by immigrations from other continental landmasses. Most of these changes would have taken place during the first half of the Late Cretaceous, and in particular during the Turonian–Coniacian. The very limited information available on the composition and distribution of the Coniacian faunas (only three datapoints) impedes our understanding of the evolutionary events during this time interval. However, it appears that the transition from ornithocheirid-dominated pterosaur assemblages to azhdarchoid-dominated ones, characteristic for the later part of the Late Cretaceous of western Europe (e.g., Buffetaut 2008), took place around the Turonian–Coniacian, since ornithocheirids are no longer reported from these areas from post-Turonian deposits.

#### Santonian

**Figure 5.**
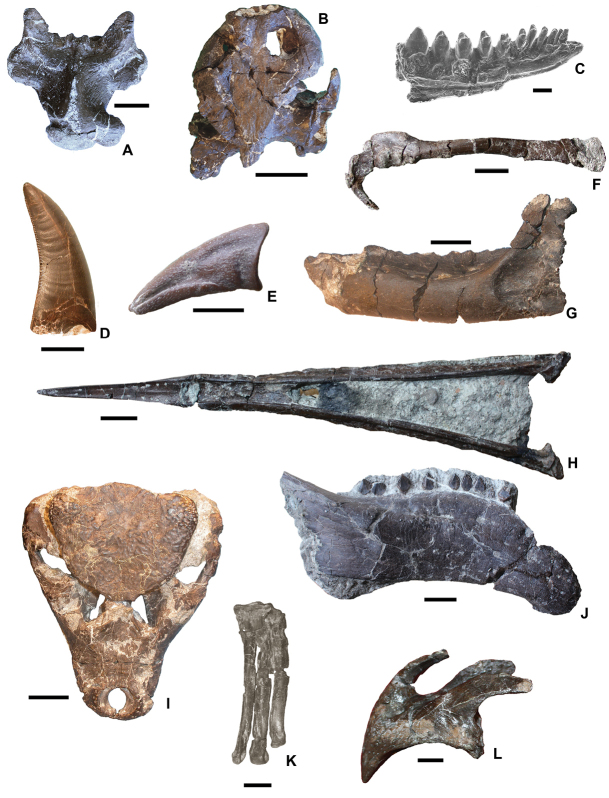
Representative taxa from the Santonian Iharkút fauna from the Csehbánya Formation, Bakony Mountains, western Hungary. **A** *Pannoniasaurus inexpectatus* (Squamata, Mosasauroidea), dorsal vertebra (MTM uncatalogued) in dorsal view (photo by Réka Kalmár) **B** *Foxemys trabanti* (Pleurodira, Bothremydidae), skull (MTM V 2010.215.1.) in dorsal view (photo by Márton Rabi). **C** Bicuspidon aff. hatzegiensis (Squamata, Borioteiioidea), left dentary (MTM 2006.112.1.) in medial view (photo by László Makádi) **D** Basal tetanuran (Theropoda, Tetanurae), tooth (MTM V.01.54) in ?lingual view **E** Indeterminate abelisaurid (Theropoda, Abelisauridae), pedal ungual phalanx (MTM V 2008.43.1.) in lateral view **F** *Pneumatoraptor fodori* (Theropoda, Paraves), left scapulocoracoid (holotype, MTM V 2008.38.1.) in lateral view **G** *Mochlodon vorosi* (Ornithopoda, Rhabdodontidae), left dentary (holotype, MTM V 2010.105.1) in lateral view **H** *Bakonydraco galaczi* (Pterosauria, Azhdarchidae), mandible (holotype, MTM 2007.110.1) in dorsal view **I** *Iharkutosuchus makadii* (Eusuchia, Hylaeochampsidae), skull (holotype, MTM 2006.52.1) in dorsal view **J** *Hungarosaurus tormai* (Ankylosauria, Nodosauridae), right dentary (MTM 2007.25.2) in lateral view **K** *Bauxitornis mindszentyae* (Aves, Enantiornithes), left tarsometatarsus (holotype, MTM V 2009.38.1) in anterior view **L** *Ajkaceratops kozmai* (Ceratopsia), fused rostral and premaxillae (holotype, MTM V 2009.192.1) in lateral view. Scale bars: 2 cm in **A, V, G, H, I, J**; 1 cm in **D, E, F, K, L**; 1 mm in **C.**

The Santonian marks an important turning point in the nature and quality of the European continental vertebrate fossil record (Figs 1, 3). Although sea levels were still relatively high during the Santonian (Miller et al. 2003; Haq 2014), tectonic events related to the completion of the Eoalpine orogeny led to the emergence of large land areas in central and southeastern Europe, extending from the Eastern Alps in Austria to the Southern Carpathians in Romania. These tectonic processes began during the latest Turonian–Coniacian and continued into the Campanian (Willingshofer et al. 1999; Faupl and Wagreich 2000; Schuller 2004). These orogenetic events had far-ranging effects, producing local uplifts even in the northern parts of the Adriatic-Dinaric Carbonate Platform (Otoničar 2007); lithological markers (bauxites) suggest temporary emergence of land in the more southern Apulian Carbonate Platform (Nicosia et al. 2000a). As a result, large areas in central-southern Europe became (although mostly temporarily) subaerially exposed, allowing colonization by continental faunas. Thus, even discounting the important and well-studied Iharkút fossil site from Hungary (Ősi et al. 2012b – see below, section **B**, and Figs 4, 5), the otherwise still poorly sampled Santonian vertebrate record from Europe is nevertheless of special significance.

Santonian specimens were among the first continental vertebrate fossils reported from the Upper Cretaceous of Europe (Dollo 1883). These early discoveries were isolated dinosaurian remains described from shallow-marine glauconite-bearing detritic deposits of the Lonzée Member, in central Belgium. Two taxa were named from Lonzée. The first of these, *Craspedodon*, was for a long time regarded as a derived iguanodontian (e.g., Norman 2004), but was recently reinterpreted as a basal ceratopsian by Godefroit and Lambert (2007). The second, “*Megalosaurus lonzeensis*”, based on an isolated manual ungual, was often suggested to be an indeterminate ornithomimosaur (e.g., Mateus et al. 2010), but is now considered an indeterminate coelurosaur (Carrano et al. 2012).

More recently, continental vertebrate fossils have been reported from farther south in Europe. Without doubt, the most important Santonian vertebrate discovery comes from the western Hungary (Ősi et al. 2003; Ősi 2004); it will be described below (section **B**). Other discoveries include specimens of indeterminate chelonians, crocodyliforms (including taxa with a crushing dentition), and theropods from near-shore to coastal deposits of Vendée, western France (Buffetaut et al. 1991; Buffetaut and Pouit 1994). In Valencia (eastern Spain), theropod and ornithopod footprints are known from Santonian–lower Campanian intertidal to subtidal carbonates (Santisteban and Suñer 2003). Two occurrences are known from more southerly, Tethyan carbonate platform areas. Buffetaut et al. (2002b) reported a fossil feather from shallow-marine rudist-bearing limestones of the Lipica Formation in southwestern Slovenia. This Santonian–Campanian unit was deposited along the northern margin of the Adriatic-Dinaric Carbonate Platform. In southern Italy, an important dinosaur tracksite was discovered in intertidal deposits belonging to the carbonate platform deposits of the Altamura Limestone Formation, accumulated on the Apulian Carbonate Platform (Nicosia et al. 2000a; Dal Sasso 2003). This tracksite yielded thousands of footprints, many of which belong to small-to-medium-sized hadrosauroids with a quadrupedal gait (*Apulosauripus*; Nicosia et al. 2000b). It is worth noting, however, that recently the *Apulosauripus* tracks had been referred to an indeterminate thyreophoran (e.g., Dalla Vecchia 2009b; Petti et al. 2010; Apesteguía and Gallina 2011). Other, less common tracks were identified as belonging to small sauropods and ankylosaurs, whereas theropods appear to be absent or very poorly represented (Nicosia et al. 2000a; Del Sasso 2003).

Some comments on the faunal composition and biogeography of the Santonian continental assemblages can be gleaned from the known record of fragmentary fossils (not yet considering information derived from the Hungarian Iharkút assemblage, which will be discussed in full below). One significant feature is the presence of sauropods on the Tethyan island area of Apulia. Together with the recent discovery of late Turonian–early Coniacian sauropods in the Adriatic-Dinaric Carbonate Platform (see above), recognition of sauropods in the Santonian of Apulia argues against a prolonged “sauropod hiatus” in Europe. Perhaps these isolated Tethyan island areas were refugia for certain taxa (including sauropods) during periods of sea-level high-stands of the mid-Late Cretaceous that inundated extensive land areas in cratonic Europe. This hypothesis is similar to the “inland herbivore” (Lucas and Hunt 1989) or “highland immigrant” (Lehman 2001) scenarios proposed for North America. Butler and Barrett (2008; see also Mannion and Upchurch 2010) demonstrated that Cretaceous sauropods, and especially titanosaurs, preferred inland continental habitats. This may explain the pattern that led Le Loeuff (1993) to propose the “European sauropod hiatus” - the absence of sauropod body fossils from the Cenomanian to the late Campanian of Europe. This absence may be due to the fact that very few suitable inland areas remained for sauropods to inhabit, with the exception of the geographically limited refugia where footprints are commonly preserved but body fossils are exceedingly rare for taphonomic reasons. Regardless of whether these Tethyan platforms were refugia, the occurrence of sauropod footprints demonstrates that the “sauropod hiatus” hypothesis is incorrect.

Some other interesting observations deserve mention. Hadrosauroids, sauropods, and ankylosaurs co-occur in Apulia. Hadrosauroids and sauropods also lived together in the same environments in the Maastrichtian faunas of Romania (Nopcsa 1923a; Weishampel et al. 1991, 2010; see also below, section **E**), whereas the two groups only rarely are found together in the Campanian–Maastrichtian of the Ibero-Armorican landmass (Le Loeuff et al. 1994). Additionally, small size seems to characterize the dinosaurs of the southern European Tethyan islands in the Santonian, similar to what has been noted for the Cenomanian and (in part) Turonian. Finally, the Santonian marks the first appearance of derived ceratopsians (ceratopsoids) in western Europe. These were considered descendants from earlier (Aptian–Albian) Asian immigrants by Godefroit and Lambert (2007; but see below, Discussions).

#### Campanian

Beginning with the Campanian, the European continental vertebrate fossil record becomes significantly better (Figs 1, 3, 4). This trend is obvious regardless of whether taxonomic diversity or chronostratigraphic or paleogeographic distribution of the record is considered. This is the result of substantial increases in exposed land area and corresponding numbers of continental vertebrate-bearing rock units (Smith et al. 2001; Smith and McGowan 2007; Benson et al. 2013). The increase in emergent land area towards the end of the Late Cretaceous was due to continuing convergence in the central-southeastern areas of Europe, and corresponding uplifts in the Carpathian, Balkan, and Dinaric orogens and surrounding areas (Dabovski et al. 2002; Schuller 2004; Otoničar 2007; Schmid et al. 2008; Venturini et al. 2008), as well as to the termination of spreading and initiation of convergence in the Pyrenean-Valais branch of the Alpine Tethys that affected southwestern Europe and marked the structuring of the Pyrenean Orogen (Sibuet et al. 2004). In more distant cratonic areas of Europe (Russia, Scandinavia) the occurrence of continental vertebrate remains is most likely related to sea-level drops, although these fossils are still restricted to near-shore marine deposits.

Campanian continental vertebrate fossils from Europe have been found across a large area, spanning from southern Sweden in the north to Slovenia and Valencia (Spain) in the south, and from Coimbra (Portugal) in the west to Saratov and Volgograd (Russia) in the east. Age constrains are fairly good for the near-shore marine localities or those located in successions showing marine influences, but are rather poor for those localities discovered in fluvio-lacustrine sediments deposited in purely continental settings. Therefore, the dating and correlation of most European vertebrate localities of this age is difficult (e.g., Buffetaut and Le Loeuff 1991a) and the age of many Ibero-Armorican localities is still given as late Campanian–early Maastrichtian (see Figs 1, 3 and sections **D**, **E** below).

Unequivocal Campanian (mainly early Campanian) continental vertebrates are known from Scania (southern Sweden), Villeveyrac in eastern Languedoc (southern France), Muthmannsdorf in Niederösterreich (eastern Austria), Sebeș in southwestern Transylvania (central Romania), and several sites in southeastern European Russia (Figs 1, 3, 4). The more important Austrian, French, and Romanian localities will be reviewed in detail in their respective dedicated sections below (see sections **C**, **D**, **F**).

In Sweden, lower Campanian shallow-marine deposits, which locally overlie upper Santonian–lowermost Campanian delta plain deposits, have yielded indeterminate theropod and ornithischian dinosaur remains (Weishampel et al. 2004). Recently, an indeterminate leptoceratopsid was reported from these deposits by Lindgren et al. (2007).

In southeastern Russia, the shallow-marine phosphate-bearing deposits of the Rybushka Formation yielded several isolated and fragmentary pterosaur specimens. Averianov (2007a) mentioned the presence of an indeterminate ornithocheirid from the Campanian of Volgograd Oblast. More common, however, are the remains of azhdarchid pterosaurs (including the genera *Bogolubovia* and *Volgadraco*), described from lower Campanian beds of Penza, Saratov, and Volgograd regions (Averianov 2007b, 2014; Averianov et al. 2008; Averianov and Popov 2014). An additional vertebrate occurrence was mentioned by Nessov (1995) from shallow-marine deposits of Volgograd Oblast, yielding bone fragments that were tentatively referred to indeterminate theropods, sauropods and thyreophorans. Nessov considered this locality Campanian–Maastrichtian, but this age was later given as Campanian by Averianov and Yarkov (2004a).

Vertebrate-bearing localities that are more coarsely constrained to the late Campanian–early Maastrichtian interval are spread throughout the Ibero-Armorican domain (Figs 1, 3, 4). These localities represent some of the most important Late Cretaceous continental vertebrate assemblages from Europe, and will be described in more detail in sections **D** and **E.** In southern France, they extend from the Department of Ariège, Languedoc, in the west, to the Var Department, Provence, in the east, along a wide belt stretching parallel with the Pyrenees and the Mediterranean coast (see section **D** and Figs 3, 4). On the Iberian Peninsula these localities are concentrated in north, in the Basque-Cantabrian and South Pyrenean regions, as well as along the centrally placed Iberian Range, with an interesting outlier on the Atlantic Coast, in the Lusitanian Basin, Portugal (see section **E** and Figs 3, 4).

Further to the east, an important although not exceptionally rich late Campanian–early Maastrichtian fossil locality was described from Villaggio del Pescatore in Trieste Province, northeastern Italy. Although first reported as Santonian (e.g., Dalla Vecchia 2001) or Santonian–Campanian (Dal Sasso 2003; Delfino et al. 2008b), the age of the site was later considered late Campanian–early Maastrichtian by Dalla Vecchia (2009a), based on biostratigraphic data and lithostratigraphic correlations. The fossils originate from calcareous deposits of the Liburnia Formation, formed in a restricted inner carbonate platform with freshwater influx. These shallow-marine beds succeed a bauxite-bearing erosional surface and its overlying freshwater limestones that mark a ‘middle’ Campanian emersion event in the Italian Karst region of the Adriatic-Dinaric Carbonate Platform (Venturini et al. 2008). The vertebrate assemblage of the site is dominated by the associated or articulated remains of *Tethyshadros*, a peculiar hadrosauroid close to the origin of the derived hadrosaurids (Dalla Vecchia 2009a). Besides *Tethyshadros*, the site has yielded articulated specimens of the eusuchian crocodyliform *Acynodon adriaticus* (Delfino et al. 2008b) as well as a few bones of indeterminate theropods (Dalla Vecchia 2001) and pterosaurs (Delfino et al. 2008b).

#### Maastrichtian

The paleogeographic-paleotectonic trends described above for the Campanian continue during the Maastrichtian. In western Europe, much of the Ibero-Armorican landmass emerged, concurrent with the westward withdrawal of the seaways in the north-Pyrenean and south-Pyrenean areas (e.g., López-Martínez et al. 2001; Laurent et al. 2002a). A similar marine regression is noted in the Carpathian orogenic areas in Romania (Vremir et al. 2014), and emergence, associated with karstification processes, is also reported from areas of the Adriatic-Dinaric Carbonate Platform (Otoničar 2007). As a result of this increase in terrestrial habitats and rock deposition, continental vertebrate-bearing Maastrichtian deposits are widespread in Europe. Vertebrate fossils have been reported from Portugal in the western part of the continent to Russia in the east, and from the Netherlands in the north to Valencia (Spain) and Bulgaria in the south (Figs 1, 3, 4).

The most important and best-studied Maastrichtian vertebrate localities are those from the Ibero-Armorican landmass (mainly southern France and north-central Spain) in western Europe and the Transylvanian landmass (northwestern Romania) in the east. These occurrences will be discussed in sections **D**–**F** (see also Figs 3, 4). Many of the Ibero-Armorican occurrences noted above (see *Campanian*) could be early Maastrichtian instead of late Campanian in age, but precise age constrains are still uncertain. Unequivocal Maastrichtian (mainly late Maastrichtian) occurrences are known from southwestern France (Figs 3, 4; section **D**), and northeastern and eastern Spain (Figs 3, 4; section **E**). In Romania, a Maastrichtian age is well documented for the largest part of the known latest Cretaceous vertebrate occurrences (Figs 3, 4; section **F**). Apart from the Ibero-Armorican and Transylvanian assemblages, Maastrichtian vertebrates are known from the Netherlands and Belgium, southern Germany, Slovenia, Bulgaria, Ukraine, and European Russia. Most of these fossils are found in shallow-water marine deposits, or from continental intercalations within dominantly marine deposits. As a result, the age of these remains is rather well constrained.

Chalk deposits from the Maastrichtian type area in Limburg (Belgium and the Netherlands) yielded some of the first continental vertebrate remains of this age from anywhere in the world (Seeley 1883). Although these are isolated skeletal elements that washed out into the sea, they are remarkably common and have been reported in surprisingly large numbers since the early discoveries (e.g., Buffetaut et al. 1985; Weishampel et al. 1999; Jagt et al. 2003; Mulder et al. 2005; Buffetaut 2009). One of the first specimens discovered was described as ‘*Megalosaurus*’ *bredai*, a ‘megalosauroid’ (basal theropod) by Seeley (1883). It was later reassigned to a new genus *Betasuchus* by Huene (1932). *Betasuchus* has long been considered either an ornithomimosaur (Huene 1932; Russell 1972) or a ceratosaur (Le Loeuff and Buffetaut 1991; Carrano and Sampson 2008). Recently, Carrano et al. (2012) accepted the ceratosaurian identification of the specimen. Theropod remains are by far surpassed in numbers, however, by those of hadrosaurids. Seeley (1883) coined the name ‘*Orthomerus dolloi*’ for the first hadrosaurid remains recovered from the Maastrichtian of Limburg. This taxon was considered a member of Hadrosauridae, albeit a *nomen dubium* in the recent review of the group by Horner et al. (2004); furthermore, Weishampel et al. (1999) argued for the possible presence of two different taxa of derived hadrosaurids.

New discoveries have revealed a more diverse Limburg fauna than previously recognized. Dyke et al. (2002, 2008) recently described definitive avian specimens from these deposits. Some of the fossils were interpreted as belonging to a primitive marine ornithurine similar to *Ichthyornis* (Dyke et al. 2002), but the specimens are too fragmentary to permit any definitive taxonomic assignment beyond Enantiornithes indet. and Ornithurae indet. (Dyke et al. 2008). Enantiornithines are thought to be a clade of exclusively continental birds, indicating that although the Limburg fossils are preserved in marine beds, they include land-living birds. Another newly reported and surprising occurrence in Limburg is that of a metatherian mammal, *Maastrichtidelphys*, reported by Martin et al. (2005). This taxon was originally described as a herpetotheriid metatherian by Martin et al. (2005) (see also Ladevèze et al. 2012), but in a comprehensive phylogenetic analysis Williamson et al. (2012, 2014) placed it instead in a large polytomy with pediomyid metatherians and many taxa traditionally referred to as “peradectids”, but distantly related to herpetotheriids. This taxon may indicate paleobiogeographic relationships (such as high-latitude dispersal routes) between eastern North America and western Europe during the Maastrichtian (Martin et al. 2005), but the fragmentary record of Cretaceous–Paleogene metatherians makes testing this biogeographic scenario difficult.

From Bavaria, in southern Germany, Wellnhofer (1994) reported the occurrence of isolated remains assigned to an indeterminate hadrosauroid from upper Maastrichtian deep marine (flyschoid) deposits of the Gerhartsreiter Schichten. The presumably associated remains of this small-bodied (under 2 m body length) individual seem to have been derived from an emergent area that existed on the submerged southern marginal areas of the European Craton during the late Maastrichtian.

Further to the south, an interesting association of continental vertebrates was recovered at Kozina (Kras, Slovenia) from a fissure fill developed in the uppermost Cretaceous limestone succession of the Liburnia Formation. Although the age of the assemblage was first tentatively considered early Campanian–late Maastrichtian (Debeljak et al. 1999, 2002), the geological setting suggests a late Maastrichtian age. The fossils were recovered from a karstic hole formed during a regional emergence of the northern part of the Adriatic-Dinaric Carbonate Platform that lasted from the ‘middle’ Campanian to the early-late Maastrichtian boundary (Otoničar 2007). Since the fossil-bearing karstic fissure fill is reported to contain clasts from the underlying shallow marine Lipica Formation (Santonian–Campanian) as well as clasts reminiscent of the overlying, freshwater limestones of the Liburnia Formation, the age of the fissure fill (and of the vertebrate remains therein) can be confidently refined to the late Maastrichtian. The assemblage reported from Kozina consists of fragmentary bone remains and isolated teeth (Debeljak et al. 1999). The teeth suggest the presence of one conical-toothed crocodyliform and possibly two different taxa of durophagous crocodyliforms (one of these maybe related to the hylaeochampsid relative *Acynodon*; Delfino et al. 2008b) as well as of theropod (possibly dromaeosaurid) dinosaurs (Debeljak et al. 2002). The assemblage is dominated by ornithopod teeth. Most of these can be referred to a rather derived hadrosauroid (maybe even a hadrosaurid), whereas others suggest the presence of a more basal, iguanodontian-grade ornithopod (Debeljak et al. 2002).

To the east, isolated dinosaur fossils were reported recently from late Maastrichtian limestones of the Kajlâka Formation, a unit deposited in an epicontinental sea bordering the northern margin of the Mediterranean Tethys (Jagt et al. 2006), over the southern margin of the cratonic Moesian Platform (Dabovski et al. 2002). These specimens suggest the presence of small-sized hadrosauroids (Godefroit and Motchurova-Dekova 2010) as well as, more surprisingly, possible ornithomimosaurs (Mateus et al. 2010). Once confirmed by additional discoveries, this latter record would represent the first occurrence of the clade in the Upper Cretaceous of Europe. European ornithomimosaurs are otherwise restricted to the upper Lower Cretaceous (Pérez-Moreno et al. 1994; Néraudeau et al. 2012; Allain et al. 2014). Although Mateus et al. (2010) suggested that the Santonian ‘*Megalosaurus*’ *lonzéensis* might also be an ornithomimosaur, this taxon was considered an indeterminate coelurosaur by Carrano et al. (2012).

A surprising late Maastrichtian vertebrate record, represented exclusively by dinosaur ichnites, comes from two sites identified in shallow-marine arenaceous limestones from the Roztocze area in southeastern Poland. Here, the Potok site yielded theropod footprints referred to *Irenesauripus*, as well as *Hadrosauropodus*-like ornithopod prints suggesting the presence of hadrosauroid dinosaurs (Gierliński et al. 2008). Subsequently, a more intriguing footprint assemblage was discovered in the nearby site of Mlynarka Mount (also known as the Szopowe quarry) (Gierliński 2009). The assemblage includes small-sized didactyl footprints referred to *Velociraptorichnus* sp. and attributed to an indeterminate dromaeosaurid. These are associated with a functionally tetradactyl footprint referred to *Macropodosaurus* sp., an ichnological morphotype considered to have been produced by an indeterminate therizinosauroid (e.g., Gierliński and Lockley 2013a), as well as with a similarly tetradactyl, but highly divaricated print identified as a small-sized specimen of *Saurexallopus*, an ichnotaxon usually linked to oviraptorosaurs (e.g., Gierliński and Lockley 2013b). Both of these latter identifications (especially if confirmed by discoveries of skeletal elements) would represent the first records of these two groups in the latest Cretaceous of Europe. The makers of the Polish tracks are considered inhabitants of the westernmost emergent areas of the wider Eastern European craton (Gierliński et al. 2008).

Hadrosauroid remains, referred to as ‘*Orthomerus weberae*’, were described by Riabinin (1945) from Upper Cretaceous shallow-marine deposits (glauconitic limestones) of the Besh-Kosh Mountains of Crimea. The taxon is considered a hadrosaurid, albeit a *nomen dubium*, by Horner et al. (2004). It represents an additional record of latest Cretaceous hadrosauroids from eastern Europe, interpreted as a lightly built, small-bodied hadrosaurid by Nessov (1995).

Finally, a few isolated continental vertebrate remains have been reported from lower Maastrichtian shallow-marine sands of the Volgograd Oblast (Russia). These fossils, associated with typical marine taxa such as sharks and mosasaurs, were referred to possible terrestrial turtles and to theropod dinosaurs (Averianov and Yarkov 2004a, 2004b). Although the turtle remains were cited as resembling the basal turtle *Kallokibotion* (Averianov and Yarkov 2004a), these similarities were dismissed in a recent review of central-eastern European turtles by Rabi et al. (2013a). The poorly preserved theropod remains were referred to a dromaeosaurid (isolated tooth) or a more basal, non-avetheropod taxon (fragmentary braincase) (Averianov and Yarkov 2004a), although the heavily worn state of the latter specimen casts some doubts on this referral and it might belong to an indeterminate ankylosaur instead (Averianov, pers. comm. 2014).

In summary, the most salient feature of the Maastrichtian fossil record of European continental vertebrates (excluding for the moment those from the better-sampled areas discussed below) is the widespread presence and numerical dominance of derived hadrosauroid (possibly hadrosaurid) dinosaurs. These are reported from both marginal settings of cratonic areas (Limburg, Bulgaria) and from more isolated, sea-bounded areas (Bavaria, Slovenia), with such occurrences being linked to emergent lands that existed within the Tethyan Realm. Furthermore, the great majority of these hadrosauroid remains appear to represent small-sized taxa. It is possible that this pattern is due to a taphonomic bias against larger fossils, or to the fact that most or all of the hadrosauroid specimens are juveniles. However, given that the small hadrosauroid fossils are numerically abundant and found across a wide geographic area and range of depositional settings, it is most likely that at least some Maastrichtian hadrosauroids of Europe were smaller than contemporary taxa in North America and Asia (see further discussion below). The occurrence of late-surviving ceratosaurians is also noteworthy, as is the suggested (but as yet weakly supported) presence of ornithomimosaurian, therizinosauroid, and oviraptorosaurian dinosaurs.

### B. Santonian, Hungary (Iharkút and Ajka)

#### History of research

Discovered in 2000, the Santonian-aged continental vertebrate locality at Iharkút is one of the most recent discoveries among the European Late Cretaceous sites. The locality is an abandoned and recultivated bauxite open-pit mine close to the villages of Németbánya and Bakonyjákó, in the heart of the Bakony Mountains, western Hungary (47°13'52"N, 17°39'01"E; Figs 1, 3, 4). The first fossils were found in the Sz-1 site, at the northen part of the open-pit, and additional bone-yielding horizons were later discovered in the Csehbánya Formation, among which the Sz-6 site at the southeastern end of the quarry is the most extensive (ca. 5000 m^2^) and productive. In the last 12 years, the Sz-6 site has yielded over 10,000 bones and teeth, including associated and articulated skeletons of both freshwater and continental vertebrates (Ősi et al. 2012b; Botfalvai et al. 2015; Fig. 5).

Besides the Iharkút locality, a few vertebrate remains, including teeth and some isolated bones, are known from the Ajka Coal Formation, which is stratigraphically equivalent with the Csehbánya Formation (Haas et al. 1992). These specimens were collected from the waste dumps of the subterranean Ajka coalmines in the Csinger Valley, close to the city of Ajka, which are 25 km from the Iharkút locality. A few years ago the coalmines were closed and the waste dumps were removed, and therefore there is no longer any access to these beds.

#### Geological setting

The Iharkút vertebrate locality is situated on a tectonic unit called the Transdanubian Range that was on the northern part of the triangular Apulian microfig between Africa and Europe during the Mesozoic (Csontos and Vörös 2004). The largest part of this block is formed by Triassic marine sediments, including the Upper Triassic Main Dolomite Formation that forms the thick basement of the Iharkút locality. Numerous 50-to-90-meter deep, tectonically controlled sinkholes on the karstified surface of this dolomite were filled by Cretaceous (pre-Santonian) bauxite. Palynological data (Knauer and Siegl-Farkas 1992; Bodor and Baranyi 2012), indirect nannoplankton data (Bodrogi and Fogarasi 1995), and paleomagnetic studies (Szalai 2005) indicate that the paleosurface formed by Triassic rocks and the accumulated bauxite lens were covered by fluvial and floodplain deposits of the Csehbánya Formation no later than the Santonian.

The Csehbánya Formation consists mainly of variegated clay, paleosol horizons, and silt with sand and sandstone layers, the latter interpreted as channel fills (Tuba et al. 2006; Ősi and Mindszenty 2009; Botfalvai et al. 2015). Vertebrate fossils are found in the coarse-grained, pebbly sandy basal beds (Sz-1, Sz-6 sites) of the fluvial half-cycles of the Csehbánya Formation, where the bones and teeth were washed together. Although isolated bones, teeth and plant remains appear in various stratigraphic horizons of the formation (including red paleosols and blackish, organic rich clay beds), the most productive sequence is a greyish coarse basal breccia layer (Sz-6 site) covered with sandstone and brownish siltstone. At some places in the open-pit mine, the Csehbánya Formation is unconformably covered by middle Eocene (Lutetian) conglomerates. In other parts of the Iharkút area, the Mesozoic sediments are covered by Oligocene clays, siltstones, sandstones, and conglomerates (Csatka Formation), or, in some places, by only a thin discontinuous blanket of Pleistocene loess.

The Ajka Coal Formation, which has yielded isolated teeth and bones, represents a swamp to forest-swamp facies formed at approximately the same time as the fluvial Csehbánya Formation, but deposited mainly west-southwest to it. It is composed of freshwater to brackish sediments including dark, clay-rich coal strata and pelitic calcareous and fine siliciclastic layers (Haas et al. 1992). Based on palynomorphs and correlated with nannoplankton zones, the Ajka Coal Formation is Santonian in age (Siegl-Farkas 1999).

#### Faunal overview

**Fishes.** Only two main groups, pycnodontiforms and lepisosteiforms, are known from Iharkút. Pycnodontiforms are represented by numerous isolated prearticulars with three to four tooth rows that are composed of elongate to circular teeth (Ősi et al. 2012b). Gulyás (2009) noted that these pycnodontiform remains belong to the genus *Coelodus* and they are among the few known freshwater occurrences of pycnodontiforms (Kocsis et al. 2009). The group has been reported from few other European Late Cretaceous vertebrate sites, besides the Lower Cretaceous Galve and Las Hoyas localities in Spain, providing evidence for its freshwater occurrence in Europe. Isolated pycnodontiform teeth have been also found in the Ajka Coal Formation.

Lepisosteiform remains consist of some jaw elements, numerous isolated teeth, vertebrae, and scales. Gulyás (2009) referred these remains to *Atractosteus*, which has been described from other European vertebrate faunas (Buffetaut et al. 1996; Grigorescu et al. 1999).

**Amphibians.** Amphibians are represented by both anurans and allocaudatans. Among anurans, the neobatrachian *Hungarobatrachus*, a specialized form with good jumping and swimming abilities (Venczel and Szentesi 2010), is unique to Iharkút (Szentesi and Venczel 2010). In addition to *Hungarobatrachus*, remains of the discoglossid *Bakonybatrachus* (Szentesi and Venczel 2012), as well as palaeobatrachid (Szentesi 2010) and pelobatid (Zoltán Szentesi, pers. comm., 2013) frogs have been recovered.

Although the available material (premaxillae, maxillae, dentaries) referable to Albanerpetontidae is not diagnostic enough to permit a more precise taxonomic assignment, the unusually large dimensions of some of the Iharkút dentaries suggests that two taxa may be present, including one new, large-sized taxon (Szentesi et al. 2013). In general, the albanerpetontid remains from Iharkút strongly resemble specimens from the Haţeg Basin of Romania, likely indicating close taxonomic affinities (Ősi et al. 2012b).

**Turtles.** Turtle remains, especially fragmentary shell pieces, are the most common fossils in Iharkút. More complete carapace and plastron remains, cranial and mandibular bones, and postcranial material (vertebrae, appendicular elements) are also relatively abundant. Turtle fossils can be assigned to Bothremydidae, Dortokidae, and meiolaniform ‘Kallokibotionidae’ (= ‘Kallokibotioninae’ of other authors, e.g., Rabi et al. 2013a), with the aquatic bothremydids being the most abundant.

Shell fragments of bothremydids from Iharkút indicate the presence of relatively large animals with body lengths of over one meter. Cranial features of the Hungarian bothremydid *Foxemys trabanti* (Fig. 5B) indicate its close relationship to *Foxemys mechinorum* (Tong et al. 1998) from the Upper Cretaceous of southern France (Rabi et al. 2012). Whereas bothremydid remains are known from various sites in southwestern Europe (Tong et al. 1998; Lapparent de Broin and Murelaga 1999; Murelaga and Canudo 2005) and also from Iharkút (Rabi and Botfalvai 2006), they have not been reported from the Maastrichtian of the Romanian Haţeg Basin (Grigorescu 2010a; Rabi et al. 2013a) or from the Campanian of Austria. Dortokidae, known by pelvic elements and shell material from Hungary (Ősi et al. 2012b), is a group endemic to Europe. It has been also reported from southern France and Spain (Pereda-Suberbiola 2009). *Kallokibotion*, first described from the Maastrichtian of the Haţeg Basin (Nopcsa 1923a, b) as representing an enigmatic lineage of basal cryptodires, is also known from Iharkút on the basis of a few shell fragments (Rabi et al. 2013a). A few turtle shell fragments have been also found in the blocks of the Ajka Coal Formation, but their precise taxonomic identity remains uncertain.

**Squamates.** In contrast to most Late Cretaceous continental vertebrate sites of Europe, remains of mosasaurs are frequently found at the Iharkút locality. *Pannoniasaurus inexpectatus* (Fig. 5A), a member of the basal mosasaur clade Tethysaurinae, is represented by a large number of individuals, including juveniles, which together preserve nearly all elements of the skeleton (Makádi et al. 2012). The largest specimens belong to animals with a total body length of approximately six meters, making them the top predators of the freshwaters of the Iharkút area during the Santonian. Stable isotope data measured from their teeth (Kocsis et al. 2009) suggests that, in spite of the primarily marine habitat of the group in other parts of the world, their occurrence in this lacustrine and fluvial environment was not occasional but reflects their normal habitat (Makádi et al. 2012). A fragmentary vertebra referred to *Pannoniasaurus* has also been found in the Ajka Coal Formation. The piece of rock that yielded this single vertebra contains abundant freshwater molluscs, typical of the lower part of the formation. Thus, multiple formations record the presence of *Pannoniasaurus* in freshwater environments, and indeed, there is no current evidence that it occurred outside of these environments (Makádi et al. 2012).

Similarly to the Haţeg fauna of Romania (Weishampel et al. 2010), one of the most diverse groups in the Iharkút fauna are the scincomorph lizards. Several different taxa can be distinguished based on numerous fragmentary mandibles and dentaries (Makádi 2008; Ősi et al. 2012b). *Bicuspidon* aff. *Bicuspidon hatzegiensis*, a small-bodied species first reported from the Haţeg Basin (Folie and Codrea 2005), is represented by numerous jaw elements from Iharkút (Makádi 2006; Fig. 3C). Aside from *Bicuspidon* aff. *Bicuspidon hatzegiensis*, another species of polyglyphanodontine lizard, *Distortodon rhomboideus*, has been also identifed from the Csehbánya Formation, increasing the known diversity of this group outside North America (Makádi 2013a). The Iharkút locality additionally provided the first evidence for a chamopsiid lizard outside North America: *Pelsochamops infrequens*, based on a partial mandible (Makádi 2013b).

**Crocodyliforms.** Based on isolated cranial material, four different crocodyliform taxa can be identified from Iharkút (Ősi et al. 2012b). First, the poorly known ziphodont taxon *Doratodon*, originally described from the lower Campanian Gosau Beds of Austria (Bunzel 1871; Buffetaut 1979), is represented by its diagnostic triangular, labiolingually flattened, and mesially and distally finely serrated teeth as well as some fragmentary dentaries (Rabi 2008; Rabi and Sebők in press). Recently, isolated fossils of this taxon have also been recorded from the Campanian of Spain (Company et al. 2005), the Maastrichtian of the Haţeg Basin (Martin et al. 2006), and possibly from Upper Cretaceous beds in Italy (Delfino 2001; but see above). A second non-eusuchian mesoeucrocodylian is known on the basis of fragmentary cranial remains and labiolingually compressed pseudoziphodont isolated teeth (Sebők et al. in prep.). The material appears to be closely related to *Theriosuchus* (Ősi et al. 2012b), an atoposaurid crocodyliform that survived from the Late Jurassic until the terminal Cretaceous in Europe (Martin et al. 2010).

Besides these small-bodied and probably mostly terrestrial crocodyliforms, two basal eusuchians are also known in the Iharkút fauna. The first, represented only by isolated cranial and mandibular elements as well as isolated teeth, shows close affinities to the medium-sized, semi-aquatic *Allodaposuchus* which has been reported from various European Campanian–Maastrichtian sites, including the Haţeg Basin, southern France, and perhaps Spain (Nopcsa 1928; Buscalioni et al. 2001; Delfino et al. 2008a; Martin 2010a). Some isolated crocodyliform teeth, appearing most similar to those of the *Allodaposuchus*-like form from Iharkút, have been also found in the Ajka Coal Formation.

The second eusuchian is the best-known crocodyliform of the Iharkút fauna: *Iharkutosuchus makadii*, a semi-aquatic basal hylaeochampsid eusuchian not exceeding one meter in length (Ősi et al. 2007). A great variety of cranial and mandibular remains are known for this taxon, including complete skulls (Fig. 5I), mandibles, and teeth of different ontogenetic stages, which provide insight into the paleobiology of this peculiar small-bodied eusuchian. This species possesses an extremely heterodont dentition with flat, multicusped grinding teeth, closed supratemporal fenestrae, and various other unusual cranial and mandibular features that were suggested to be related to a sophisticated jaw mechanism, dental occlusion, and oral food processing (Ősi 2008a; Ősi and Weishampel 2009).

**Pterosaurs.** Iharkút boasts one of the richest Late Cretaceous European pterosaur records, as it has produced numerous cranial and postcranial remains of azhdarchids. A new species of azhdarchid, *Bakonydraco galaczi*, was described from Iharkút based on a complete, edentulous mandible (Ősi et al. 2005; Fig. 5H). Besides the mandible, an elongate premaxillary tip and 54 additional mandibular symphyses has been also referred to this genus (Ősi et al. 2011). Such a great abundance of *Bakonydraco* jaw fragments clearly indicates that these animals were common in the Iharkút ecosystem. This abundance makes it probable that most of the pterosaurian postcranial remains from Iharkút, including numerous cervicals, pectoral girdle elements and limb bones, most probably belong to *Bakonydraco* as well. The material suggests an animal with an estimated wingspan of 3 to 4 meters.

There is, however, some evidence for additional pterosaurian taxa at Iharkút. Histological studies and statistical character analyses conducted on the large sample of mandibular symphyses indicate that the smallest specimens, which are three to four times smaller than the largest specimens of *Bakonydraco galaczi*, represent subadult-to-adult individuals. The identification of this material as representing mature or nearly mature individuals therefore suggests the presence of another taxon, which is probably also an azhdarchid (Prondvai et al. 2014). In addition, certain pterosaur bones could be referred only to Pterodactyloidea, among which the articular region of a mandible may suggest the occurrence of a taxon different from *Bakonydraco galaczi* (Ősi et al. 2011). It is currently unclear whether the small mandibular tips and this puzzling posterior mandible fragment (along with other problematic pterosaur remains) belong to the same taxon or represent different taxa.

**Dinosaurs: Ankylosaurs.** Iharkút is the only locality from the Late Cretaceous of Europe where remains of two different ankylosaurs have been found. The most abundant and best known is *Hungarosaurus tormai*, a medium-sized taxon (total length 4–4.5 meters) known by eight associated and one articulated partial skeletons as well as hundreds of isolated bones (Ősi 2005; Ősi and Makádi 2009; Fig. 5J). Phylogenetic analyses have provided strong support for its close relationship with another European ankylosaur, *Struthiosaurus*, which is known from most of the important latest Cretaceous (Santonian to Maastrichtian) European localities, including Iharkút (see below). Both taxa resolve as basal members of the clade Nodosauridae. It has been hypothesized that *Hungarosaurus* had a more sophisticated cerebral coordination of posture and movement, and perhaps a more cursorial habit, than other ankylosaurs (Ősi and Makádi 2009; Ősi et al. 2014b). This is based on the presence of a hypertrophied cerebellum, the gracile hindlimbs and forelimbs that are approximately equal in length (which is unusual for ankylosaurs), and the occurrence of paravertebral elements.

The presence of *Struthiosaurus* at Iharkút is indicated by a humerus that is smaller than and morphologically distinct from *Hungarosaurus*. This specimen demonstrates the sympatric existence of two different nodosaurid ankylosaurs: a smaller, robust form that was 2–2.5 meters in total length (*Struthiosaurus*) and a larger, cursorial form that was 4–4.5 meters in length (*Hungarosaurus*) (Ősi and Prondvai 2013). Clearly, ankylosaurs were an important component of the terrestrial fauna, filling a mid-sized herbivore niche, in the Santonian of Iharkút.

**Dinosaurs: Ornithopods.** In contrast to the latest Cretaceous sites of western Europe and Romania, rhabdodontid dinosaurs are among the rarest fossils in Iharkút, although they are present. A distinct species of rhabdodontid, *Mochlodon vorosi*, was described from Iharkút based on diagnostic features of the dentary (Ősi et al. 2012a; Fig. 5G). This species is the sister taxon of *Mochlodon suessi* from the Campanian of Austria. Cranial and postcranial features clearly distinguish *Mochlodon vorosi* from the western European *Rhabdodon* and from *Zalmoxes* of Romania. The *Mochlodon* species attained an adult total length of approximately 1.6–1.8 meters. Whereas the subadults of both *Zalmoxes* species were slightly larger (2–2.5 meters) than *Mochlodon*, the French specimens of *Rhabdodon* had a much larger (5–6 meter) adult length, indicating a substantial difference in body size between the western and eastern European taxa. Based on the distribution of femoral size on ornithopod phylogeny, it was shown that *Mochlodon* (estimated femur length of 245 mm) underwent some size reduction relative to the ancestral rhabdodontid condition (Ősi et al. 2012a). This phylogenetic analysis also implied a pre-Santonian divergence between western and eastern rhabdodontid lineages within the western Tethyan archipelago.

**Dinosaurs: Ceratopsians.** Although some controversial teeth and vertebrae from northwestern Europe have been assigned to ceratopsians (Godefroit and Lambert 2007; Lindgren et al. 2007; see above), the cranial remains of *Ajkaceratops kozmai* from Iharkút provided the first indisputable evidence of this clade in Europe (Ősi et al. 2010a; Fig. 5L). *Ajkaceratops* is very closely related to the Late Cretaceous bagaceratopsids (*Bagaceratops* and *Magnirostris*) from Central Asia and demonstrates that ceratopsians were widespread across the Northern Hemisphere, including the European archipelago, during the Late Cretaceous.

**Dinosaurs: Non-avian theropods.** Although their fossil material is scant, three different groups of non-avian theropods (basal tetanurans, abelisaurids, paravians) have been identified in the Iharkút vertebrate assemblage. Basal tetanurans are known from hundreds of isolated teeth, which are mostly 3-4 centimeters in length (Fig. 5D). These teeth are almost identical to specimens from the Campanian of Austria (‘*Megalosaurus pannoniensis*’) and ‘*Megalosaurus dunkeri*’ teeth from the Barremian of the Isle of Wight of England, suggesting the occurrence of late-surviving basal tetanurans in the Upper Cretaceous of Europe (Ősi et al. 2010b). These animals would have been the top predators in the Iharkút terrestrial ecosystem. A pedal ungual phalanx (Ősi et al. 2010b; Fig. 5E) and a right femur (Ősi and Buffetaut 2011) from Iharkút belong to the non-tetanuran theropod clade Abelisauroidea, thus further strengthening the earlier hypothesis (Buffetaut et al. 1988; Buffetaut 1989a) that these mostly Gondwanan theropods played a significant role in European Late Cretaceous faunas, where they would have been mid-sized (and in some cases perhaps large-sized) predators. Finally, the paravian record at Iharkút, which includes teeth and postcranial material, is more diverse than that of basal tetanurans and abelisauroids. Based on a single but highly diagnostic scapulocoracoid, a new small-bodied basal paravian theropod, *Pneumatoraptor fodori*, was identified (Ősi et al. 2010b; Fig. 5F). This taxon does exhibit some similarities with the Romanian dromaeosaurid *Balaur bondoc* (Csiki et al. 2010b) and other dromaeosaurids.

**Dinosaurs: Birds.** Approximately a dozen limb bones from Iharkút can be assigned to birds, some of which have been referred to Enantiornithes (Ősi 2008b). Among the enantiornithine bones, a complete tarsometatarsus was described as a new taxon, *Bauxitornis mindszentyae* (Fig. 5K), which shows great similarities with certain avisaurid taxa (Dyke and Ősi 2010).

### C. Lower Campanian, eastern Austria (Muthmannsdorf)

#### History of research

**Figure 6.**
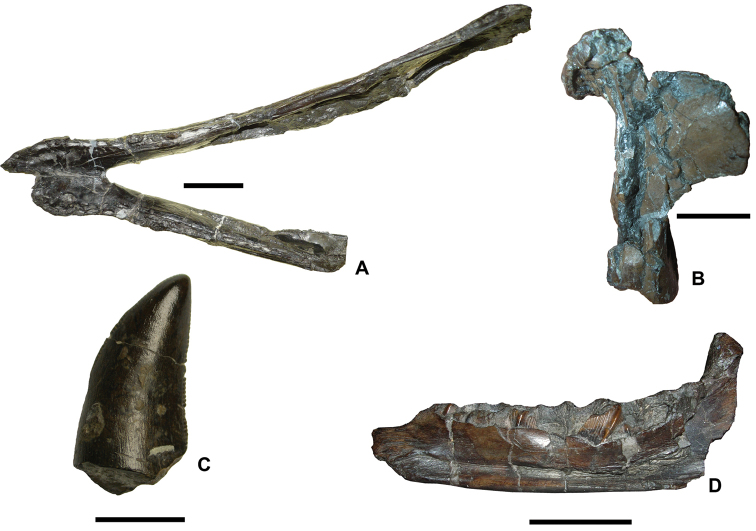
Representative taxa from the early Campanian Muthmannsdorf fauna from the Grünbach Formation, eastern Austria. **A** *Doratodon carcharidens* (Mesoeucrocodylia) mandible (PIUW 2349/57) in dorsal view (photo by Márton Rabi) **B** Indeterminate azhdarchid (Pterosauria, Azhdarchidae), left humerus (PIUW 2349/102) in anterior view **C** ‘*Megalosaurus pannoniensis*’ basal tetanuran (Theropoda, Tetanurae), tooth (PIUW uncatalogued) in lateral view **D** *Mochlodon suessi* (Ornithopoda, Rhabdodontidae), right dentary (holotype, PIUW 2349/2) in medial view. Scale bars equal 2 cm in **A, B** and **D** and 1 cm in **C.**

The first vertebrate fossil discovered in the coal-bearing beds at Muthmannsdorf, west of Wiener Neustadt (Lower Austria; Figs 1, 3, 4), was a dinosaur tooth found by Ferdinand Stoliczka in 1859, during an excursion led by Eduard Suess (Bunzel 1871). Subsequently, with the help of the mine manager Pawlowitsch, further excavations were conducted in the coal seam. This produced a small collection of vertebrate fossils (Seeley 1881; Fig. 6), which was first described by Bunzel (1870, 1871). Seeley (1881) revised the initial taxonomic identifications of Bunzel and gave new, more accurate descriptions for many of the species. After Seeley’s work, various authors reviewed some elements of this fauna (e.g., Buffetaut 1979, 1989b; Wellnhofer 1980; Pereda-Suberbiola and Galton 2001; Sachs and Hornung 2006; Buffetaut et al. 2011b). Because mining activity was stopped at the end of the nineteenth century, no additional bones have been found at Muthmannsdorf.

#### Geological setting

The vertebrate-bearing beds at Muthmannsdorf are part of the Upper Cretaceous to Palaeocene Gosau Group of the eastern Alps. These beds, composed mainly of marine to costal sediments (Summesberger et al. 2007), are placed within the Grünbach Formation of the Lower Gosau Subgroup (Summesberger et al. 2000). The Grünbach Formation is composed of alternating carbonaceous shales and coals with conglomerates and sandstones of freshwater or shallow-water marine origin (Summesberger et al. 2000). They were deposited on the northern corner of the Apulian microfig, relatively close to the Transdanubian Range (i.e. the Iharkút area of Hungary) (Csontos and Vörös 2004). Although Suess (1881) suggested a pre-Turonian age, more recent studies clearly demonstrated that the vertebrate-bearing beds are Campanian in age (Plöchinger 1961; Thenius 1974; Summesberger et al. 2007). More precisely, the Grünbach Formation was dated as early Campanian on the basis of foraminiferan (*Globotruncana elevata* Zone) and nannoplankton data (Zone UC 15) (Hradecká et al. 2000; Herman and Kvaček 2007).

#### Faunal overview

**Turtles.** Although turtle remains are relatively abundant in the Muthmannsdorf assemblage, they are exclusively shell fragments. After the initial description of Bunzel (1871), Seeley (1881) studied the turtle fossils and described them as belonging to a new species, *Emys neumayri*, based on two shell fragments. Nopcsa (1926) also examined the specimens and noted the occurrence of the characteristic Romanian taxon *Kallokibotion* in the Muthmannsdorf fauna but did not give any detailed explanation for this assignment. A recent overview of this material (Rabi et al. 2013a) listed the occurrence of dortokids and of possible ‘kallokibotionins’ (meiolaniforms) in the Muthmannsdorf fauna, strengthening the similarities with the Iharkút vertebrate fauna.

**Squamates.** A poorly preserved vertebra was identified as a lacertilian and named as a new taxon, *Araeosaurus gracilis* by Seeley (1881). However, no recent studies have confirmed the affinities of this specimen or identified it more precisely.

**Choristoderes.** Two platycoelous vertebral centra, originally assigned to dinosaurs by Bunzel (1871) and Seeley (1881), were reinterpreted by Buffetaut (1989b) as belonging to choristoderes. If this identification is correct then this is the first known Cretaceous record of the group in Europe. European choristoderes are known otherwise from the Triassic and Jurassic of the United Kingdom, as well as from Cenozoic deposits of Germany, France and the Czech Republic (Evans and Hecht 1993; Evans and Klembara 2005).

**Crocodyliforms.** A few specimens assigned to different crocodyliforms are known from Muthmannsdorf. Bunzel (1871) erected the species *Crocodilus carcharidens* based on a fragmentary mandible (Fig. 6A). This species was later reidentified as *Doratodon carcharidens* by Seeley (1881) who considered it a dinosaur. Subsequently, Nopcsa (1926) and Mook (1934) argued that *Doratodon carcharidens* was a crocodyliform as originally described. Redescription of the crocodyliform material of the Gosau Beds of Austria by Buffetaut (1979) listed this incomplete mandible, in addition a fragmentary right maxilla, a parietal fragment, and isolated teeth, as belonging to *Doratodon*, which is probably a sebecosuchian mesoeucrocodylian (Rabi and Sebők in press). In addition to *Doratodon*, an alligatorid eusuchian was identified on the basis of a mandible fragment and some postcranial material (Buffetaut 1979).

**Pterosaurs.** The pterosaur material from Muthmannsdorf consists of the articular region of a lower jaw, a proximal portion of a humerus (Fig. 6B), and crushed phalangeal fragments. Among these, Seeley (1881) described the mandible fragment as *Ornithocheirus bünzeli*. Subsequently, Wellnhofer (1980) named the humerus as *Ornithocheirus bünzeli* and referred the mandible to *Ornithocheirus* sp. A recent study of the Muthmannsdorf pterosaur remains (Buffetaut et al. 2011b) determined that the humerus is not from an ornithocheirid but rather from an azhdarchid pterosaur, a clade known from most latest Cretaceous European localitites. On the other hand, the mandibular fragment cannot be assigned to any specific pterodactyloid group due to the lack of diagnostic characters.

**Dinosaurs: Ankylosaurs.** Ankylosaur remains are the most abundant vertebrate fossils in the Muthmannsdorf assemblage. The material includes a few cranial elements and many isolated postcranial bones (vertebrae, limb bones, girdle and armor elements) of at least three different individuals. During the last 140 years, various authors have studied this material (Bunzel 1871; Seeley 1881; Nopcsa 1926, 1929; Pereda-Suberbiola and Galton 1992, 1994, 2001). The only clearly diagnostic element is a fragmentary basicranium referred to *Struthiosaurus austriacus* by Bunzel (1871). Later authors distinguished other taxa based on the postcranial remains, but the lack of autapomorphic features on these bones, however, means that they cannot be unequivocally assigned to a certain taxon (Pereda-Suberbiola and Galton 2001).

**Dinosaurs: Ornithopods.** A few dinosaur fossils, including both cranial and postcranial material, can be referred to a small-bodied ornithopod dinosaur. There has been substantial debate in the historical literature regarding this material: Bunzel (1871) described it as *Iguanodon suessii*, Seeley reinterpreted it as *Mochlodon suessi*, which Weishampel et al. (2003) regarded as a *nomen nudum*, and most later authors referred the specimens to *Rhabdodon* (e.g., Nopcsa 1915; Steel 1969; Brinkmann 1988; Pincemaille-Quillévéré 2002) whereas Sachs and Hornung (2006) assigned them to *Zalmoxes*. The latest interpretation of the Austrian ornithopod is related to the discovery and description of similar Hungarian rhabdodontid material, for which the genus name *Mochlodon* was resurrected with two valid species: *Mochlodon suessi* for the Austrian fossils (Fig. 6D) and *Mochlodon vorosi* for the Hungarian material (Ősi et al. 2012a).

**Dinosaurs: Non-avian theropods.** Two fragmentary teeth described as ‘*Megalosaurus pannoniensis*’ by Seeley (1881) belong to large carnivorous dinosaurs. The teeth (Fig. 6C) are almost identical to the large Iharkút theropod teeth referred to basal tetanurans (Ősi et al. 2010b).

### D. Santonian–Maastrichtian, Southern France

#### History of research

Fossil vertebrates were first reported from the Upper Cretaceous of southern France by Cuvier (1824), who mentioned crocodilian remains from the lignites of the Fuveau Basin in Provence. In the mid-nineteenth century, Matheron was the first to provide more detailed accounts of Late Cretaceous vertebrates from that area (Bouches-du-Rhône, Var), including dinosaurs (Matheron 1846, 1869). Farther west, in the foothills of the Pyrenees (Ariège), Pouech found vertebrate remains (including dinosaur eggs) in the 1850s (Le Loeuff 1992) but these finds attracted little attention. Gervais (1877) studied Late Cretaceous fossil eggs from southern France and briefly mentioned skeletal remains from Aude and Hérault. At the very end of the century, Depéret (1900) reported discoveries of Late Cretaceous vertebrates in the Saint-Chinian region of western Hérault, including ankylosaur material later described by Nopcsa (1929). A major review of the Late Cretaceous dinosaurs from southern France was published by Lapparent (1947), which remained the standard work on the topic for several decades, well after the identifications it contained had become outdated. In the 1950s, the abundant dinosaur eggs from the Aix-en-Provence area attracted a great deal of attention (e.g., Dughi and Sirugue 1957), but curiously little work was done at that time on skeletal remains from the same formations. It was only in the 1980s that interest in the dinosaurs of southern France was renewed, resulting in systematic excavations at various Late Cretaceous sites that continue to the present day.

#### Geological setting

**Figure 7.**
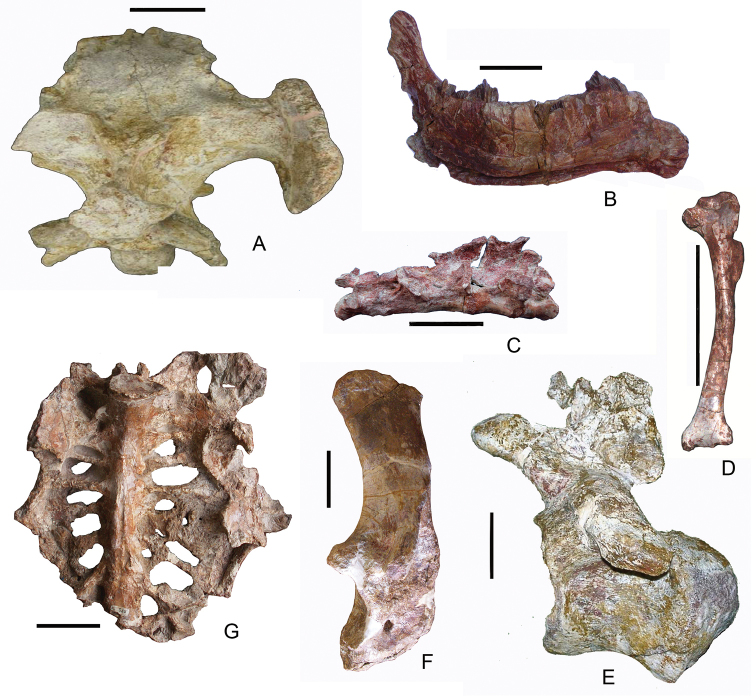
Representative taxa from the late Campanian–early Maastrichtian faunas from southern France. **A** *Arcovenator escotae* (Theropoda, Abelisauridae), braincase (MHNAix-PV 2011-12) in dorsal view (Lower Argiles Rutilantes Formation, Jas Neuf Sud, Var) **B** *Rhabdodon priscus* (Ornithopoda, Rhabdodontidae), left dentary (MC Mn 227) in lingual view (Grès à Reptiles Formation, Montplo Nord, Hérault) **C** *Variraptor mechinorum* (Theropoda, Dromaeosauridae), sacrum (MC PSP 6) in right lateral view (Grès à Reptiles Formation, Plo Saint-Pons, Hérault) **D** *Martinavis cruzyensis* (Aves, Enantiornithes), right humerus (MC M 1957) in caudal view (Grès à Reptiles Formation, Massecaps, Hérault) **E** Indeterminate titanosaur (Sauropoda, Titanosauria), caudal vertebra (MC M 0001) in left lateral view (Grès à Reptiles Formation, Massecaps, Hérault) **F** *Struthiosaurus* sp. (Ankylosauria, Nodosauridae), right scapulocoracoid (MC Mn 393) in lateral view (Grès à Reptiles Formation, Montplo Nord, Hérault) **G** *Gargantuavis philoinos* (Aves incertae sedis), synsacrum and part of ilia (MDE C3-525) in ventral view (Marnes de la Maurine Formation, Bellevue, Aude). All scale bars equal 50 mm.

The Late Cretaceous fossil vertebrates from southern France (Fig. 7) come from a fairly large number of localities in various sedimentary basins extending discontinuously over a large area (Figs 1, 3, 4). The western-most sites are in Haute-Garonne, where the transition from continental deposits to marine sediments deposited in a gulf of the Atlantic Ocean can be observed. The eastern-most occurrences are in Var (farther east, in Alpes-Maritimes, the Upper Cretaceous is represented by the marine deposits of the southern Alpine regions). In between, Upper Cretaceous vertebrate-bearing sediments occur in Ariège, Aude, Hérault, Gard, and Bouches-du-Rhône. The non-marine strata of these regions were deposited in different fluvial basins located in the central part of the Ibero-Armorican Island of the Late Cretaceous European archipelago. Fossils are found mainly in fluvial siltstones, sandstones, and conglomerates, and less frequently in lacustrine limestones and lignites. Vertebrate remains have usually undergone some transport, and articulated specimens are much less common than isolated bones and teeth. Relatively few formal formation names have been proposed for these Upper Cretaceous vertebrate-bearing rocks, although there are some formal designations for Provence (Cojan and Moreau 2006).

Dating the Late Cretaceous vertebrate localities of southern France has been, and still is, a major problem (Buffetaut and Le Loeuff 1991). Direct correlations with marine series are possible only in the westernmost area (especially Haute-Garonne). In other areas, such as Provence, it can only be determined that the oldest non-marine deposits (“Valdonnian”: see below) overlie marine rocks of Santonian age (Babinot and Durand 1980a), but no marine intercalations occur within the continental series, which in many places extend across the Cretaceous-Paleogene boundary, with the lowermost Paleogene deposits usually being represented by unfossiliferous red marls. There are no opportunities for radiometric dating of these deposits, which also yield few biostratigraphically useful fossils, although ostracodes, freshwater molluscs, charophytes, and fossil eggshells (Garcia and Vianey-Liaud 2001) have been used in attempts to date and correlate these rocks.

Several local stage names were proposed during the nineteenth century to subdivide the Upper Cretaceous continental series of Provence, notably by Matheron (1864: Valdonnian and Fuvelian) and Villot (1883: Begudian and Rognacian). They are, in ascending order, the Valdonnian, Fuvelian, Begudian, and Rognacian (Babinot and Durand 1980a,b,c,d). The Vitrollian (Matheron 1878; Babinot and Durand 1980e), which overlies the Rognacian, corresponds to the lower part of the Paleocene, spanning most of the Danian (Cojan and Moreau 2006). Because of the aforementioned dating problems, it has proven difficult to correlate these local stages with the standard global stratigraphic scale. Since the 1980s, magnetostratigraphy and chemostratigraphy have been used to complement biostratigraphy in an effort to better correlate the local stages. According to Cojan and Moreau (2006), the Valdonnian corresponds to part of the Santonian, the Fuvelian is upper Santonian to lower Campanian, the Begudian corresponds to the middle Campanian, and the Rognacian spans the upper Campanian and the entire Maastrichtian.

Outside Provence, the aforementioned local stages have sometimes been used, but correlations with the type areas are not straightforward. In the Pyrenees, the Garumnian local stage (Leymerie 1862) includes Upper Cretaceous (Maastrichtian) vertebrate-bearing beds, but extends into the Paleogene, up to the Thanetian (Plaziat 1980), and therefore is of little stratigraphic use.

A precise placement of most of the latest Cretaceous vertebrate localities in southern France on the standard stratigraphic scale is no easy task. Nevertheless, three main faunal complexes can be distinguished (Buffetaut et al. 1997b):

1. An older assemblage from the Fuvelian or contemporaneous deposits (notably in the Villeveyrac basin of Hérault), which is apparently early Campanian in age. Known localities have yielded mostly turtle and crocodile remains, with dinosaurs being less abundant.
2. An assemblage or group of assemblages from the “Begudo-Rognacian”, including the lower Rognacian as well, and therefore middle to late Campanian to early Maastrichtian in age. Most localities in southern France fall into that interval, notably in Var (Fox-Amphoux), Bouches-du-Rhône (Aix Basin), Hérault (Cruzy), and Aude (Campagne-sur-Aude). The most common dinosaurs are rhabdodontids and various titanosaurs. Ankylosaurs and theropods (abelisaurids and dromaeosaurids) are also relatively common.
3. A latest Cretaceous assemblage, corresponding to the later part of the Rognacian and thus late Maastrichtian in age. As noted by Le Loeuff et al. (1994), this assemblage is characterized by the abundance of hadrosaurids (which do not occur in the earlier faunal complexes), and the absence of rhabdodontids. Contrary to an earlier assumption (Le Loeuff et al. 1994), sauropods are not absent. Localities yielding this type of assemblage are mainly known from Haute-Garonne and Aude (Fontjoncouse).

A possible transitional assemblage, in which hadrosaurids and rhabdodontids occur together, has been reported from a single site at Vitrolles-la-Plaine (Bouches-du-Rhône), but reworking and mixing of specimens cannot be ruled out (Valentin et al. 2012).

#### Faunal overview

**Fishes.** Fish remains are found at many localities in the continental Upper Cretaceous of southern France. The most commonly encountered fossils are lepisosteid scales. More complete specimens include remains of *Atractosteus africanus* from the lower Campanian of Bouches-du-Rhône (Cavin et al. 1996). The presence of a freshwater mawsoniid coelacanth in the upper Campanian–lower Maastrichtian of Hérault is notable (Cavin et al. 2005). The upper Maastrichtian of Haute-Garonne has yielded a relatively diverse ichthyofauna (Laurent 2003).

**Amphibians.** Amphibian remains have been recovered by screenwashing from various sites belonging to all three aforementioned faunal complexes (e.g., Buffetaut et al. 1999; Garcia et al. 2000; Laurent 2003). They include albanerpetontids, caudatans, and anurans. Among the anurans, the presence of early Campanian palaeobatrachids is worth noting (Buffetaut et al. 1996), as is that of the late Campanian to early Maastrichtian batrachosauroidid salamaders (Buffetaut et al. 1997b).

**Turtles.** Turtles are common, and sometimes extremely abundant, at many localities. Bothremydid pleurodirans are represented in the early Campanian by *Polysternon* (Buffetaut et al. 1996), in the late Campanian–early Maastrichtian by *Foxemys* (Tong et al. 1998), and in the late Maastrichtian by *Elochelys* (Laurent et al. 2002b). Solemydid cryptodirans are also present at many localities (Buffetaut et al. 1999).

**Squamates.** Like amphibians, squamates have been recovered in some abundance at various sites where screenwashing has been performed (Buffetaut et al. 1999; Garcia et al. 2000; Laurent 2003). A recent review of the Late Cretaceous French squamate record was provided by Rage (2013), who noted (p. 522) that “the oldest faunas heralding terrestrial assemblages of modern type emerged in the Campanian”. Iguanids, scincomorphs, and possible amphisbaenians have been reported (but see Augé 2012). The large varanoid mentioned by Buffetaut et al. (1999) and Laurent (2003) apparently is a freshwater mosasaur rather than a terrestrial form (L. Makádi, pers. com. 2013).

**Crocodyliforms.** Crocodyliforms are commonly found at most localities and their diversity is high (Martin and Delfino 2010). In the early Campanian, they are represented in the “Fuvelian” deposits of Bouches-du-Rhône by *Massaliasuchus affuvelensis*, a basal alligatoroid (Martin and Buffetaut 2008). Crocodyliforms diversified during the late Campanian–early Maastrichtian interval. The largest taxon is *Ischyrochampsa meridionalis*, which is probably a eusuchian of uncertain affinities (e.g., Buscalioni et al. 2003) rather than a trematochampsid as originally suggested by Vasse (1995). *Allodaposuchus* is a mid-sized form, apparently a basal alligatoroid (Martin 2010a) or a basal eusuchian related to Hylaeochampsidae (Puértolas-Pascual et al. 2014). The smallest crocodilian in the assemblage is *Acynodon*, a small alligatoroid with a tribodont dentition (Martin and Buffetaut 2005). Diversity is lower for the late Maastrichtian. *Acynodon* is present in Haute-Garonne (Laurent 2003), as is the longirostrine eusuchian *Thoracosaurus* (Laurent et al. 2000).

**Pterosaurs.** Although not abundant, pterosaur remains have been found at various sites in the uppermost Cretaceous of southern France. Most of these are late Campanian to early Maastrichtian in age and are located in Aude, Hérault, and Var. All identifiable material is referrable to small or mid-sized azhdarchids (Buffetaut 2008). Late Maastrichtian pterosaurs are known from Aude and Ariège. The occurrence of a huge cervical vertebra at Mérigon (Ariège), indicating an azhdarchid with a wingspan of about nine metres, is particularly noteworthy (Buffetaut et al. 1997a).

**Dinosaurs: Ankylosaurs.** Nodosaurid ankylosaurs are present at various localities in southern France, but they are usually uncommon. They are known from the older “Fuvelian” faunal complex, notably in Provence (T. Tortosa, pers. com.) and at Villeveyrac (Hérault), where the type specimen of *Struthiosaurus languedocensis* was found (Garcia and Pereda-Suberbiola 2003). Ankylosaur remains are also known from several localities of late Campanian to early Maastrichtian age in Provence and Languedoc. The material from Quarante (Hérault) described by Nopcsa (1929) as *Rhodanosaurus ludgunensis* (usually considered a *nomen dubium*: Garcia and Pereda-Suberbiola 2003) belongs to that faunal complex. Newly discovered material from Cruzy (Hérault; Fig. 7F) may provide a better understanding of the ankylosaurs from that time interval. Ankylosaurs are poorly represented in the late Maastrichtian localities. Laurent (2003) reported a few nodosaurid dermal scutes from Lestaillats 1 (Haute-Garonne).

**Dinosaurs: Ornithopods.** Ornithopods are well represented in the older faunal complexes (early Campanian to early Maastrichtian) by rhabdodontids. *Rhabdodon* (Fig. 7B) was the first dinosaur to be identified in the Upper Cretaceous of southern France (Matheron 1869). Two species of *Rhabdodon* have been recorded, *Rhabdodon priscus* (Matheron 1869) and *Rhabdodon septimanicus* (Buffetaut and Le Loeuff 1991b). Although the validity of *Rhabdodon septimanicus* has been questioned (Allain and Pereda-Suberbiola 2003), the abundant material recovered during recent excavations, notably at Cruzy (Buffetaut 2005), confirms that two species are present.

In the youngest faunal complex (late Maastrichtian), rhabdodontids are no longer present and ornithopods are represented by hadrosaurids, known from localities in Aude and Haute-Garonne (Laurent et al. 2002a). According to a recent review by Prieto-Márquez et al. (2013), the French hadrosaurids can be referred to Lambeosaurinae. A large part of the material is identified only as Lambeosaurinae indet. However, the taxon *Canardia garonnensis* is based on various skeletal elements from the Tricouté 3 locality (Haute-Garonne). It is worth noting that *Canardia garonnensis* occurs in marine sediments only 1 meter below the iridium anomaly of the Cretaceous-Paleogene boundary at Larcan, Haute-Garonne, indicating that this taxon is one of the last Cretaceous continental vertebrates in Europe (Bilotte et al. 2010; Prieto-Márquez et al. 2013).

As noted above, the co-occurrence of rhabdodontids and hadrosaurids has been reported at a single locality in southern France, at Vitrolles-la-Plaine (Bouches-du-Rhône), which is considered late Maastrichtian in age (Valentin et al. 2012). This is an uncommon type of faunal assemblage that may represent a transitional stage between the earlier rhabdodontid-dominated assemblages and the later assemblages where only hadrosaurids are present. Alternatively, this unusual assemblage may be a result of reworking and mixing of specimens of different ages (Valentin et al. 2012). More research is currently needed to determine which of these explanations is correct.

**Dinosaurs: Non-avian theropods.** Abelisaurid and dromaeosaurid theropods are known from various sites in the uppermost Cretaceous of southern France, as are some more fragmentary fossils that may belong to small-bodied coelurosaurs.

Abelisaurids were first reported from the upper Campanian–lower Maastrichtian of Provence by Buffetaut et al. (1988). Although this identification was questioned (Allain and Pereda-Suberbiola 2003), new discoveries have confirmed the presence of these mid- to large-sized non-tetanuran theropods in southern France (Tortosa et al. 2014). The oldest Late Cretaceous remains are from “Fuvelian” (lower Campanian) deposits of Le Beausset (Var), which were described by Le Loeuff and Buffetaut (1991) as the new taxon *Tarascosaurus salluvicus*. Good cranial and postcranial material from the upper Campanian of Jas Neuf (Var) was assigned to another taxon, *Arcovenator escotae*, by Tortosa et al. (2014; Fig. 7A). Abelisaurids also occur at various other localities of late Campanian to early Maastrichtian age, notably at Cruzy (Hérault), and several taxa may be represented. The abelisaurid record for the late Maastrichtian is scant, although an isolated tooth from Cassagnau 1 (Haute-Garonne) described by Laurent (2003) may well belong to an abelisaurid. It is worth noting that much Late Cretaceous material from southern France referred to *Megalosaurus* in the older literature (e.g., Lapparent 1947) can now be referred to abelisaurids.

Isolated teeth referred to “deinonychosaurs” (now recognized as the clade including dromaeosaurids and troodontids) were first reported from Upper Cretaceous localities in southern France by Buffetaut et al. (1986). Subsequently, two dromaeosaurid taxa have been described from skeletal material from upper Campanian–lower Maastrichtian sites: *Variraptor mechinorum* from Var (Le Loeuff and Buffetaut 1998; Fig. 7C) and *Pyroraptor olympius* from Bouches-du-Rhône (Allain and Taquet 2000). Both taxa are based on incomplete postcranial material and their validity has been disputed (e.g., Turner et al. 2012). Following a description of additional material referrable to *Variraptor*, Chanthasit and Buffetaut (2009) concluded that comparison between the two named French dromaeosaurids was difficult because of the lack of substantial elements in common and that both taxa may be valid; it is also conceivable that they represent the same taxon. The late Maastrichtian record of dromaeosaurids is meager. Laurent (2003) reported teeth of indeterminate dromaeosaurids from Cassagnau 1 and 2 (Haute-Garonne).

Isolated teeth from the late Maastrichtian Vitrolles-la-Plaine locality (Bouches-du-Rhône) were assigned to the *Richardoestesia* morphotype by Valentin et al. (2012). Laurent (2003) referred an isolated tooth from the upper Maastrichtian of Marsoulas (Haute-Garonne) to ?*Euronychodon* sp. These tooth types are thought to pertain to derived paravian coelurosaurian theropods, although the lack of more complete skeletons in possession of these teeth makes identification extremely difficult (Currie et al. 1990; Sues and Averianov 2013).

**Dinosaurs: Birds.** Fossil birds are known from a few localities of late Campanian to early Maastrichtian age. Postcranial remains of enantiornithines have been reported from Cruzy, Hérault (Buffetaut 1998), and Fox-Amphoux, Var (Buffetaut et al. 2000). The enantiornithine taxon *Martinavis cruzyensis* is based on a humerus from Cruzy (Walker et al. 2007; Fig. 7D). The giant flightless bird *Gargantuavis philoinos* (Fig. 7G) is known from a few localities in Var, Aude and Hérault (Buffetaut and Le Loeuff 1998; Buffetaut and Angst 2013). The avian nature of *Gargantuavis* is not in doubt (Buffetaut and Le Loeuff 2010), but its exact affinities among birds are still uncertain. It may be an ornithuromorph close to ornithurines (Buffetaut and Angst 2013). The only late Maastrichtian avian specimen reported to date is a putative enantiornithine scapula from Haute-Garonne (Laurent 2003).

**Dinosaurs: Sauropods.** Matheron (1869) was the first to describe sauropod remains from the Upper Cretaceous of southern France, when he erected the taxon *Hypselosaurus priscus*—which he considered as a giant crocodile—on the basis of material from Bouches-du-Rhône. Depéret (1900) referred specimens from Hérault to *Titanosaurus*. Lapparent (1947) concluded that both *Hypselosaurus* and *Titanosaurus* were present at fossil localities in southern France, and various subsequent authors accepted this opinion. *Hypselosaurus* in particular was frequently associated with the abundant large eggs from Provence and other areas, often assumed to be the egg-layer. However, *Hypselosaurus* is now considered a *nomen dubium* (Le Loeuff 1993).

Recent research, based on both skeletal remains (Fig. 7E) and isolated teeth mostly from the late Campanian to early Maastrichtian interval, has revealed a relatively high diversity of titanosaurians in the uppermost Cretaceous of southern France (Díez Díaz et al. 2013d), with several taxa recognized. First, *Ampelosaurus atacis* is known from abundant material, including a partial articulated skeleton, from Aude (Le Loeuff 1995, 2005a). Second, *Atsinganosaurus velauciensis* was found in Bouches-du-Rhône (Garcia et al. 2010). Third, the as yet incompletely described “Massecaps titanosaur”, which exhibits an unusual dental morphotype, was recently found in Hérault (Klein et al. 2012; Díez Díaz et al. 2013d). It cannot be excluded that total titanosaur diversity was actually higher (Tortosa et al. 2012), as suggested by the co-occurrence of morphologically different elements at some sites that are currently difficult to assign to specific taxa (e.g., Cruzy, Aix Basin).

Sauropods were still present during the late Maastrichtian in southern France, although their diversity may have declined relative to the late Campanian to early Maastrichtian interval. Indeterminate titanosaur remains have been reported from Haute-Garonne (Laurent 2003) and Vitrolles-la-Plaine (Valentin et al. 2012).

**Mammals.** Mammal fossils are surprisingly uncommon in the Late Cretaceous sites of southern France, even where screenwashing has been conducted. Tabuce et al. (2013) reviewed the Late Cretaceous eutherian record from southern France and concluded that only three genera can currently be identified: *Labes*, *Valentinella* and *Mistralestes*. *Labes* can be definitively identified as member of the basal eutherian clade Zhelestidae, and the other two taxa might as well belong to the same group. It appears that metatherian mammals, which were present in the ‘middle’ Cretaceous of France (Vullo et al. 2009b) as well as in the Maastrichtian of Limburg (Martin et al. 2005; see section **A**) and common in the latest Cretaceous of North America (Wilson 2013), were apparently absent in the uppermost Cretaceous of southern France.

### E. Campanian–Maastrichtian, Spain and Portugal

#### History of research

Latest Cretaceous (Campanian–Maastrichtian) dinosaur remains have been known from the Iberian Peninsula since the end of the 19th century (Figs 3, 4). In Portugal, Sauvage (1897-98) described theropod teeth from the “Garumnian” of Viso (formerly Vizo), near Coimbra, together with associated remains belonging to actinopterygians, amphibians, turtles, and crocodyliforms. The Viso assemblage was reviewed by Lapparent and Zbyszewski (1957). Subsequently, two other vertebrate sites of the same age, Aveiro and Taveiro, were found in what was then the Beira Litoral province (Antunes and Pais 1978; Antunes and Broin 1988; Antunes and Sigogneau-Russell 1991, 1992; Galton 1994, 1996; Antunes and Mateus 2003: map in fig. 16). These sites, which have also yielded selachian and mammalian teeth, are regarded as Late Campanian to Maastrichtian in age.

**Figure 8.**
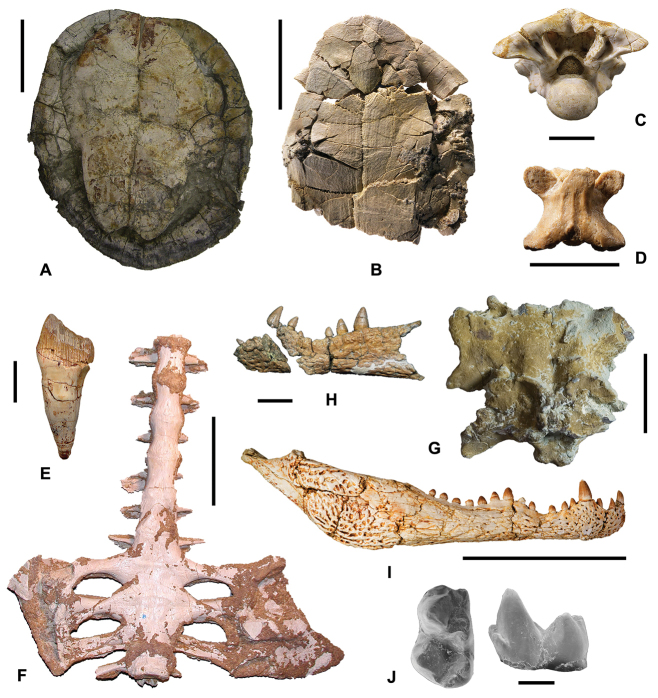
Representative taxa from the late Campanian–early Maastrichtian faunas from Spain. **A** *Iberoccitanemys convenarum* (Pleurodira, Bothremydidae), complete shell (HUE-4913) in ventral view (Villalba de la Sierra Formation, Lo Hueco near Fuentes, Cuenca) **B** *Dortoka vasconica* (Pleurodira, Dortokidae), partial shell (holotype, MCNA 6313) in ventral view (unnamed unit, Laño, Condado de Treviño) **C** *Menarana laurasiae* (Serpentes, Madtsoiidae), mid-trunk vertebra (holotype, MCNA 5337) in posterior view (unnamed unit, Laño, Condado de Treviño) **D** *Herensugea caristiorum* (Serpentes, Madtsoiidae), mid-trunk vertebra (holotype, MCNA 5387) in dorsal view (unnamed unit, Laño, Condado de Treviño) **E** *Rhabdodon* sp. (Ornithopoda, Rhabdodontidae), maxillary tooth (MGUV CH-162) in labial view (Sierra Perenchiza Formation, Chera, Valencia) **F** *Struthiosaurus* sp. (Ankylosauria, Nodosauridae), synsacrum (MCNA 7420.1) in ventral view (unnamed unit, Laño, Condado de Treviño) **G** *Ampelosaurus* sp. (Sauropoda, Titanosauria), braincase (HUE-8741) in dorsal view (Villalba de la Sierra Formation, Lo Hueco near Fuentes, Cuenca) **H** *Doratodon ibericus* (Crocodyliformes, Ziphosuchia), left dentary (holotype, MGUV 3201) in lateral view (Sierra Perenchiza Formation, Chera, Valencia) **I** *Musturzabalsuchus buffetauti* (Crocodyliformes, Eusuchia), right mandible (paratype, MCNA 7480) in lateral view (unnamed unit, Laño, Condado de Treviño) **J** *Lainodon orueetxebarriai* (Eutheria, Zhelestidae), first lower molar (holotype, MCNA L1AT 14) in occlusal and labial views (unnamed unit, Laño, Condado de Treviño). Scale bars equal 10 cm (**A, F, I**), 5 cm (**B, G**), 2 cm (**H**), 1 cm (**C, E**), 5 mm (**D**), 1 mm (**J**). Photographs courtesy by Adán Pérez-García (**A**), J. Carmelo Corral (**B–D**), Julio Company (**E, H**), GBE-UNED/MCCM (**G**), Francisco Ortega (**I**) and Emmanuel Gheerbrant (**J**).

**Figure 9.**
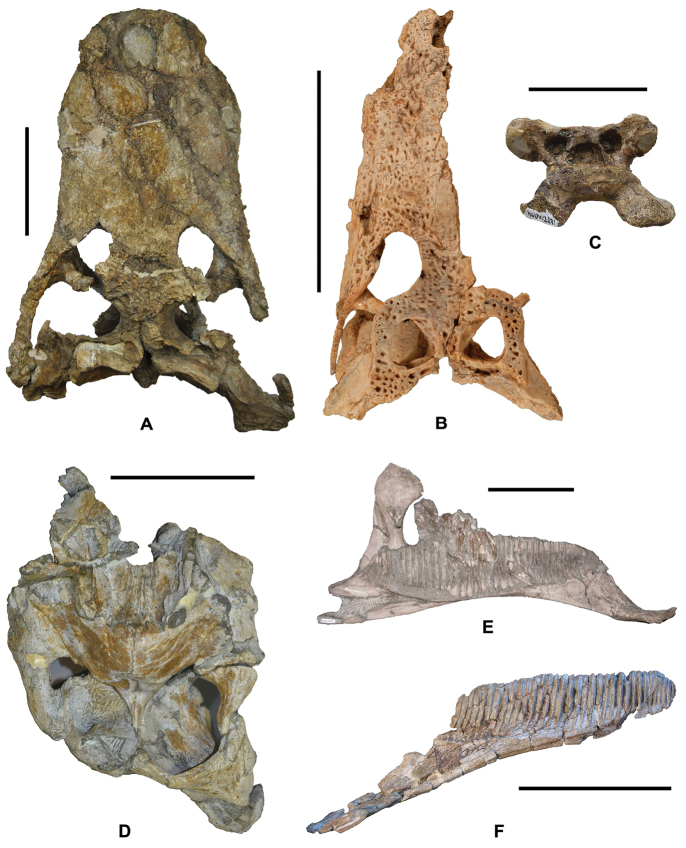
Representative taxa from the late Maastrichtian faunas from Spain. **A** *Allodaposuchus subjuniperus* (Crocodyliformes, Eusuchia), skull (holotype, MPZ 2012/288) in dorsal view (lower Tremp Formation, Beranuy near Arén, Huesca) **B** *Arenysuchus gascabadiolorum* (Crocodyliformes, Eusuchia), skull (holotype, MPZ ELI-1) in dorsal view (lower Tremp Formation, Arén, Huesca) **C** Indeterminate azhdarchid (Pterosauria, Azhdarchidae), cervical vertebra (MGUV 2271) in posterior view (unnamed unit (Margas de los Cuchillos Formation?), La Solana near Tous, Valencia). **D–E** *Arenysaurus ardevoli* (Ornithopoda, Lambeosaurinae) **D** partial skull (holotype, MPZ 2008/1) in dorsal view and **E** left dentary (paratype, MPS 2008/258) in medial view (basal Tremp Formation, Blasi 3, Arén, Huesca) **F** ‘*Koutalisaurus kohlerorum*’ (Ornithopoda, Lambeosaurinae; indeterminate lambeosaurine sensu Prieto-Márquez et al. 2013), right dentary (IPS 29920 (formerly IPS SRA 27) in medial view (‘lower red unit’ of the Tremp Formation, Les Llaus near Sant Romà d’Abella, Lleida) **F** Scale bars equal 10 cm (**A–B, D–F**), and 5 cm (**C**). Photographs courtesy by Museo de Ciencias Naturales de la Universidad de Zaragoza (**A–B, D**), Julio Company (**C**) and Alberto Prieto-Márquez (**E–F**).

In Spain, the first dinosaur remains from uppermost Cretaceous formations were found during the 1920s in the Tremp area of Lleida in the Catalonian Pyrenees, but systematic field research on these sites was not undertaken until the 1950s (Lapparent and Aguirre 1956). Other isolated finds were also made near Soria around the same time (Lapparent et al. 1957). In the last thirty years, systematic excavations at a large number of sites distributed across the Iberian Peninsula, mainly in the southern Pyrenees (provinces of Huesca, Lleida, and Barcelona), the Iberian Range (mainly Burgos, Cuenca, Segovia, and Valencia), and the Basque-Cantabrian Region (Condado de Treviño within Alava), have yielded abundant fossils of dinosaurs and other continental vertebrates (Pereda-Suberbiola et al. 1999b; Pereda-Suberbiola 2006; López-Martínez et al. 2001; Ortega et al. 2006 and references therein; Figs 8, 9).

#### Geological setting

Most of the Campanian–Maastrichtian vertebrate sites from the Iberian Peninsula are located in the southern Pyrenees, specifically in the Àger, Tremp, Coll de Nargó, and Vallcèbre synclines, which are in the provinces of Huesca (Aragón community), Lleida, and Barcelona (Catalonia) from west to east. Two main geological units concentrate the uppermost Cretaceous deposits: the shallow marine-deltaic Arén Sandstone and the transitional-to-continental Tremp Formation, also commonly known as the local “Garumnian” (López Martínez et al. 2001; Riera et al. 2009; López-Martínez and Vicens 2013; Vila et al. 2013a). These diachronous formations range in age from late Campanian to late Maastrichtian (Arén Sandstone), respectively from late Campanian to early Paleogene (Tremp Formation).

Numerous fossil localities in the Arén Sandstone and Tremp Formation have yielded bones, footprints, and eggs attributed to different groups of dinosaurs (López-Martínez et al. 2001; López-Martínez 2003a; Oms et al. 2007; Pereda-Suberbiola et al. 2009b; Vila et al. 2012, 2013b; Prieto-Márquez et al. 2013; Sellés et al. 2013, 2014a). Dinosaur fossils occur in various depositional settings, including inland fluvial systems and coastal (lagoonal, palustrine) environments (Ardèvol et al. 2000; López-Martínez et al. 2009; Vila 2013; Vila et al. 2013a). Two particularly important dinosaur-dominated vertebrate sites are the Tremp-Isona outcrops of Lleida (Tremp Formation) and the Blasi localities near Arén in Huesca, which exposes delta-front, lagoonal, and coastal deposits (Arén Sandstone). Furthermore, late Maastrichtian dinosaur nesting sites yielding clutches, eggs, and eggshells are known from Lleida and Barcelona; these are considered to be among the most important recorded in Europe (Vila et al. 2011; Sellés et al. 2013).

Other important latest Cretaceous vertebrate-bearing localities are found in the Basque-Cantabrian region of Spain (north-central Iberian Peninsula). The best known of these is Laño in the Condado de Treviño, an enclave of Alava administered by the province of Burgos. The fossiliferous beds of the Laño quarry have yielded one of the most diverse vertebrate assemblages of Europe, which consists of nearly 40 species, including dinosaurs, crocodyliforms, snakes, turtles, and mammals (Astibia et al. 1990, 1999; Pereda-Suberbiola et al. 2000, submitted). The fluvial beds of Laño are regarded as late Campanian to early Maastrichtian in age on the basis of stratigraphical correlations (Berreteaga 2008). Other latest Cretaceous vertebrate localities are known in Alava and northern Burgos (Pereda-Suberbiola et al. 1999; Lécuyer et al. 2003 and Corrigendum).

In the Iberian Range of Spain, latest Cretaceous vertebrate sites are located in several areas, from the Demanda-Cameros region in the northwest to the Cuenca and Valencia provinces in the southeast. In Burgos, on the southern margin of the Cameros Massif, the lacustrine “Lychnus Limestone” unit—which corresponds to the lower part of the Santibañez del Val Formation (Maastrichtian)—has yielded a collection of vertebrate remains, including teeth of crocodyliforms (Buscalioni et al. 1997) and mammals (Pol et al. 1992). Dinosaur bones and eggshells have also been found in the same unit near Arauzo de Miel in Burgos (Torcida 1996; Bravo et al. 2006). In Soria, fluvial clays of the Santibañez del Val Formation (“Garumnian” facies) have yielded a limited amount of dinosaur fossils (Lapparent et al. 1957; Pereda-Suberbiola and Ruiz-Omeñaca 2001).

In Cuenca Province of central Spain (southeastern Iberian Range), the most important locality is “Lo Hueco” near Fuentes. This site, where more than 10,000 fossils have been collected, is regarded as a Konzentrat-Lagerstätte (Ortega et al. 2008, submitted, and references therein). The “Garumnian” facies of Lo Hueco represents a near-coast muddy floodplain (palustral) environment (Barroso-Barcenilla et al. 2009). The flora and invertebrate and vertebrate fauna of this site suggest a late Campanian to early Maastrichtian age. Work continues at Lo Hueco and many new vertebrate taxa should be described from this site in the near future (e.g., Pérez-García et al. 2012a).

In Valencia Province of eastern Spain (southeastern margin of the Iberian Range), two vertebrate sites are particularly diverse and important: Chera and La Solana in the municipality of Tous (Company 2004). The fossiliferous beds of Chera are interpreted as deposits of small ephemeral carbonate lakes and ponds in a coastal environment (Company 2004). The vertebrates found at this site are similar to those found at Laño in the Basque-Cantabrian Region (Company et al. 2009b). The fossiliferous horizons of La Solana were accumulated in a distal alluvial floodplain environment, and the vertebrates here are different than those from Chera (Company et al. 1998, 1999, 2013); the La Solana beds are regarded as latest Maastrichtian in age.

In the Central Range of Spain, the Armuña site in Segovia Province has yielded a vertebrate assemblage that is similar to, but less diverse than, those of Laño and Chera (Buscalioni and Martínez Salanova 1990; Corral Hernández et al. 2007). The fossiliferous beds, which are considered to be broadly Campanian to Maastrichtian in age, were formed in a braided river system flowing along or near a coastal plain (Gil et al. 2010).

Finally, in Portugal, the sites of Aveiro, Taveiro, and Viso near Coimbra have yielded a diverse vertebrate assemblage of late Campanian to Maastrichtian age (Antunes and Broin 1988). Dinosaurs are mainly represented by isolated small teeth (Antunes and Sigogneau-Russell 1991, 1992). Antunes and Mateus (2003) interpreted the absence of large-sized dinosaurs in this fauna as reflecting a sharp decrease in the diversity of large-bodied taxa relative to previous time intervals (see also Antunes and Sigogneau-Russell 1996), but the apparent absence of large vertebrates is more likely due to preservational factors.

#### Faunal overview

**Fishes.** Continental vertebrate sites from the Campanian–Maastrichtian of the Iberian Peninsula have yielded rare actinopterygian fossils, including lepisosteiforms (gars) and teleosteans (Cavin 1999). Their remains include mainly rhomboidal ganoid scales, teeth with plicidentine, opisthocoelous vertebrae, and some bones.

Much of the Spanish actinopterygian material is assignable to freshwater lepisosteids. Cavin (1999) referred the lepisosteid remains of Laño to *Atractosteus*, but reinterpretation of the material based on new data (Grande 2010) indicates that it can only be attributed more generally to Lepisosteidae indet. (Pereda-Suberbiola et al. submitted). Lepisosteid remains have also been reported from the Blasi 2 site in Arén (Blain et al. 2010), Lo Hueco (Serrano et al. 2012), and Tous (Company et al. 2013). Indeterminate actinopterygians represented by amphicoelous vertebrae are known from Lo Hueco (Ortega et al. submitted).

Among teleosteans, the occurrence of Phyllodontidae and Palaeolabridae in the Upper Cretaceous of Europe was first recorded in Laño (Cavin 1999). Subsequently, additional phyllodontid-like teeth have been found in other Iberian continental localities, including La Solana (Company et al. 2013). Pycnodontiforms and osteoglossids may also be present at La Solana (Company et al. 2013). At Lo Hueco, teeth of Pycnodontoidea, Amiidae, and Albulidae have been reported (Torices et al. 2011).

Finally, teeth of small, durophagous osteichthyans and a few scales have been found at the Molí del Baró-1 and Barranc de Torrebilles sites in Lleida, which represent oxbow lake and meandering river deposits, respectively (Marmi et al. 2012a). It is not clear to which group of bony fishes these unusual crushing teeth belong.

**Amphibians.** Fossils of amphibians, including albanerpetontids and anurans, have been recovered by screenwashing from a small number of Campanian–Maastrichtian Iberian sites. These include Laño (Duffaud and Rage 1999), Arén (Blain et al. 2010), Chera and Tous (Company et al. 2009b; Company and Szentesi 2012), and the Beira Litoral sites of Portugal (Antunes and Broin 1988). Among the anurans, discoglossids and palaeobatrachids were reported, along with possible pelobatids (Company and Szentesi 2012).

The amphibian assemblage of Laño is one of the richest and most diverse from the Upper Cretaceous of Europe (Duffaud and Rage 1999; Duffaud 2000). It consists of about 200 isolated bones belonging to at least five taxa: an indeterminate albanerpetontid, an indeterminate caudatan (which may be one of the oldest known records of salamandrids), and at least three anurans (Duffaud and Rage 1999; Duffaud 2000).

In Arén, the Blasi 2 site has also yielded a relatively diverse amphibian fauna, which includes the disarticulated fossils of one albanerpetontid and two anurans. The albanerpetontid is remarkably similar to *Albanerpeton nexuosum* from the Campanian–Maastrichtian of North America (Blain et al. 2010) and may represent the same taxon or a close relative. The two anurans are differentiated by pelvic traits: the first taxon has small ilia matching those of discoglossids, the second has larger ilia similar to those of palaeobatrachids. The discoglossid from Blasi 2 is also represented by other bones, which exhibit similarities with the North American *Paradiscoglossus americanus* as well as with material from Laño (Duffaud and Rage 1999) and the Haţeg Basin of Romania (Folie and Codrea 2005). Palaeobatrachid remains are much more abundant than those of discoglossids at Laño, whereas discoglossids are the dominant taxa at Blasi 2. This difference may be linked to the paleoenvironment (Blain et al. 2010): Laño represents a freshwater fluvial environment (Pereda-Suberbiola et al. 2000) whereas Blasi 2 may correspond to a coastal, mangrove-like swamp (Ardèvol et al. 2000).

The amphibian records of other Iberian sites are poor. Fragmentary remains of indeterminate albanerpetontids, discoglossids, and possibly pelobatids have been reported from Chera (Company and Szentesi 2012). At La Solana, these groups are found together with palaeobatrachids (Company et al. 2013). Finally, indeterminate anurans have been reported from other localities, such as the Fontllonga and Molí Vell sites in Lleida (López-Martínez et al. 1999; Rocek 2000).

**Squamates.** Squamates from the Campanian–Maastrichtian of the Iberian Peninsula include specimens of lizards and snakes. Squamate fossils mostly comprise maxillary and dentary fragments, teeth, and vertebrae, and have usually been collected by screenwashing and microfossil picking. They are relatively abundant at various localities, mainly Aveiro, Laño, Chera, Lo Hueco, and Blasi 2 in Arén (Antunes and Broin 1988; Rage 1996, 1999; Blain et al. 2010; Narváez and Ortega 2010; Torices et al. 2010).

Lizards comprise non-acrodontan iguanians (i.e., Iguanidae *sensu lato*) and scincomorphans. Laño was the first Iberian locality to document the presence of pleurodont Iguanidae (Rage 1999), which subsequently have also been reported from Lo Hueco (Narváez and Ortega 2010). Remains of indeterminate lizards, which could belong to iguanids, have also been described from Blasi 2 (Blain et al. 2010). Among scincomorphans, one isolated tooth found at Laño may belong to Paramacellodidae (Rage 1999), a group reported from the uppermost Cretaceous of Central Europe (see below, section **F**). Vertebrae from Laño and Blasi 2 that may potentially belong to amphisbaenians, but more probably represent anguids, would be the earliest records of one or both of these clades in Europe (Astibia et al. 1990; Rage 1999; Blain et al. 2010). Varanoid vertebrae have been recovered from Aveiro, Laño, and Lo Hueco, with the latter material probably belonging to a new taxon of a non-marine pythonomorph (Houssaye et al. 2013).

Snake fossils are among the least common specimens from the Iberian vertebrate sites. Two madtsoiid snakes are present at Laño, both of which are only known from this locality: *Menarana laurasiae* (Fig. 8C) and *Herensugea caristiorum* (Fig. 8D) (Rage 1996, 1999; LaDuke et al. 2010). The genus *Menarana* is known elsewhere only from the Maastrichtian of Madagascar (LaDuke et al. 2010). Together with *Nidophis insularis* from the Hațeg Basin of Transylvania, the Laño snakes seem to exhibit affinities with characteristic Gondwanan taxa (LaDuke et al. 2010; Vasile et al. 2013). An indeterminate, possible alethinophidian snake has also been reported from Blasi 2, but its affinities are uncertain (Blain et al. 2010).

One problematic specimen deserves brief comment. Recently, Apesteguía (2012) suggested the presence of an eilenodontine sphenodontian in Laño on the basis of a fragment of maxilla or dentary with teeth, but its assignment to an indeterminate lacertilian cannot be ruled out (Rage 1999).

**Turtles.** Turtle fossils are one of the dominant elements in the Late Cretaceous vertebrate assemblages. Representatives of three groups have been recognized in the Campanian–Maastrichtian of the Iberian Peninsula, two of them assigned to Pleurodira (Bothremydidae and Dortokidae) and one to stem Testudines (Solemydidae).

Bothremydids are the most abundant and diverse turtles from the Iberian localities overall. They are represented by *Rosasia soutoi* at Aveiro, Taveiro, and Viso (Antunes and Broin 1988); *Polysternon atlanticum* at Laño (Lapparent de Broin and Murelaga 1996, 1999); and *Iberoccitanemys convenarum* at Lo Hueco (Pérez-García et al. 2012a; Fig. 8A). All but the Portuguese taxon *Rosasia* are members of Foxemydina, a clade known only in the Santonian to Maastrichtian of Europe (Gaffney et al. 2006; Pérez-García et al. 2013). A turtle from Isona in Lleida, described as *Polysternon isonae* by Marmi et al. (2012b), may be a distinct taxon or may be diagnostic only at the level of Foxemydina indet. (Pérez-García 2012a). Moreover, indeterminate bothremydids have been reported from several other Iberian localities (see list in Pérez-García et al. 2013).

Dortokidae is an endemic European lineage of pleurodirans. The best-known Iberian dortokid is *Dortoka vasconica* from Laño (Lapparent de Broin and Murelaga 1996, 1999; Fig. 8B). The presence of several morphological features, including large fontanelles on its carapace, indicates that *Dortoka* was a freshwater turtle (Pérez-García et al. 2012b). Dortokid remains are also known from Chera and probably from Armuña in Segovia.

Solemydids have a unique shell sculpturing that consists of distinct tubercles, making their fossils easy to identify. These turtles may have had terrestrial habits (Joyce et al. 2011). Iberian solemydids are represented by *Solemys vermiculata* from Laño (Lapparent de Broin and Murelaga 1996, 1999) and *Solemys*-like remains from other localities, including Armuña, Chera, and Arén (Pérez-García 2009, 2012a). *Solemys* is comparatively larger in size (carapace length ~70 cm) than the Iberian bothremydids and *Dortoka*.

**Crocodyliforms.** Eusuchians are the major components of the Late Cretaceous crocodyliform assemblages from Europe and are represented in Spain by a variety of forms, including the alligatoroids *Acynodon iberoccitanus* and *Musturzabalsuchus buffetauti* (Fig. 8I) from Laño (Buscalioni et al. 1997, 1999), the basal eusuchian *Allodaposuchus subjuniperus* from Beranuy near Arén in Huesca (Puértolas-Pascual et al. 2014; Fig. 9A), and the crocodyloid *Arenysuchus gascabadiolorum* from Arén (Puértolas et al. 2011; Fig. 9B). All of these taxa have been identified and diagnosed on the basis of cranial remains, although in the case of Laño these elements are incomplete and disarticulated.

The phylogenetic relationships of these crocodyliforms are the subject of intense debate. For instance, *Acynodon* has usually been regarded as a basal member of Globidonta within Alligatoroidea (Buscalioni et al. 1999; Martin 2007; Delfino et al. 2008b). However, an alternative phylogenetic hypothesis has placed this brevirostrine eusuchian within Hylaeochampsidae (Brochu et al. 2012; Puértolas-Pascual et al. 2014). *Musturzabalsuchus* is also the subject of recent debate concerning its relationships with basal alligatoroids and other basal eusuchians like *Allodaposuchus* and *Massaliasuchus* (Martin and Delfino 2010; Narváez and Ortega 2011).

Additional Iberian eusuchian crocodyliforms are represented by more fragmentary fossil material. Cranial and postcranial remains from the lower Maastrichtian of Fumanya in Barcelona Province have been provisionally identified as *Allodaposuchus* sp. (Blanco et al. 2013). Newly discovered material from Lo Hueco, which includes several complete articulated skulls and postcranial bones, suggests the occurrence of two different (probably new) taxa. A preliminary study indicates that they are part of a group of basal eusuchians closely related to *Allodaposuchus* (Narváez et al. 2013; Ortega et al. submitted). Remains from Armuña, Vilamitjana (Lleida), and Laño have been referred to *Allodaposuchus precedens* (Buscalioni et al. 2001), but this material is in need of revision (Delfino et al. 2008a; Narváez and Ortega 2011; Puértolas-Pascual et al. 2014).

Some non-eusuchian crocodyliforms are also known from Iberia. *Doratodon ibericus* is based on a partial jaw with ziphodont dentition from Chera (Company et al. 2005; Fig. 8H). In Central Europe, *Doratodon* is represented by *Doratodon carcharidens* from the lower Campanian of Muthmannsdorf, Austria (see above, section **C**). This enigmatic crocodyliform has been recently interpreted as a member of Ziphosuchia and possibly related to sebecosuchians (Company et al. 2005; see also Rabi and Sebők in press) or to notosuchians (Bronzati et al. 2012). Furthermore, ziphodont teeth from Laño are similar to those of *Ischyrochampsa* (Buscalioni et al. 1999), a taxon first described as trematochampsid but later regarded as an early-diverging member of Neosuchia (Buscalioni et al. 2003).

**Pterosaurs.** Pterosaur remains have been described from a few latest Cretaceous sites of the Iberian Peninsula. Most of these fossils are from Laño and Tous (La Solana and Chera), and have been referred to Azhdarchidae (Buffetaut 1999; Company et al. 1999). The material from Laño consists of a jaw fragment and postcranial bones, including cervical vertebrae and some wing metacarpals and phalanges, belonging to several individuals (Astibia et al. 1990; Buffetaut 1999). In a preliminary study, Buffetaut (1999) referred the material to cf. *Azhdarcho*. The Laño azhdarchid had a minimum wingspan of 3 to 3.5 meters (Buffetaut 1999). Pterosaur remains from La Solana include incomplete cervical vertebrae (some of them of gigantic size) and fragmentary wing bones, which have been referred to an indeterminate azhdarchid (Company et al. 1999; Company 2004; Fig. 9F). In addition, wing phalanges of an indeterminate azhdarchid have been found at the site La Castellana-2 near Chera (Pereda-Suberbiola et al. 2007). Recently, a femur and other fragmentary pterosaur long bones belonging to very large individuals were described from the the Torrebilles-2 site near Isona (Dalla Vecchia et al. 2013). The site occurs within magnetochron C29r in the uppermost Maastrichtian part of the Tremp Formation, and therefore is less than 400,000 years older than the Cretaceous-Palaeogene boundary (Dalla-Vecchia et al. 2013).

In Portugal, material from Viso described by Lapparent and Zbyszewki (1957) is no longer regarded as pterosaurian (Galton 1994).

**Dinosaurs: Overview.** Dinosaurs from the upper Campanian–Maastrichtian of the Iberian Peninsula include a diverse array of titanosaurian sauropods, neoceratosaurian and coelurosaurian theropods (including dromaeosaurids and probably birds), rhabdodontid and hadrosauroid ornithopods, and nodosaurid ankylosaurs. Titanosaurs and hadrosauroids are the most diverse and abundant groups of large-bodied herbivores. Titanosaurian remains are commonly found in sites of late Campanian to early Maastrichtian age (Vila et al. 2012) whereas hadrosauroid remains occur abundantly in upper Maastrichtian outcrops (Cruzado-Caballero 2012; Prieto-Márquez et al. 2013). Contrary to previous assertions, there is no evidence of pachycephalosaurs or ceratopsians in the Portuguese (or broader Iberian) fossil record (Pereda-Suberbiola 1999b; Antunes and Mateus 2003).

**Dinosaurs: Ankylosaurs.** Several fossils from Laño, including cranial and mandibular remains, teeth, and postcranial bones, have been referred to the nodosaurid ankylosaur *Struthiosaurus* sp. (Pereda-Suberbiola 1999a; Garcia and Pereda-Suberbiola 2003; Fig. 8F). *Struthiosaurus* fossils are also known from Chera, including an incomplete skull and postcranial elements (Company et al. 2009c). Nodosaurid teeth have been reported from Quintanilla del Coco in Burgos (Pol et al. 1992). In the south-central Pyrenees of Lleida, only a few sites have yielded ankylosaurian teeth and bones, but the systematic affinities of these specimens are debatable (Riera et al. 2009; Escaso et al. 2010). In Portugal, teeth described as *Taveirosaurus costai* from Taveiro have been interpreted as either those of a juvenile ankylosaur or an indeterminate ornithopod (Antunes and Sigogneau-Russell 1991, 1996; Galton 1996; Pereda-Suberbiola 1999a; Norman et al. 2004). These specimens are not diagnostic and thus *Taveirosaurus costai* should be regarded as a *nomen dubium* (contra Antunes and Mateus 2003).

**Dinosaurs: Ornithopods.** Both rhabdodontid and hadrosauroid ornithopods are found at various sites across Iberia. Hadrosauroid remains are especially abundant in late Maastrichtian aged localities of the south-central Pyrenees (Cruzado-Caballero 2012 and references therein). The only evidence of hadrosauroids in the pre-late Maastrichtian of the Iberian Peninsula is a single tooth from Laño (Pereda-Suberbiola et al. 2003). Consequently, Laño is the only Iberian locality where hadrosauroids and rhabdodontids have been discovered together, somehow reminiscent of the case of the southern French locality of Vitrolles-la-Plaine (Valentin et al. 2012).

Among hadrosauroids, three clearly diagnostic taxa belonging to the major subclade Lambeosaurinae have been named from Iberia, mainly based on cranial remains: *Pararhabdodon isonensis* from Sant Romà d’Abella in Lleida, respectively *Arenysaurus ardevoli* (Fig. 9C–D) and *Blasisaurus canudoi* from Blasi (Pereda-Suberbiola et al. 2009b; Prieto-Márquez et al. 2006, 2013; Cruzado-Caballero et al. 2010a, 2013). *Arenysaurus* and *Blasisaurus* form a clade of derived lambeosaurines that might be closely related to *Parasaurolophus* (Cruzado-Caballero et al. 2013) or *Hypacrosaurus* (Prieto-Márquez et al. 2013). *Pararhabdodon*, on the other hand, appears to be closely related to the basal Asian lambeosaurin *Tsintaosaurus* (Prieto-Márquez and Wagner 2009). Another potential lambeosaurine taxon, ‘*Koutalisaurus kohlerorum*’ from Les Llaus in Lleida, based on an isolated dentary without teeth (Fig. 9E), could be a subjective junior synonym of *Pararhabdodon isonensis* (Pereda-Suberbiola et al. 2009a; Prieto-Márquez and Wagner 2009; discussed as Lambeosaurinae indet. by Prieto-Márquez et al. 2013). Other lambeosaurine specimens are indeterminate at the genus and species level (Cruzado-Caballero 2012; Prieto-Márquez et al. 2013).

Additional hadrosauroid material from Iberia is more fragmentary, meaning that the systematic affinities of specimens are often uncertain. It is currently debatable whether the other major hadrosaurid subclade, Saurolophinae, may be represented by fragmentary material from Arén (Cruzado-Caballero et al. 2010b; Prieto-Márquez et al. 2013). Other mandibular and postcranial material from Lleida and Valencia has been regarded as Euhadrosauria indet., Hadrosauridae indet., or Hadrosauroidea indet. (Pereda-Suberbiola et al. 2009a; Cruzado-Caballero et al. 2014), meaning that it is unclear whether some or all of this material belongs to Hadrosauridae or more inclusive clades. Additionally, footprints (ichnogenus *Hadrosauropodus*) and eggshells (oogenus *Spheroolithus*) from the southern Pyrenees have been referred to hadrosaurids, although these assignments are not unequivocal (Vila et al. 2013a; Sellés et al. 2014b).

Rhabdodontids are less abundant in the Iberian localities than in the late Campanian to early Maastrichtian sites of southern France and the Maastrichtian sites of Romania. Specimens from Laño, Chera, Lo Hueco, and Armuña have been provisionally assigned to *Rhabdodon* sp. (Pereda-Suberbiola and Sanz 1999; Company 2004; Corral Hernández et al. 2007; Escaso et al. 2012; Fig. 8E), but this material is in need of revision.

**Dinosaurs: Non-avian theropods.** Iberian theropods are mainly represented by isolated teeth, but a few bones are also known. A collection of nearly 150 teeth from Laño and a few other localities from the south-central Pyrenees (Blasi; Vicari-4, Montrebei, Figuerola-2, and Fontllonga-6 in Lleida) constitutes one of the richest samples of non-avian theropods in the Late Cretaceous of Europe (Torices et al. in press). At least five taxa of small theropods and one large theropod are present in this assemblage, including *Paronychodon*, *Richardoestesia*, and two morphotypes of dromaeosaurids (Torices et al. 2004, in press). Another taxon based on teeth, *Euronychodon*, which was first described from Taveiro (Antunes and Sigogneau-Russell 1991), is now regarded as a subjective junior synonym of *Paronychodon* (Rauhut 2002; Sues and Averianov 2013; Torices et al. in press). Some of the very rare non-dental remains of theropods from Iberia include a set of long bones from Laño that may belong to an abelisaurid closely related to *Tarascosaurus* (Le Loeuff and Buffetaut 1991). The Iberian record of theropod eggs consists mainly of oospecies of Prismatoolithidae, Elongatoolithidae, and Laevisoolithidae (Sellés et al. 2014a and references).

**Dinosaurs: Birds.** A few bones from Laño exhibit bird-like features (Buffetaut et al. 2006). One of these, a partial sacrum initially considered possibly pterosaurian, belongs to a large ground bird probably closely related to *Gargantuavis* (Buffetaut et al. in prep.). The presence of birds is also suggested by the ootaxon *Sankofa pyrenaica* from the Montsec area of Lleida (López-Martínez and Vicens 2013).

**Dinosaurs: Sauropods.** Titanosaurian fossils have been found at over a dozen Campanian to Maastrichtian localities on the Iberian Peninsula (Díez Díaz 2013; Vila et al. 2012). Much of this material is fragmentary and indeterminate at the genus and species level (Royo Torres 2009; Díez Díaz 2013). A collection of bones from Laño, including a basicranium, teeth, vertebrae, appendicular elements, and osteoderms, was described as a new genus and species, *Lirainosaurus astibiae*, which is regarded as a derived lithostrotian close to Saltasaurinae (Sanz et al. 1999; Díez Díaz et al. 2011, 2012, 2013a,b,c). *Lirainosaurus* is also represented by referred postcranial specimens from Chera (Company et al. 2009a). It is a slender, small-bodied titanosaur; the largest individuals probably did not exceed 6 meters in length and 2–4 tons in weight (Díez Díaz et al. 2013b).

Recently discovered titanosaurian remains from Lo Hueco include numerous isolated bones belonging to partial skeletons of several individuals (Ortega 2013). At least two different titanosaurs appear to be present at Lo Hueco: a robust taxon exhibiting affinities with the “Massecaps titanosaur” from Cruzy, Languedoc, and a slender form that is highly autapomorphic (Díez Díaz 2013). This material remains to be named and fully described. Other noteworthy titanosaurian specimens from Spain include a well-preserved braincase from Lo Hueco provisionally referred to *Ampelosaurus* sp. (Knoll et al. 2013; Fig. 8G), an abundance of megaloolithid eggs and eggshells from a number of localities in Lleida and Barcelona that were probably laid by titanosaurs (Vila et al. 2011, 2012), and wide-gauge trackways from Fumanya (Barcelona) that were attributed to titanosaur trackmakers (Vila et al. 2013b).

**Mammals.** Only tribosphenic therian mammals have been described from the Upper Cretaceous of the Iberian Peninsula. The fauna is dominated by eutherians, and especially zhelestids, including *Lainodon orueetxebarriai* (Fig. 8J) and *Lainodon ragei* from Laño (Gheerbrant and Astibia 1994, 1999, 2012), and *Labes quintanillensis* from Quintanilla del Coco in Burgos (Pol et al. 1992). *Lainodon* is also present at Taveiro (Antunes et al. 1986; Gheerbrant and Astibia 2012). The zhelestids from the Iberian Peninsula and southern France belong to the clade Lainodontinae, which is the dominant mammalian group in the Late Cretaceous assemblages of southwestern Europe (Gheerbrant and Astibia 2012). A tooth from Quintanilla del Coco may belong to the “cimolestan” group Palaeoryctidae (Pol et al. 1992), which may or may not be placental mammals. Multituberculates appear to be absent in the Iberian localities despite their abundance elsewhere in Europe (e.g., Romania; see below, section **F**), although they are recorded from the lower Paleogene (Danian) of northern Spain (Peláez-Campomanes et al. 2000).

### F. Coniacian and Campanian–Maastrichtian, Romania

#### History of research

The first report of Late Cretaceous continental vertebrates from the Transylvanian region was made by Halaváts (1897), a geologist of the Royal Hungarian Geological Survey who was mapping in the western Southern Carpathians, including the Haţeg Basin. He considered these vertebrate fossils as “Aquitanian” (early Miocene) in his views on the local stratigraphy. A short time later, Nopcsa (1897, 1899) initiated large-scale collecting and detailed study of the Haţeg vertebrates. He also re-dated them as “Danian” (i.e., latest Cretaceous) and recognized their wider distribution across Transylvania (Nopcsa 1905). Based on his studies on these specimens, Nopcsa established himself as a leading figure in European vertebrate paleontology. Over the next few decades he described a rich fossil assemblage from Transylvania (Figs 3, 4), including turtles, crocodyliforms, pterosaurs, and many types of dinosaurs (e.g., Nopcsa 1900, 1902a, 1904, 1915, 1923b, 1928, 1929; Andrews 1913; Huene 1932). Based on its high degree of endemicity, primitiveness, and inferred small body size of many taxa, Nopcsa (1914, 1923a, 1934) interpreted this peculiar assemblage as an insular fauna, similar in many respects to the (then recently discovered) Plio–Pleistocene faunas of the Mediterranean.

**Figure 10.**
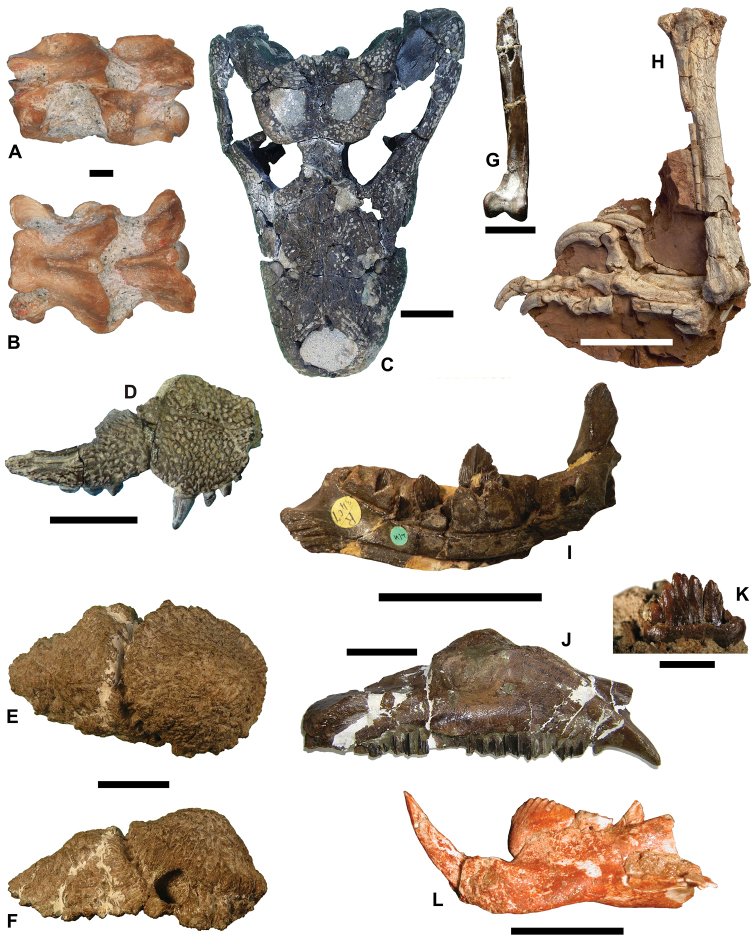
Representative taxa from the latest Campanian–Maastrichtian faunas from Transylvania, western Romania. **A–B** *Nidophis insularis* (Serpentes, Madtsoiidae), articulated vertebrae (LPB (FGGUB) v.547/2) in left lateral (**A**) and dorsal (**B**) views (Densuş-Ciula Formation, Tuştea, Haţeg Basin; photo by Ştefan Vasile) **C** *Allodaposuchus precedens* (Eusuchia, ?Hylaeochampsidae), skull (PSMUBB V 438) in dorsal view (Sebeş = Şard Formation, Oarda de Jos, southwestern Transylvanian Basin; photo by Vlad Codrea/Massimo Delfino) **D** *Theriosuchus sympiestodon* (Mesoeucrocodylia, Atoposauridae), right maxilla (MCDRD 793) in lateral view (Sînpetru Formation, Sînpetru, Haţeg Basin) **E–F** Indeterminate titanosaur (?*Magyarosaurus dacus*) (Sauropoda, Titanosauria), isolated osteoderm (LPB (FGGUB) R.1410) in dorsal (**E**) and lateral (**F**) views (Sînpetru Formation, Sînpetru, Haţeg Basin) **G** Indeterminate ornithuran bird (Aves, Ornithurae), incomplete left tibiotarsus (LPB (FGGUB) R.1902) in anterior view (Densuş-Ciula Formation, Vălioara, Haţeg Basin) **H** *Balaur bondoc* (Theropoda, Dromaeosauridae), articulated left distal hindlimb (EME PV.313) in lateral view (Sebeş = Şard Formation, Sebeş-Glod, southewestern Transylvanian Basin; photo by Mick Ellison) **I** *Zalmoxes robustus* (Ornithopoda, Rhabdodontidae), right dentary (NHMUK R.3407) in medial view (Sînpetru Formation, Sînpetru, Haţeg Basin) **J** *Telmatosaurus transsylvanicus* (Hadrosauria), right maxilla (MFGI unnumbered) in lateral view (Sînpetru Formation, Sînpetru, Haţeg Basin) **K** Indeterminate nodosaurid – *Struthiosaurus transylvanicus* or new taxon – (Ankylosauria, Nodosauridae), isolated tooth (LPB (FGGUB) R.2182) in medial view (Sînpetru Formation, Sînpetru, Haţeg Basin) **L** *Barbatodon transylvanicus* (Multituberculata, Kogaionidae), right maxilla (LPB (FGGUB) M.1635) in medial view (Sînpetru Formation, Pui, Haţeg Basin). Scale bars equal 1 mm in **A, B**; 5 mm in **K**; 1 cm in **G, L**; 2 cm in **D**; and 5 cm in **C, E, F, H, I, J.**

More recently, renewed collecting in the uppermost Cretaceous continental deposits of Transylvania has led to a better understanding of the peculiar Romanian vertebrate assemblage (Grigorescu 2005, 2010a; Weishampel and Jianu 2011). The faunal list was extended to include many new species of fishes, amphibians (anurans and albanerpetontids), squamates (lizards and snakes), turtles, diverse crocodyliforms, pterosaurs, and dinosaurs (ornithopods, nodosaurids, sauropods, non-avian and avian theropods), and multituberculate mammals (see Grigorescu 2005; Therrien 2005; Benton et al. 2010; Weishampel et al. 2010; Fig. 10). Moreover, the distribution of the Maastrichtian vertebrate assemblage was extended significantly outside the Haţeg Basin by discoveries in the adjoining intermontane Transylvanian (Codrea and Godefroit 2008; Codrea et al. 2010a,b,c; Vremir 2010) and Rusca Montană Basins (Vasile and Csiki 2011; Codrea et al. 2012). During the same period, intense multidisciplinary research targeting the fossil-bearing deposits expanded knowledge on their geological setting, stratigraphy, age, and paleoenvironments (Grigorescu 2010a).

#### Geological setting

The oldest Late Cretaceous terrestrial vertebrates from Transylvania are fragmentary specimens from the Coniacian to possibly lower Santonian continental deposits of the Borod Basin in the northern part of the Apuseni Mountains (Figs 1, 2). These include the isolated teeth of a small theropod described by Nopcsa (1902b) as *Megalosaurus hungaricus*, today recognized as an indeterminate taxon possibly related to either tyrannosauroids or dromaeosaurids (Holtz et al. 2004b; Carrano et al. 2012). The Borod site is slightly older than the oldest well-known European Late Cretaceous vertebrate assemblage, Iharkút in Hungary. Although the Borod sample is extremely poor, it indicates that colonization of emergent islands in Transylvania occurred as early as the Coniacian–?early Santonian.

The more extensive and better sampled uppermost Cretaceous units of Transylvania are spread across a large area, extending approximately 300 kilometers southwards from near the town of Jibou in northern Romania (Nopcsa 1905; Grigorescu 1992; Codrea et al. 2010c, 2012; Figs 1, 3, 4). These deposits outcrop discontinuously, mainly along the western-southwestern margin of the Transylvanian Basin, as well as in smaller adjoining intermontane basins (Haţeg, Rusca Montană) in the Southern Carpathians. Coeval volcanoclastic deposits also occur in several intermontane basins within the Apuseni Mountains, but have yet to yield vertebrate fossils. Despite their geographic extent, the continental deposits are remarkably uniform lithologically across Transylvania. They are comprised of alluvial (channel and floodplain) units accumulated in post-orogenic collapse basins at the foothills of actively eroding mountains (Ciulavu 1999; Willingshofer et al. 2001; Krézsek and Bally 2006). These continental sequences conformably or unconformably overlie transitional, paralic-deltaic successions that document the final phases of marine transgression before land areas emerged towards the end of the Cretaceous (Codrea and Dica 2005; Codrea et al. 2010c; Vremir 2010; Vremir et al. 2014).

Most of the uppermost Cretaceous terrestrial succession consists of channel sandstones and conglomerates, interbedded with moderately to well-drained floodplain deposits, which formed in low-sinuosity fluvial systems under a seasonally variable and semiarid climate (Codrea et al. 2001, 2010c; Van Itterbeeck et al. 2004; Bojar et al. 2005; Codrea and Dica 2005; Therrien 2005, 2006; Vremir 2010; Mariş 2012). These indicate that braided rivers surrounded by extensive and relatively dry floodplains were the dominant landscape in the region during the latest Cretaceous. Some facies variations, however, do occur. In certain areas of the Rusca Montană and northwestern Haţeg basins, volcanoclastic deposits (tuffs, tuffites, volcanic agglomerates) and even volcanic rocks (lava flows), together with coal-bearing clastics, are the dominant lithology (Dincă 1977; Petrescu and Duşa 1982; Anastasiu and Csobuka 1989; Grigorescu 1992; Bârzoi and Şeclăman 2010). In some areas of the Haţeg Basin, dark grey calcic vertisols and coarser-grained channel and crevasse sandstones indicate meandering river systems with localized freshwater ponds and wetlands (Van Itterbeeck et al. 2004; Therrien 2006; Săsăran et al. 2011). Similar lacustrine deposits are also recorded in the southwestern Transylvanian Basin (Codrea et al. 2001, 2010c; Vremir 2010). Finally, along the marginal areas of the Transylvanian Basin at Stăuini and Petreşti, there are transitional estuarine-deltaic deposits at the base of the continental succession that grade into the more characteristic fluvial and floodplain lithologies (Therrien 2005; Codrea et al. 2010c; Vremir 2010; Vremir et al. 2014).

All of these sedimentary sequences accumulated on continental fragments that initially detached through mid-Mesozoic crustal stretching, and subsequently assembled into larger blocks during mid-to-Late Cretaceous collisions (e.g., Csontos and Vörös 2004; Willingshofer 2000). The emerged areas of Transylvania were located on a composite block (Tisia-Dacia terrane) that formed a subtropical island placed at about 28° northern latitude (Panaiotu and Panaiotu 2010). This island, often referred to as the “Haţeg Island” in the literature, was part of a larger archipelago fringing the northern margin of the Mediterranean Tethys (Benton et al. 2010). Living on this island was the characteristic dwarfed, primitive, and aberrant Transylvanian island fauna that is recognized as one of the most unusual Mesozoic terrestrial assemblages in the global fossil record (Nopcsa 1914; Weishampel et al. 1991; Benton et al. 2010; Weishampel and Jianu 2011).

The age of the uppermost Cretaceous continental deposits of Transylvania is poorly constrained. Similarities in the composition of fossil floras and faunas between various sites suggest that the fossil-bearing units were deposited roughly synchronously across their outcropping area (Nopcsa 1905; Grigorescu 1992; Codrea and Godefroit 2008; Codrea et al. 2010c, 2012; Vasile and Csiki 2011; Vasile and Csiki-Sava 2012). Diverse palynostratigraphic (Antonescu et al. 1983; Van Itterbeeck et al. 2005), paleobotanical (Petrescu and Duşa 1982), and magnetostratigraphic (Panaiotu and Panaiotu 2010; Panaiotu et al. 2011) data, mainly from the Haţeg Basin, support a Maastrichtian age for the fossil-bearing units. This age assessment was recently corroborated by early Maastrichtian (69.8±1.3 Ma and 71.3±1.6 Ma) radiometric dates reported from tuff beds interspersed within the continental deposits from the northwestern Haţeg Basin (Bojar et al. 2011). The base of the Transylvanian fossiliferous continental succession, however, appears to be latest Campanian in age based on microfossil biostratigraphy (Vremir et al. 2014). This indicates that coastal, and even purely continental, environments populated by a terrestrial fauna were probably already forming locally by the latest Campanian (Vremir et al. 2014).

#### Faunal overview

**Fishes.** Fishes are surprisingly rare in the uppermost Cretaceous of Transylvania. Grigorescu et al. (1985) first reported indeterminate acipenseriform and characid fossils from Pui. Subsequently, the occurrence of lepisosteids (both *Lepisosteus* and *Atractosteus*) and indeterminate teleosteans in a few microfossil bonebeds from the Haţeg Basin was noted by Grigorescu et al. (1999), Csiki et al. (2008), and Vasile and Csiki (2010). A substantially richer fish assemblage, including both lepisosteids (*Lepisosteus*) and characids, is now known from lacustrine deposits of the Transylvanian Basin (Codrea and Jipa, personal communication, 2011). The scarcity of fish remains does appear to be genuine in most studied deposits, as taphonomic or preservational factors do not easily explain the presence of a large number of fragile but well-preserved frog remains alongside the much rarer, but otherwise resistant ganoid scales.

**Amphibians.** Anuran and albanerpetontid amphibians are relatively common fossils in the Transylvanian deposits. Anuran remains are common, and sometimes greatly abundant, in most microvertebrate bonebeds in the uppermost Cretaceous succession (e.g., Grigorescu et al. 1999; Codrea et al. 2002; Smith et al. 2002; Folie and Codrea 2005; Vasile and Csiki 2010, 2011). Most diagnostic frog specimens can be referred to Discoglossidae, including *Paralatonia transylvanica*, associated with another, less common *incertae sedis* taxon described as *Hatzegobatrachus grigorescui* (Venczel and Csiki 2003). Some anuran genera previously known from other continental landmasses and much older time periods have been reported from the Haţeg Basin (Grigorescu et al. 1999; Folie and Codrea 2005), but such accounts should be viewed with caution until they are better substantiated. These problematic taxa include *Paradiscoglossus* (known from the Maastrichtian of North America; Estes and Sanchíz 1982a) and *Eodiscoglossus* (known from the Middle Jurassic to Lower Cretaceous of Europe; Estes and Sanchíz 1982b; Evans et al. 1990).

Albanerpetontid fossils are almost as numerous as those of the anurans, but appear to belong exclusively to the genus *Albanerpeton* (Grigorescu et al. 1999; Codrea et al. 2002; Folie and Codrea 2005; Codrea et al. 2010a; Vasile and Csiki 2011), a geographically widespread mid-Cretaceous to early Pleistocene taxon (Gardner and Böhme 2008). The more precise species-level affinities of the Transylvanian albanerpetontids are currently under study, but they appear to be closely related to the derived clade of ’robust-snouted’ species of *Albanerpeton* (Folie and Codrea 2005; Venczel et al. 2013; Z.Cs.-S., personal observation). The Late Jurassic to Early Cretaceous genus *Celtedens* was also reported as present in the Transylvanian uppermost Cretaceous by Grigorescu et al. (1999), but the referred element most likely is an incomplete frontal of *Albanerpeton*.

**Turtles.** Turtle remains, especially carapace fragments, are among the most common fossils in the Maastrichtian beds of Transylvania. Until recently, the basal turtle *Kallokibotion bajazidi* (Nopcsa 1923a, b) was the only chelonian taxon reported from the area, first from the Haţeg Basin and then subsequently from the Transylvanian (Codrea and Vremir 1997; Vremir 2004) and Rusca Montană (Codrea et al. 2012) basins. *Kallokibotion* was considered a basal cryptodiran turtle (e.g., Gaffney and Meylan 1992), but there is mounting evidence that this taxon (and related forms from central-eastern Europe; Rabi et al. 2013a) is actually a member of the Meiolaniformes, a basal non-pantestudine clade with a mainly southern Gondwanan distribution (e.g., Sterli and de la Fuente 2013; Sterli et al. in press).

More recently, it has been recognized that the Transylvanian Maastrichtian turtle assemblages were more diverse than previously thought (Vremir 2004). Dortokid turtles, an endemic European clade, have now been identified in the Transylvanian Basin (‘*Muehlbachia nopcsai*’; Vremir and Codrea 2009) and the Haţeg Basin (Rabi et al. 2013a). Among the dortokids, the latest Cretaceous Transylvanian taxa appear more closely related to the Campanian–Maastrichtian Ibero-Armorican *Dortoka* (Lapparent de Broin and Murelaga 1999) than to the latest Paleocene Transylvanian *Ronella* (Lapparent de Broin et al. 2004). Vremir (2004) also noted the occurrence of possible bothremydid turtles (?*Polysternon*) in Transylvania, but without providing further details or description. The presence of bothremydids in Romania was rejected, however, by Lapparent de Broin et al. (2009), and is not mentioned in the most recent review of the Maastrichtian turtles from Transylvania by Rabi et al. (2013a).

**Squamates.** Squamates represent a common and taxonomically diverse component of the Transylvanian vertebrate assemblages. Lizards in particular are rather abundant, whereas snakes are much less common.

Lizards were first recognized in the Romanian uppermost Cretaceous by Grigorescu et al. (1985), and subsequently have been found in most microvertebrate bonebeds from the Haţeg and Transylvanian basins (Grigorescu et al. 1999; Codrea et al. 2002, 2010a; Folie and Codrea 2005; Csiki et al. 2008). In addition, eggshell fragments (probably belonging to squamates) are also common at these sites. Nonetheless, most known lizard remains are fragmentary and of little diagnostic value. Folie and Codrea (2005) assigned material to several taxa, including a polyglyphanodontin boreioteiid (*Bicuspidon hatzegiensis*, considered closely related to the mid-Cretaceous North American *Bicuspidon numerosus*; Nydam and Cifelli 2002) and the paramacellodids *Becklesius nopcsai* and *Becklesius* cf. *Becklesius hoffstetteri*. If correctly assigned, these paramacellodid taxa would represent survivors of a Late Jurassic to Early Cretaceous lineage. Furthermore, Csiki et al. (2008) mentioned the presence of ?*Slavoia*, whereas Weishampel et al. (2010) listed, but without supporting evidence, the possible presence of the contogeniid ?*Contogenys* (Nydam and Fitzpatrick 2009) and the xantusiid ?*Paracontogenys*. All other lizard remains from Transylvania are currently indeterminate at lower taxonomic levels, and are usually referred to as Scincomorpha indet. or (more rarely) as Anguimorpha indet. Future work may be able to more precisely identify some of the better-preserved specimens.

Snakes are the most recent major addition to the faunal list of the Transylvanian Maastrichtian. An isolated vertebra from Pui was referred to Madtsoiidae (Folie and Codrea 2005), a taxon with an almost exclusively Gondwanan distribution during the Cretaceous (Rage 1999; LaDuke et al. 2010). Subsequently, more complete and better-preserved fossils from the northwestern Hațeg Basin were described as a new madtsoiid snake, *Nidophis insularis* (Vasile et al. 2013; Fig. 10A–B). For the moment, snake fossils remain restricted to the Haţeg Basin, although this is probably due to sampling biases caused by their low abundance.

**Crocodyliforms.** Crocodyliform remains are among the most frequently encountered vertebrate fossils in the Maastrichian of Transylvania, particularly as isolated teeth. The first crocodyliform described was *Allodaposuchus precedens* (Nopcsa 1928; formerly mentioned as *Crocodylus affuvelensis* by Nopcsa 1915), based on a fragmentary skull and postcranial remains. For decades, *Allodaposuchus* was regarded as a basal eusuchian (e.g., Buscalioni et al. 2001) or even as a basal alligatoroid (Martin 2010a). Discovery of better preserved, complete skull remains in Romania (Fig. 10C) and elsewhere (Delfino et al. 2008a; Puértolas-Pascual et al. 2014) and more detailed phylogenetic analyses (Brochu et al. 2012; Puértolas-Pascual et al. 2014) suggest instead that it might represent a basal eusuchian closely related to Hylaeochampsidae, an almost exclusively European endemic clade (e.g., Buscalioni et al. 2011).

As in the case of the turtles, recent collecting has unearthed a much higher diversity of crocodyliforms in the Transylvanian uppermost Cretaceous than previously thought (Martin et al. 2006). *Doratodon*, a ziphodont crocodyliform originally described from the lower Campanian of Austria (Bunzel 1871; see section **C**), is known from isolated teeth that possess the characteristic serrated, triangular, and laterally compressed morphology of the genus. The presence of *Acynodon*, Ibero-Armorican taxon first described by Buscalioni et al. (1997, 1999) was similarly recognized based on isolated, typically spatulate-shaped teeth. Although generally considered a peculiar heterodont alligatoroid (Buscalioni et al. 1999; Martin 2007; Puértolas et al. 2011), *Acynodon* has also been recovered as a basal eusuchian closely related to Hylaeochampsidae in recent phylogenetic analyses (Brochu et al. 2012). Finally, more complete specimens have been assigned to a Transylvanian species of the atoposaurid *Theriosuchus*, *Theriosuchus sympiestodon* (Martin et al. 2010, 2014; Fig. 10D). This heterodont taxon is characterized by a fang-like fourth maxillary tooth followed immediately by low-crowned, laterally compressed, and pseudoziphodont leaf-shaped posterior teeth. Remains of *Theriosuchus* and *Acynodon* are currently restricted to the Haţeg Basin, whereas teeth of *Doratodon* have also been described from the Rusca Montană Basin by Vasile and Csiki (2011).

**Pterosaurs.** Early reports of pterosaurs in the Haţeg Basin were problematic. Nopcsa (1923a) mentioned a handful of specimens referred to the pterosaur ‘*Ornithodesmus*’. One of these fossils, an isolated sacrum, was recently reinterpreted as belonging to a maniraptoran dinosaur (Ősi and Főzy 2007), and all other material appears to be lost. Jianu et al. (personal communication, 1997) identified new pterosaur remains from the Haţeg Basin, but this material, considered to represent a small-sized pteranodontid, was never fully described and is not currently available for study. Only recently were the first diagnostic pterosaur remains reported from the Haţeg Basin, referred to a new gigantic azhdarchid, *Hatzegopteryx thambema* (Buffetaut et al. 2002a). This colossal taxon, which is likely one of the largest known flying organisms of all time (Witton and Habib 2010), is characterized by a large and robust skull and appendicular bones with a highly pneumatic, sponge-like internal structure. A second, medium-sized azhdarchid, *Eurazhdarcho langendorfensis*, was described from the Transylvanian Basin by Vremir et al. (2013), suggesting a higher local taxonomic and paleoecological diversity of pterosaurs than once surmised; however, the taxonomic distinctiveness of the latter taxon was questioned by Averianov (2014).

**Dinosaurs: Ankylosaurs.** Despite the early discovery of the first specimens (Nopcsa 1915), ankylosaur remains continue to be comparatively rare in the Transylvanian Maastrichtian. Nopcsa (1915, 1929) named associated fossils (braincase, vertebrae, and elements of the shoulder girdle) of a small-sized nodosaurid from the Haţeg Basin as *Struthiosaurus transylvanicus*, considering it closely related to *Struthiosaurus austriacus* from Muthmannsdorf (Bunzel 1871; Seeley 1881). The taxonomic identity of this material remained ambiguous for many decades, as it was sometimes considered synonymous with *Struthiosaurus austriacus* (e.g., Pereda-Suberbiola and Galton 1994, 1997), or even interpreted as representing a distinct genus (e.g., Coombs and Maryanska 1990). More recent reviews accept *Struthiosaurus transylvanicus* as a valid taxon closely related to the Austrian species (e.g., Vickaryous et al. 2004). *Struthiosaurus* is usually recovered as a relatively basal nodosaurid in phylogenetic analyses (e.g., Ősi and Makádi 2009; Thompson et al. 2012).

In recent years, new nodosaurid discoveries have been reported, although they remain uncommon compared to those of other dinosaurs. Such discoveries are more numerous in the Transylvanian Basin (e.g., Codrea et al. 2010b,c; Vremir 2010), but are restricted to a few isolated teeth in the Haţeg Basin (Codrea et al. 2002). Nodosaurids are as yet unknown from the Rusca Montană Basin. This new material has usually been referred to *Struthiosaurus transylvanicus*, but recent review of the specimens has failed to identify evidence for its referral to *Struthiosaurus*, with the exception of a diagnostic humerus and elements associated with it (Ősi et al. 2014a). Furthermore, a distinctive nodosaurid tooth suggests either the presence of a second nodosaurid taxon or of a previously unreported tooth morphotype in *Struthiosaurus* (Ősi et al. 2014a; Fig. 10K). Clearly, a specimen-level revision of the Transylvanian nodosaurids is needed.

The small size of the Transylvanian nodosaurids might be evidence for their dwarf status (see Nopcsa 1915). However, Pereda-Suberbiola and Galton (2009) argued against this hypothesis, instead considering small size to represent an ancestral character state retained from primitive ancestors that did not live on islands and were not heterochronic dwarfs.

**Dinosaurs: Ornithopods.** The Late Cretaceous ornithopod assemblage from Transylvania is locally abundant and taxonomically diverse, with different taxa often co-occuring at the same sites. This is unlike the case in other roughly contemporaneous European assemblages where either basal euornithopods or hadrosauroids dominate (e.g., Le Loeuff et al. 1994; Vila et al. 2013a). Ornithopods were among the first Transylvanian dinosaurs discovered and described by Nopcsa, who named the hadrosauroid ’*Limnosaurus*’ (=*Telmatosaurus*) *transsylvanicus* (Nopcsa 1900; see also Weishampel et al. 1993) and the basal euornithopod ’*Mochlodon*’ *robustus* (Nopcsa 1902a, 1904) from the Haţeg Basin. The Transylvanian euronithopod material was later referred to its own genus, *Zalmoxes*, by Weishampel et al. (2003), who recognized two species: Nopcsa’s *Zalmoxes robustus* and a second Transylvanian species, *Zalmoxes shqiperorum*.

*Zalmoxes* is a member of the endemic European clade Rhabdodontidae (Weishampel et al. 2003; see also Ősi et al. 2012a), and is characterized by relatively small (2.5–3.5 meters long) size but a robust build. Its remains (Fig. 10I) are among the most commonly encountered vertebrate fossils in the Transylvanian Maastrichtian, being found in the Transylvanian, Haţeg, and Rusca Montană basins (Nopcsa 1905; Weishampel et al. 2003; Codrea and Godefroit 2008; Codrea et al. 2012). It is also one of the most completely known dinosaurs from Transylvania, especially after the description of the first reasonably complete skeleton of *Zalmoxes shqiperorum* from Nălaţ-Vad by Godefroit et al. (2009). Footprints possibly referable to *Zalmoxes* were reported by Vremir and Codrea (2002) from Oarda (Transylvanian Basin).

The hadrosauroid *Telmatosaurus* appears to be less common than *Zalmoxes*, and it is also somewhat larger in size (Fig. 10J). It has only been reported from the Haţeg (Nopcsa 1900; Weishampel et al. 1993) and the southwestern Transylvanian (Codrea et al. 2010b,c; Vremir 2010) basins. Neonate bones, eggs, and nesting sites in Transylvania have also been attributed to *Telmatosaurus* (Grigorescu 2010b; Grigorescu et al. 1990, 1994, 2010). As a result, its growth, reproductive dynamics and early ontogeny are better documented than in any other Transylvanian vertebrates (Grigorescu and Csiki 2006). Phylogenetic analyses usually recover *Telmatosaurus* as a hadrosauroid close to or at the base of the major hadrosaurid radiation (e.g., Weishampel et al. 1993; Prieto-Márquez 2010a).

Nopcsa originally proposed that both Transylvanian ornithopods were smaller than their close relatives and mainland contemporaries, probably a result of their insular island environment (“phyletic dwarfism”). Subsequent authors have largely agreed that both taxa may be examples of island dwarves, based on osteohistological studies (e.g., Benton et al. 2010) and by mapping body-size distribution onto phylogenies (e.g., Weishampel et al. 1993, 2003). However, a more recent and comprehensive phylogenetic analysis of Rhabdodontidae conducted by Ősi et al. (2012a) appears to weaken support for phyletic dwarfism in the case of *Zalmoxes*, and instead suggests that small body size may be a retained primitive feature. It is clear, therefore, that reliable identification of phyletic dwarfism in the two Transylvanian ornithopod genera depends on the exact patterns of basal euornithopod and hadrosauroid phylogeny. Larger and more comprehensive phylogenetic analyses can continue to help test the dwarfism hypothesis.

**Dinosaurs: Non-avian theropods.** Non-avian theropods from Transylvania have remained elusive for many decades. Nopcsa (1915) reported the supposed presence of a large-bodied theropod, *Megalosaurus* sp., but these specimens instead belonged to titanosaurian sauropods (Csiki and Grigorescu 1998). No other non-avian theropod remains were reported until the 1980s, when Grigorescu (1984) and Grigorescu et al. (1985) mentioned the presence of isolated blade-like and serrated teeth with definitive theropod affinities. About the same time, isolated limb bones referred previously to birds, first as the pelecaniform *Elopteryx nopcsai* (Andrews 1913) and then as the giant owls *Bradycneme draculae* and *Heptasteornis andrewsi* (Harrison and Walker 1975), were reinterpreted as small non-avian theropods (Csiki and Grigorescu 1998; Csiki et al. 2010b). The more precise affinities of these specimens, however, are uncertain. They have been assigned by different authors to indeterminate non-maniraptoran tetanurans (Csiki and Grigorescu 1998), dromaeosaurids (e.g., Le Loeuff et al. 1992), troodontids (e.g., Makovicky and Norell 2004), and even alvarezsaurids (Naish and Dyke 2004; Kessler et al. 2005). A consensus has yet to be reached.

Additional non-avian theropod specimens discovered over the past two decades (mainly isolated teeth) have been assigned to a variety of theropods, including an indeterminate medium-sized taxon (Smith et al. 2002), velociraptorine dromaeosaurids (Weishampel and Jianu 1996; Csiki and Grigorescu 1998; Grigorescu et al. 1999; Codrea et al. 2002; Vasile 2008; Codrea et al. 2010a, 2012), and troodontids (Codrea et al. 2002, 2012; Smith et al. 2002). Other specimens have been referred to the tooth form genera *Richardoestesia* (Codrea et al. 2002; Vasile 2008; Vasile and Csiki 2011; Vasile et al. 2012), *Euronychodon* (Csiki and Grigorescu 1998; Codrea et al. 2002; Csiki et al. 2008; Vasile 2008), and *Paronychodon* (Codrea et al. 2002; Vasile and Csiki 2011; Vasile et al. 2012), some or all of which probably represent derived paravian theropods. According to the distribution of such specimens, dromaeosaurids appear to be the most wide-ranging theropods in the Transylvanian Maastrichtian, as their teeth are found at numerous sites in the Haţeg, Rusca Montană, and southwestern Transylvanian basins. Troodontids, *Paronychodon*, and *Richardoestesia* have been reported from the Haţeg and Rusca Montană basins, whereas the possible medium-sized theropod and *Euronychodon* are restricted for the moment to the Haţeg Basin.

The mostly dental record of Transylvanian non-avian theropods was improved dramatically with the description of partial articulated skeleton of the dromaeosaurid *Balaur bondoc* (Csiki et al. 2010b; Brusatte et al. 2013a). This stocky, small-bodied dromaeosaurid with two sickle-like claws on each foot (Fig. 10H) was discovered near the town of Sebeş in the Transylvanian Basin by Mátyás Vremir in 2010. The holotype is the most complete specimen of a Late Cretaceous non-avian theropod from Europe. Isolated appendicular elements previously considered to represent an elmisaurid oviraptorosaur (Csiki and Grigorescu 2005) indicate that *Balaur* (possibly a second species of this genus) was also present in the Haţeg Basin (Brusatte et al. 2013a). Despite its unique anatomical features, most probably related to its insular habitat, the derived position of *Balaur* within the Asian-North American clade of velociraptorines suggests faunal interactions between Europe and these continents well into the Late Cretaceous.

**Dinosaurs: Birds.** Despite early reports of bird remains from the Haţeg Basin, most of these were subsequently reinterpreted as non-avian theropods (see above), thus removing birds from the faunal list of the Transylvanian vertebrate assemblages. It is only very recently that the first unequivocal bird fossils were described, which include representatives of both Ornithurae (Wang et al. 2011a; Fig. 10G) and Enantiornithes (Wang et al. 2011b). The description of new enantiornithine remains from the southwestern Transylvanian Basin (Dyke et al. 2012) further supported the presence of this group in Transylvania, while also suggesting a higher diversity of the clade. These new remains comprise an association of adult and neonatal enantiornithine bones with large quantities of avian eggshell fragments, in what was interpreted as a mass drowning of a nesting-breeding colony.

**Dinosaurs: Sauropods.** Besides turtles, crocodyliforms, and rhabdodontids, the remains of sauropods are among the most common vertebrate fossils throughout the Transylvanian area. They have been recovered from all major fossiliferous units except those of the northwestern Transylvanian Basin (Nopcsa 1915; Codrea et al. 2010c, 2012; Vremir 2010). Without exception, all identifiable specimens can be referred to Titanosauria.

Nopcsa named the taxon ’*Titanosaurus*’ *dacus* based on un-associated skeletal remains from the Haţeg Basin (Nopcsa 1915), and more abundant material from the same area was used by Huene (1932) to erect a new genus (*Magyarosaurus*) with three species (*Magyarosaurus dacus*, *Magyarosaurus transylvanicus* and *Magyarosaurus hungaricus*). Subsequently, the presence of more than one species of *Magyarosaurus* was either accepted (McIntosh 1990) or rejected (Le Loeuff 1993; Upchurch et al. 2004). In most cases, newly discovered titanosaur material was customarily referred to *Magyarosaurus dacus* (Fig. 10D–E), without strong supporting arguments. Although a thorough revision of the Transylvanian sauropod material is still pending, preliminary data suggest the presence of more than one taxon in the area, including one of fairly large size (Z.Cs.-S., personal observation). A higher taxonomic diversity is also supported by the description of a new lithostrotian titanosaur taxon, *Paludititan nalatzensis*, from the Râul Mare succession by Csiki et al. (2010a). The taxonomic identity of the sauropod material from outside the Haţeg area is still largely uncertain, although it appears to include one taxon different from *Magyarosaurus* (Csiki and Vremir 2011).

Recently, dinosaur eggs discovered in the Râul Mare area (Codrea et al. 2002) were referred to titanosaurs by Grellet-Tinner et al. (2012) based on overall egg morphology and eggshell microstructure. This suggests that at least some of the common Haţeg Basin megaloolithid eggs were laid by lithostrotians (possibly by *Paludititan*, which was described from the same succession).

Similar to the case of ornithopods, the small size and seemingly pedomorphic skeletal features of at least some of the Transylvanian titanosaurs (particularly *Magyarosaurus*) were interpreted to support their dwarf status (e.g., Jianu and Weishampel 1999; but see Le Loeuff 2005b) Recent osteohistological studies concur that at least some Haţeg titanosaurs were dwarfed, but these studies also showed that larger-bodied titanosaur taxa were present alongside the dwarfed ones (Stein et al. 2010).

**Mammals.** The first mammals from the Transylvanian Maastrichtian were reported from the Haţeg Basin by Grigorescu (1984) and Grigorescu et al. (1985). Some of these fossils were subsequently described as the new multituberculate *Barbatodon transylvanicum* (sic) by Rădulescu and Samson (1986) (with *Paracimexomys dacicus* Grigorescu & Hahn, 1987 as a subjective junior synonym). The affinities of *Barbatodon* remained poorly understood until the description of a second multituberculate taxon, *Kogaionon ungureanui*, based on much better preserved material (Rădulescu and Samson 1996), as well as more complete remains referred to *Barbatodon* from near the type locality (Csiki et al. 2005; Fig. 10L). These allowed the referral of both of these taxa to their own family Kogaionidae. Additional multituberculate remains, sometimes referred to the kogaionid genera *Hainina*, *Barbatodon*, or *Kogaionon*, were described from the Haţeg Basin (Csiki and Grigorescu 2000; Codrea et al. 2002; Smith et al. 2002; Vasile 2008; Vasile et al. 2011a; T. Smith and V. Codrea, personal communication, 2003). The wider Transylvanian distribution of the group was indicated by similar reports from the Rusca Montană (Codrea et al. 2012) and southwestern Transylvanian (Codrea et al. 2010a; Csiki-Sava et al. 2012; Codrea et al. 2014; Vremir et al. 2014) basins. As far as is currently known, all multituberculates from the Transylvanian area belong to Kogaionidae, which is an endemic European clade.

Unequivocal records of metatherians or eutherians are absent from the Transylvanian Maastrichtian. One isolated, fragmentary tooth from the Haţeg Basin may belong to a therian (Z.Cs.-S., unpublished data), but this needs to be substantiated, as this specimen might represent a case of sample contamination.

## Discussion and Conclusions

**Figure 11.**
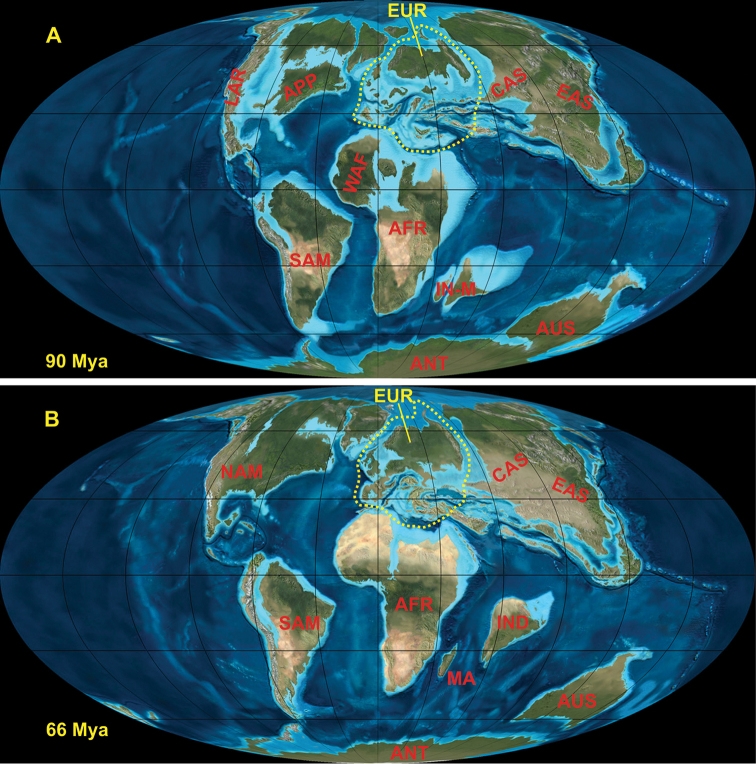
Continental paleogeography of the Late Cretaceous, highlighting the position of the European paleobioprovince (yellow dotted line). **A** Global paleogeography during the relative sea-level highstand period of the Turonian, showing maximum geographical fragmentation of Europe **B** Global paleogeography during the relative sea-level lowstand period of the latest Maastrichtian, showing significant extension of emergent areas. Abbreviations: **AFR** Africa; **ANT** Antarctica; **APP** Appalachia; **AUS** Australia; **CAS** Central (or Middle) Asia; **EAS** Eastern Asia; **EUR** Europe; **IN** India; **IN-M** Indo-Malagasy Landmass; **LAR** Laramidia; **MA** Madagascar; **NAM** North America; **SAM** South America; **WAF** western Africa. Base maps courtesy of R. Blakey.

Despite the rather poor and discontinuous nature of the European fossil record of Late Cretaceous continental vertebrates, especially when compared to the substantially richer and more continuous ones from elsewhere in the world (e.g., Benton et al. 2000; Novas 2009; Titus and Loewen 2013), it holds critically important information on vertebrate evolution and paleobiogeography. This is largely for three reasons. First, Europe was located at the crossroads of other major paleobiogeographic realms of the Late Cretaceous (Fig. 11) and was intermittently connected to some of these other provinces, making Europe a potentially important corridor for continental vertebrate dispersals. Second, Europe had an extremely fragmented geography compared to other major continental landmasses of the Late Cretaceous (Figs 2, 3, 11), making Europe an intriguing location for understanding how continental ecosystems and faunas of this time responded to habitat fragmentation and insular environments. Third, the widespread presence of interfingering marine and continental sedmentary deposits in the European Upper Cretaceous allows the vertebrate-bearing continental units to be tied into the standard subdivisions of the geological time scale. As a result, Europe offers a stratigraphically relatively well-constrained temporal succession of Late Cretaceous faunal assemblages the study of which promises to contribute to long-standing debates on the tempo, mode, and causes of Late Cretaceous faunal evolution and possibly the end-Cretaceous extinction event.

In conclusion, therefore, we argue that the study of the Late Cretaceous continental vertebrates from Europe has incredible potential to offer important insights into: 1) Late Cretaceous faunal evolution and paleobiogeography; 2) Late Cretaceous island life; and 3) the end-Cretaceous extinction event. We discuss these topics separately below.

### Late Cretaceous faunal composition, evolution and paleobiogeography

There is long-standing agreement that Europe represented a separate and distinctive paleobiogeographical realm during the Late Cretaceous (e.g., Nopcsa 1923a, 1934; Le Loeuff 1991, 1997; Holtz et al. 2004a; Pereda-Suberbiola 2009; Carrano 2012). However, despite this general opinion, there are still debates about the origin, detailed affinities, and evolution of the European continental faunas. Whereas some authors have emphasized the Gondwanan affinities of Late Cretaceous European faunas (e.g., Buffetaut et al. 1988; Buffetaut 1989a; Le Loeuff 1991; Rage 1996), others have pointed out the importance of local, vicariant faunal evolution (e.g., Weishampel et al. 1991; Csiki 1997; Pereda-Suberbiola 2009), and, more recently, it has been proposed that the European faunas show important Asiamerican influences (Martin et al. 2005; Prieto-Márquez and Wagner 2009; Csiki et al. 2010b; Ősi et al. 2010a; Makádi 2013b; Prieto-Márquez et al. 2013). With the accelerated pace of new fossil discoveries from Europe in recent years, especially from the Santonian to Maastrichtian, it appears that the origin of the Late Cretaceous continental assemblages from Europe is even more complex than previously assumed.

#### Faunal composition: distribution, endemism and provinciality

The distinctiveness of the European paleobiogeographic province during the latter part of the Late Cretaceous is indicated by the presence of numerous genera (and in some cases suprageneric taxa) unique to Europe (Le Loeuff 1991; Holtz et al. 2004a; Pereda-Suberbiola 2009; Weishampel et al. 2010). These include palaeobatrachid frogs, various chelonians (*Kallokibotion*, dortokids, solemydids), crocodyliforms (hylaeochampsids), dinosaurs (rhabdodontids, struthiosaurines), and mammals (lainodontines, kogaionids). Meanwhile, several higher-level taxa well represented in other paleobiogeographic realms of the latest Cretaceous appear to be definitively absent in Europe, or else their presence has yet to be documented conclusively. These include such iconic groups as tyrannosaurid, oviraptorosaurian, therizinosauroid and troodontid theropods, derived ceratopsid ceratopsians, ankylosaurid ankylosaurs, non-ziphosuchian notosuchian crocodyliforms, and various eucryptodiran turtle groups like trionychoids and testudinoids. Together, the prevalence of certain endemic clades and the absence of otherwise geographically widespread clades establish the uniqueness of the Late Cretaceous European bioprovince.

Representatives of the hallmark European taxa are widespread across Europe and present in the most important latest Cretaceous sites of the realm. For example, dortokids (e.g., Rabi et al. 2013a), hylaeochampsids and close relatives (e.g., Martin and Delfino 2010; Puértolas-Pascual et al. 2014), atoposaurids (e.g, Martin et al. 2014), rhabdodontids (Ősi et al. 2012a; Vremir et al. 2014), and struthiosaurines (e.g., Ősi et al. 2014a) have a trans-European distribution in the latest Cretaceous. Some individual endemic genera also were widely distributed. Chief among these is the nodosaurid *Struthiosaurus*, which ranges from the Santonian to the Maastrichtian, and from Spain to Hungary and Romania (Ősi and Prondvai 2013; Ősi et al. 2014a). Transcontinental and/or multi-state ranges are also documented for the ziphosuchian crocodyliform *Doratodon* (Santonian to Maastrichtian, extending from eastern Spain to Romania), the bothremydid pleurodire *Foxemys* (Santonian of Hungary to upper Campanian–lower Maastrichtian of southern France), the polyglyphanodontine borioteiioidean *Bicuspidon* (Santonian of Hungary to Maastrichtian of Romania), and the eusuchians *Acynodon* (Campanian–Maastrichtian of France, Spain, and Italy to Maastrichtian of Romania) and *Allodaposuchus* (Campanian–Maastrichtian of France and Spain to Maastrichtian of Romania).

That said, however, other major clades are only found on certain island blocks. For example, palaeobatrachid anurans are found on the Ibero-Armorican landmass and in Hungary but not in Romania. Solemydid turtles seem to be restricted to the Ibero-Armorican landmass, whereas the basal meiolaniform *Kallokibotion* (and related taxa) are reported only from the more eastern Tethyan Austroalpine and Transylvanian areas. Madtsoiid snakes have a disjunct distribution, with members described from some of the westernmost (Spanish) and of the easternmost (Romanian) sites, but apparently absent from all intervening landmasses (eastern Spain, southern France, Austria, Hungary). Abelisaurid theropods were present in Ibero-Armorica (*Arcovenator*, *Tarascosaurus*) and on the Austroalpine and Rhenish-Bohemian landmasses, but have yet to be found in the relatively well-sampled Transylvanian region. Among mammals, the Ibero-Armorican areas have yielded exclusively lainodontine zhelestid eutherians, but in Transylvania only kogaionid multituberculates have been uncovered.

These examples show that many, but not all, higher-level taxa and some genera had wide distributions in latest Cretaceous Europe that sometimes encompassed over 15 million years of time and over 2000 kilometers of distance (Fig. 3). However, at lower taxonomic levels (such as the species-level), local endemism seems to be the rule within the European bioprovince. Such endemism is likely due to both geography (faunas being separated from each other spatially) and time (faunas being of slightly different ages). This taxonomic distinctiveness was thoroughly documented in the case of the Romanian assemblages by Weishampel et al. (2010), but was also noted for other faunas (Le Loeuff and Buffetaut 1995; Ősi et al. 2012b). There is no species-level taxon that can be reliably documented from more than one distinct European region. Furthermore, many common higher-level endemic taxa are represented by different genera and species on the different landmasses. Among the widespread rhabdodontid ornithopods, *Mochlodon* occurs on the Austroalpine Block, whereas its relatives *Zalmoxes* and *Rhabdodon* lived on the Transyvanian (Romania) and Ibero-Armorican (southern France, Spain) landmasses, respectively. Dortokids are represented by *Dortoka* on the Ibero-Armorican landmass, but by a different taxon (or possibly multiple taxa) on the Transylvanian and Austroalpine landmasses (Rabi et al. 2013a).

**Figure 12.**
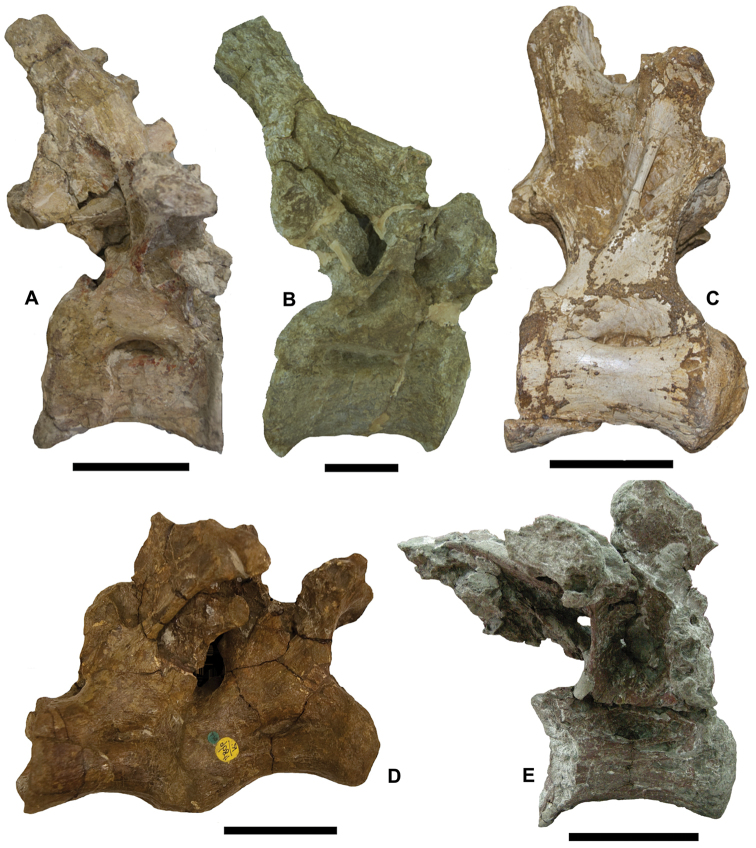
Diversity of Late Cretaceous European titanosaurs, as illustrated by posterior dorsal vertebral size and morphology (all specimens figured in right lateral view, unless specified otherwise). **A** *Atsinganosaurus velauciensis* (VBN 93.01), late Campanian, Velaux-La Bastide Neuve, Bouches-de-Rhône, southern France **B** *Ampelosaurus atacis* (MDE C3-247), late Campanian–early Maastrichtian, Bellevue, Aude, southern France **C** *Lirainosaurus astibiae* (MCNA 7443), late Campanian–early Maastrichtian, Laño, Basque Country, northern Spain **D** *Magyarosaurus dacus* (NHMUK R.4896, reversed), Maastrichtian, Sânpetru, Haţeg Basin, Romania **E** *Paludititan nalatzensis* (UBB NVM1-43), Maastrichtian, Haţeg Basin, Romania. Scale bars equal 10 cm in **A–C** and **E** and 5 cm in **D.** Photographs **A–D** courtesy by Verónica Díez Díaz.

Present alongside the European endemic taxa were some taxa that had a more cosmopolitan (and sometimes global) distribution. Although these higher-level taxa can be found outside of Europe, the individual members of these groups demonstrate a high level of local endemism within Europe. Titanosaurs are a prime example. These sauropods are well-known from the southern continents during the latest Cretaceous, but are also present in Europe (Fig. 12), where different taxa are present in the well-sampled faunas of Transylvania (*Magyarosaurus*, *Paludititan*, possibly additional genera) and the Ibero-Armorican landmass (*Ampelosaurus*, *Lirainosaurus*, *Atsinganosaurus*, possibly additional unnamed taxa). Meanwhile, titanosaurs are conspicuously absent from the Austroalpine landmass. Other examples are dromaeosaurids, with *Balaur* present in Romania and *Variraptor* and *Pyroraptor* in southern France, as well as flying vertebrates such as azhdarchid pterosaurs and enantiornithine birds, which are represented by different taxa in Romania (*Hatzegopteryx*, *Euroazhdarcho*), Hungary (*Bakonydraco*, respectively *Bauxitornis*), and the Ibero-Armorican landmass (*Azhdarcho*, respectively *Martinavis*). This is despite the theoretically great dispersal potential of these flying animals, which should be better able to cross large distances and marine barriers than non-flying species. And finally, the greatest localized diversity of globally widespread clades in Europe is seen in hadrosauroids. Not only are all known genera restricted to small parts of Europe (even within the Ibero-Armorican landmass), different higher-level groups are present in different areas (e.g., non-hadrosaurid hadrosauroids in Romania and Italy; aralosaurine lambeosaurs in the north-Pyrenean areas; tsintaosaurine and lambeosaurine lambeosaurs together with non-hadrosaurid hadrosauroids south of the Pyrenees).

Keeping with the discussion of local endemicity, it is important to note that all of the major Late Cretaceous European land areas possess some taxa that are found only on those particular landmasses. For instance, the struthiosaurine *Hungarosaurus*, the polyglyphanodontin *Distortodon*, and the hylaeochampsid *Iharkutosuchus*, among several other taxa, are only recorded in the Santonian of Hungary. The early Campanian fauna of Austria yields the only known Late Cretaceous choristoderes from Europe. Farther to the west, the Ibero-Armorican assemblages are characterized by the unique presence of batrachosauroidid urodeles, amphisbaenian and/or anguid squamates, derived alethinophidian snakes, solemydid turtles, lambeosaurine hadrosaurs, zhelestid eutherians, and the bizzare large flightless bird *Gargantuavis*. At the other end of the European Archipelago, the Transylvanian faunas stand out because of the presence of non-hadrosaurid hadrosauroids, the possible occurrence of alvarezsauroids and ornithuran birds, and especially because of the abundance and diversity of kogaionid multituberculates. Such extreme local endemicity also extends to some of the more poorly sampled European regions: certain derived non-hadrosaurid hadrosauroids (*Tethyshadros*) are present only in Italy, leptoceratopsids are seen only in the Campanian of southern Sweden, and potential herpetotheriid and/or pediomyid/peradectid metatherians (*Maastrichtidelphys*) are solely recorded in the Maastrichtian of the Dutch-Belgian region. Finally, it is possible that Maastrichtian ornithomimosaurian theropods have been found in Bulgaria, and therizinosauroids and oviraptorosaurs of the same age in Poland. None of these higher-level taxa is currently known from other European areas.

There is even evidence for smaller-scale faunal heterogeneity within some of the European landmasses. The best example concerns the Iberian and French assemblages, particularly during the late Campanian–Maastrichtian. While madtsoiid and alethinophidian snakes are restricted to southern Pyrenean areas, batrachosauroidid urodeles have been reported only from the northern Pyrenean region. The titanosaur *Atsinganosaurus* and the aralosaurin hadrosaur *Canardia* are only reported from southern France, whereas the tsintaosaurin *Pararhabdodon* and the lambeosaurins *Arenysaurus* and *Blasisaurus* are restricted to Spain. Similarly, the basal eusuchian *Massaliasuchus* is present exclusively in Provence (southern France), whereas the crocodyloid *Arenysuchus* is restricted to south of the Pyrenees. Among the lainodontine zhelestids, *Lainodon* appears only in Spain and Portugal, whereas *Valentinella* and *Mistralestes* occur exclusively in southern France. Such faunal differences are especially noteworthy since other members of the same higher-level taxa have trans-Pyrenean ranges, such as the crocodyliforms *Acynodon* and *Allodaposuchus*, the basal ornithopod *Rhabdodon*, the titanosaur *Ampelosaurus*, and the zhelestid *Labes*. Although preservational, taphonomic, and collecting biases could conceivably explain some of these observations, as is always the possibility with a patchy record, the different distributional patterns observed suggest not only the effects of geographic barriers restricting dispersal (such as the rising Pyrenees in the very latest Cretaceous), but perhaps also that chance played a large role in the origin of the different local faunal assemblages at such fine temporal and geographic scales.

Summarizing, two overarching biogeographic patterns describe Late Cretaceous continental Europe. First, there are several clades that are distinctly European and rare or absent in other parts of the world at this time. Second, within Europe there is a high degree of endemicity between the different island blocks and emergent land areas. These patterns have come to light after more than a century of research.

#### Faunal composition and evolution: the history of research

Understanding the origins of the peculiar latest Cretaceous faunal assemblages from Europe (and the endemic assemblages of the localized landmasses) has been a long-term goal of European vertebrate paleontology ever since Nopcsa (1915, 1923a) first recognized the uniqueness of these assemblages. One of Nopcsa’s greatest contributions was in outlining the marked differences between the Late Cretaceous European faunas and their contemporaneous assemblages from other major landmasses (mainly North and South America, and, to a lesser extent, India and Madagascar). He proceeded to compare these unusual Late Cretaceous faunas to Early Cretaceous faunas from Europe, and hypothesized that the former represented depauperate and “degenerated” descendants of the Early Cretaceous faunas that evolved *in situ*, in isolation on islands separated by the great marine transgressions of the Late Cretaceous.

Since then, this idea that the European faunas of the Late Cretaceous evolved in local isolation from the Early Cretaceous faunas has become a cornerstone concept in vertebrate paleontology. Nevertheless, the relative importance of this *in situ* evolution in the shaping and patterning of the Late Cretaceous faunas was periodically de-emphasized and then re-emphasized among researchers, as new fossil data were collected and local assemblages studied in more detail. More recently, the accumulation of vast amounts of new fossils from the late Campanian–early Maastrichtian Ibero-Armorican assemblages (Buffetaut et al. 1989; Astibia et al. 1990) led to the suggestion that the Late Cretaceous continental faunas of Europe were shaped considerably by southern, Gondwanan immigration events superposed upon the background *in situ* evolution of the Early Cretaceous faunas (e.g., Buffetaut et al. 1988; Buffetaut 1989a; Le Loeuff 1991; Le Loeuff and Buffetaut 1991, 1995; Rage 1996). At the same time, study of the Maastrichtian faunas from Transylvania (particularly the Haţeg Basin fauna) indicated that localized vicariant evolution was the more important driver of faunal evolution, although supplemented with isolated dispersal events from Asiamerican sources (e.g., Weishampel et al. 1991; Csiki 1997).

The newly emerging picture of European continental faunal evolution during the Late Cretaceous, based on important new discoveries all over Europe (as synthesized in this paper), frames a more complex story than recognized by Nopcsa and many researchers of the twentieth century. This complexity is largely due to the now-recognized high levels of local endemism in Late Cretaceous Europe and the widely divergent paleobiogeographic affinities of taxa on different European landmasses (i.e., the Gondwanan affinities of some taxa, the Asiamerican affinities of others). Frustratingly, the middle part of the Late Cretaceous—which appears to have been a critical time in the assembly of the latest Late Cretaceous faunas—remains relatively poorly sampled, which makes testing specific biogeographic hypotheses difficult.

Nevertheless, despite the sometimes poor and always patchy fossil record of continental biotas in Late Cretaceous Europe, enough data exist to draw some basic, preliminary conclusions about the emergence of the well-known and highly distinctive continental faunas of the very latest Cretaceous of Europe. Overall, three major categories of faunal components can be identified in the latest Cretaceous European assemblages: a core of taxa descending from older, Early Cretaceous (or even older) faunal stock of Euramerican or Pangean origin, to which a series immigrants were added during the Late Cretaceous from either southern (Gondwanan) or northern (Asiamerican) sources (Le Loeuff 1991; Pereda-Suberbiola 2009; Weishampel et al. 2010; see also Russell 1994; Le Loeuff 1997). The composition and paleobiogeographic significance of these different faunal components is rather well established by previous work (cited throughout this paper), although newly discovered taxa continuously add new information to the existing scenarios.

#### Faunal composition and evolution: the old European core

The old European core is represented mainly by endemic taxa developed through vicariant evolution from pre-existing, widespread clades with members isolated in Europe after the ‘mid’-Cretaceous tectonic and eustatic events. These include palaeobatrachid and discoglossid frogs, solemydid and dortokid turtles, hylaeochampsid and atoposaurid crocodyliforms, nodosaurid ankylosaurs and ‘megalosaur’-grade tetanurans, all of which are known from older, Early Cretaceous European faunas and have deep phylogenetic histories linking them to close relatives that are considerably older.

Two clades of frogs are part of this ‘European core’. The first of these, palaeobatrachids are known from fossils reported beginning with the Santonian of Hungary and the early Campanian of southern France and continuing up to the late Maastrichtian of northern and eastern Spain. The oldest reports of the group come from the Barremian of Spain (Buscalioni et al. 2008). Recent work indicates that palaeobatrachids are exclusively European, and purported reports of representatives of this group from North America are in error (Wuttke et al. 2012). The second group, discoglossids, is part of a larger Pangean radiation of frogs, which are known from Europe beginning in the Middle Jurassic and remained restricted to, but widely distributed throughout, Laurasia since that time (Evans et al. 1990; Roelants and Bossuyt 2005). Discoglossids first appear along with palaeobatrachids in Europe in the Barremian (Buscalioni et al. 2008), and similar anuran associations are reported from the Santonian of Hungary to the late Maastrichtian of northern Spain.

Two turtle groups are also part of the ‘European core’. Both solemydids (Joyce et al. 2011) and dortokids (Pérez-García et al. 2014) are clades of European origin, and throughout their evolutionary history remained largely restricted to Europe. Their oldest members are Early Cretaceous in age (Berriasian of the UK; Barremian of Spain), and thus, the Late Cretaceous representatives can be confidently recognized as descendants of older, Early Cretaceous European faunas.

A roughly similar temporal and spatial distribution can also be identified in the two ‘European core’ clades of non-crocodylian neosuchians, atoposaurids (e.g., Martin et al. 2010; Lauprasert et al. 2011) and hylaeochampsids (e.g., Buscalioni et al. 2011). Both are considered clades of European origin, stemming from the Late Jurassic (atoposaurids) or the Early Cretaceous (hylaeochampsids). Subsequently, throughout their evolutionary histories, both clades remained almost exclusively European in distribution, and their post-Albian members occur solely in Europe. Taken together, the evidence clearly indicates that these crocodyliforms are descendants of an older, largely endemic European faunal assemblage of the later Early Cretaceous.

One of the two most notable dinosaur clades among the ‘European core’ is Nodosauridae. The European latest Cretaceous nodosaurids have long been considered descendants of a generalized basal nodosaurid stock with a Euramerican distribution (e.g., Weishampel et al. 1991, 2010). This hypothesis was refined with the recent discovery of the Albian nodosaurid *Europelta* in Spain which allowed the recognition of Struthiosaurinae as a distinct European clade of basal nodosaurid ankylosaurs, grouping together the new Spanish taxon with the similarly Albian-aged *Anoplosaurus* from southern England as well as the latest Cretaceous *Struthiosaurus* and *Hungarosaurus* from the European archipelago (Kirkland et al. 2013). Struthiosaurinae was identified as a clade with a probable European origin, which separated from contemporaneous North American taxa by vicariant evolution during the middle-late Early Cretaceous, and subsequently diversified in Europe.

There are other potential members of the ‘European core’, but their distribution and evolution is more poorly understood. These include meiolaniform turtles, basal ornithopod dinosaurs, and multituberculate mammals. The rhabdodontid ornithopods have yet to be reported reliably from pre-Santonian beds in Europe, despite the fact that some rhabdodontid-like teeth were described from Lower Cretaceous beds from Burgos (Spain; Torcida Fernández-Baldor et al. 2004), so it is not clear whether they were present on the continent before the latest Cretaceous. However, their closest relatives have a predominantly Euramerican distribution, suggesting that rhabdodontids are remnants of an older, more geographically widespread stock as are other ‘European core’ taxa (Weishampel et al. 2003; Ősi et al. 2012a). The same is probably true for the European meiolaniforms (‘kallokibotionins’ of Rabi et al. 2013a), as their phylogenetic position basal to the pleurodiran-cryptodiran split suggests that the line leading to *Kallokibotion* and kin may have originated sometimes before the Late Jurassic, in Pangean times, and therefore been part of a widespread ancestral stock (Sterli and de la Fuente 2013) Finally, the bizarre kogaionid multituberculates of Transylvania are known only from the Maastrichtian, but the stratigraphic distribution of the clades bracketing them suggests a late Early Cretaceous European vicariant origin, as expected of a ‘European core’ group (Csiki and Grigorescu 2006; Weishampel et al. 2010).

#### Faunal composition and evolution: the Asiamerican kinship

It was once thought that several other groups may have been ‘European core’ taxa that originated locally long before the Late Cretaceous. Among these were albanerpetonids and dromaeosaurids (e.g., Le Loeuff et al. 1992; Le Loeuff and Buffetaut 1995; Pereda-Suberbiola 2009). More recently, however, comprehensive and well-resolved phylogenetic analyses have shown that the Late Cretaceous European members of these clades did not have close affinities with those from pre-Cenomanian times of the same continent. Instead, the Late Cretaceous European taxa were often found to have close phylogenetic affinities with Asian and American species (Csiki et al. 2010b; Brusatte et al. 2013a; Sweetman and Gardner 2013; Szentesi et al. 2013), indicating biotic interchange between the European archipelago and Asiamerica during the final 20 to 30 million years of the Cretaceous.

Some of these Asiamerican immigrants seemingly migrated to Europe early in the Late Cretaceous. One such group is the polyglyphanodontine lizards, which are likely of North American origin (Nydam et al. 2007) and reached Europe sometime between the Aptian and Santonian, to account for their presence in the Santonian of Hungary (Makádi 2013a; Nydam 2013). The chamopsiid *Pelsochamops* from the Santonian of Hungary, the only known European representative of the clade (Makádi 2013b), might have had a similar biogeographic history.

Similar early immigration into Europe may account for the presence of lainodontine zhelestids, as recent phylogenetic analyses indicate that the ancestors of the Campanian-Maastrichtian European taxa dispersed from Central Asia during the early Late Cretaceous, probably sometime around the Cenomanian-Turonian interval (Archibald and Averianov 2012; Gheerbrant and Astibia 2012). Other vertebrate groups apparently traveled westward into Europe at this time as well, including the ancestors of the Santonian ‘bagaceratopsid’ *Ajkaceratops* from Hungary (Ősi et al. 2010a) and the basal neoceratopsian *Craspedodon* from the Santonian of Belgium (Godefroit and Lambert 2007; Chinnery-Allgeier and Kirkland 2010), and possibly that of the basal marsupialiform *Arcantiodelphys* from the Cenomanian of western France (Vullo et al. 2009b). The arrival of these taxa in Europe during the first part of the Late Cretaceous suggests that, despite rising sea levels and advanced continental fragmentation (see Fig. 11), at least some faunal interchange was still possible between Europe and Asiamerica.

Faunal connections between Europe and Asiamerica evidently continued well into the Late Cretaceous. This is perhaps surprising, given the completion of the Turgai Strait (separating Europe from Central and Eastern Asia beginning with the Turonian), the progressive opening of the North Atlantic, and the transgression of the Western Interior Seaway (dividing North America into an eastern Appalachian and a western Laramidian landmass after the latest Albian).

Many of these Late Cretaceous faunal connections were with North America. The basal alligatoroids *Musturzabalsuchus* and *Massaliasuchus* from the Campanian of the Ibero-Armorican landmass likely resulted from North American dispersals, because the basal members of this clade (e.g., *Leidyosuchus*, *Deinosuchus*) have a North American distribution (e.g., Brochu 1999). A similar North American origin was also suggested for the basal crocodyloid *Arenysuchus* from the Maastrichtian of northern Spain (Puértolas et al. 2011). The timing of these dispersal events could have been different: around the earliest Campanian at the latest for the alligatoroids, but possibly as late as mid-Maastrichtian for the crocodyloids. The appearance of derived, lambeosaurin lambeosaurines such as *Arenysaurus* and *Blasisaurus* in northern Spain during the late Maastrichtian also was likely the product of dispersal, as their closest relatives are from North America (Prieto-Márquez et al. 2013); however, a possibly Asiatic origin of the latest Cretaceous lambeosaurines in Europe was also suggested by Cruzado-Caballero et al. (2013). Finally, the arrival of metatherians in the late Maastrichtian of western Europe (*Maastrichtidelphys*) also may represent a latest Cretaceous dispersal event of North American origin.

These and other examples suggest that biotic interchange between North America and Europe were intermittently possible throughout the Late Cretaceous. These dispersals likely followed high-latitude dispersal routes such as the De Geer corridor, which was active as of the ‘mid’-Maastrichtian (Brikiatis 2014) and would have allowed dispersal of crocodyloids, lambeosaurines and metatherians between the two landmasses towards the end of the Maastrichtian. Under the influence of climatic and eustatic changes of the Late Cretaceous, the high latitudinal position of such dispersal corridors may have exerted a strong filtering effect, perhaps explaining the rarity and taxonomic randomness of the migration events and why certain signature latest Cretaceous North American taxa such as tyrannosaurid and ceratopsid dinosaurs are not seen in Europe.

Other faunal connnections are evident between Europe and Asia during the latest Cretaceous, and these are only recently becoming better understood. The most conspicuous cases involve different clades of hadrosauroid dinosaurs. First, representatives of the post-Coniacian radiation of derived non-hadrosaurid hadrosauroids such as *Telmatosaurus* and *Tethyshadros* probably stemmed from an Asian dispersal sometime during the Santonian–Campanian, to account for their presence in eastern Europe during the late Campanian-Maastrichtian (e.g., Dalla Vecchia 2009a; Sues and Averianov 2009; Prieto-Márquez 2010b). Second, two late Maastrichtian lambeosaurines from Ibero-Armorica, the aralosaurin *Canardia* and the tsintaosaurin *Pararhabdodon*, have older (Santonian–Campanian) sister-taxa in Asia, suggesting that their ancestors dispersed from Asia around the ‘mid’ Maastrichtian (Prieto-Márquez and Wagner 2009; Prieto-Márquez et al. 2013). Similarly, the presence of the derived dromaeosaurid *Balaur* in the Maastrichtian of Romania, nested in a clade of Asiamerican taxa, may indicate an immigration event of Asiatic origin after the mid-Campanian (Csiki et al. 2010b; Brusatte et al. 2013a). Santonian–Campanian European ceratopsians may have shared a similar dispersal history, and Asian-European interchange could also explain the presence of putative alvarezsaurids (*Heptasteornis*) and possible footprints of oviraptorosaurs and therizinosauroids.

Although there is clear evidence for Asian-European faunal connections in the latest Cretaceous, pinpointing the timing, and particularly the exact paths, of these faunal interchange events is difficult. This is especially true because the Turgai Strait represented a significant marine barrier between the two land areas, starting in the Turonian (Smith et al. 1994). Island hopping along chains of volcanic archipelagoes bordering the northern margin the Tethys Ocean was considered a viable dispersal scenario by Dalla Vecchia (2009a). Many other scenarios are also possible, but all of them must in some way include island-hopping or dispersal across fairly long stretches of water.

#### Faunal composition and evolution: the Gimmigrants

Along with biotic connections with Asiamerica, there is also strong evidence that interchange with Gondwana dramatically shaped the European Late Cretaceous faunas (e.g., Buffetaut et al. 1988; Buffetaut 1989a; Le Loeuff 1991; Le Loeuff and Buffetaut 1995; Rage 1996). The Europe-Gondwana relationship was formally recognized with the definition of the ‘Eurogondwanan’ (Le Loeuff 1991) or ‘Atlantogean’ (Ezcurra and Agnolín 2012) biogeographic provinces, which link these two regions in the Late Cretaceous (see also Gheerbrant and Rage 2006; Rabi and Sebők in press). Many vertebrate groups have been cited to support this relationship during the Campanian–Maastrichtian, including characiform, lepisosteiform and mawsoniid fishes, neobatrachian frogs, bothremydid and podocnemidid turtles, madtsoiid and boiid snakes, trematochampsid and ziphosuchian crocodyliforms, and abelisaurid and titanosaurian dinosaurs (e.g., Le Loeuff 1991; Le Loeuff and Buffetaut 1995; Gheerbrant and Rage 2006; Pereda-Suberbiola 2009).

Before reviewing the strong evidence for European-Gondwanan links, it must be pointed out that several of the earlier assertions of faunal similarities between these landmasses rest on somewhat dubious grounds. No podocnemidid turtle remains have been reliably described from the Upper Cretaceous of Europe (Lapparent de Broin and Murelaga 1999; Pereda-Suberbiola 2009), and the alleged trematochampsid *Ischyrochampsa* (Vasse 1995) is currently considered an eusuchian with unknown affinities. Similarly, the presence of boiid snakes in the Late Cretaceous of Europe, suggested previously by Rage (1981), cannot be reliably supported (see also Pereda-Suberbiola 2009), and the madtsoiid snakes of Europe may represent more ancient holdovers rather than latest Cretaceous Gondwanan immigrants, two conflicting scenarios that are currently difficult to test because of poorly constrained phylogenies (Vasile et al. 2013). Definitive southern influence is rather weakly supported even in the case of the titanosaurs, usually regarded as a Gondwanan taxon and often cited as southern immigrants in Late Cretaceous Europe (e.g., Astibia et al. 1990; Le Loeuff 1993). It appears that at least some Campanian–Maastrichtian European titanosaurs are relicts, reminiscent of much earlier (Early Cretaceous) taxa (e.g., *Atsinganosaurus*; Garcia et al. 2010); even more derived lithostrotians (*Lirainosaurus*) do not appear closely related to more or less contemporaneous Gondwanan taxa (Curry Rogers 2005). Until more precise information on the phylogenetic affinities of Late Cretaceous European titanosaurs becomes available, it seems premature to consider these as southern, Gondwanan elements.

There is, however, considerable evidence for biotic interchange between Europe and Gondwana during the latest Cretaceous. Fishes are some of the most important Gondwanan-derived components of the European assemblages. Characiform fishes are reliably established as originating in Gondwana during the early Late Cretaceous (Turonian; e.g., Arroyave et al. 2013), and their appearance in the Maastrichtian of Romania and southern France most likely resulted from immigration during the Coniacian–Campanian. Additionally, the lepisosteiform *Atractosteus africanus*, described previously from the Upper Cretaceous (“Senonian”) of Africa, was also reported from the lower Campanian of southern France, suggesting its northbound dispersal sometime during the Coniacian–Santonian (Cavin et al. 1996). Finally, the presence of freshwater-dwelling mawsoniid coelacanths in the lower Maastrichtian of southern France documents another pre-Maastrichtian dispersal event into Europe from the south (Cavin et al. 2005). Whether the migration of these three groups happened during the same time interval or represent different, unconnected dispersal events is currently difficult to establish with certainty, but at least two immigration pulses appear most likely. These dispersals probably required the establishment of land bridges that allowed fluvial connections between Europe and Gondwana, as it is thought that these fishes did not have strong saltwater tolerance based on comparisons to their living relatives and their phylogenetic affinities among freshwater taxa (Otero et al. 2008; Brito and Mayrinck 2008; Mayrinck 2010). This in turn suggests that trans-Tethyan continental connections might have been more widespread during the Late Cretaceous than generally acknowledged based on tectonic and sedimentary facies data.

Frogs, turtles and crocodyliforms also support a Europe-Gondwana link. The derived neobatrachian *Hungarobatrachus* from the Santonian of Hungary likely stemmed from a south-north immigration event, as molecular phylogenetic analyses of Anura indicate that the origin and early evolution of Neobatrachia occurred in Gondwana (e.g., Biju and Bossuyt 2003; Bossuyt et al. 2006; Szentesi and Venczel 2010). Similarly, the origin of bothremydid turtles can be traced to the south-Tethyan areas of Gondwana (Gaffney et al. 2006), and thus the appearance and widespread occurrence of this group in the Santonian–Maastrichtian of Europe most likely occurred after dispersal from Gondwana. Rabi et al. (2012) identified two different dispersal events that could explain the European distribution of this group: a pre-Santonian immigration through the eastern parts of the Mediterranean Seuil that led to the introduction of the Foxemydinae, and a second, possibly later (pre-late Campanian) migration towards western Iberia that explains the presence of *Rosasia* in Portugal. Whereas the second dispersal could have involved brackish-water-tolerant taxa (as was also suggested tentatively for characiforms and lepisosteiforms), the foxemydine radiation was purely continental and probably required emergent landmasses to cross from Africa into northern Apulia (Austroalpine Block) in pre-Santonian times, and to subsequently spread towards cratonic Europe (Ibero-Armorican landmass) in pre-Campanian times. In a similar vein, the Gondwanan affinities of the European ziphodont crocodyliform *Doratodon*, already considered a member of the predominantly Gondwanan Ziphosuchia by Company et al. (2005), were currently supported by the phylogenetic analysis of Rabi and Sebők (in press).

Abelisauroid theropods were often considered the paramount evidence for the Gondwanan affinities of the Late Cretaceous European bioprovince (Buffetaut et al. 1988; Buffetaut 1989a; Le Loeuff and Buffetaut 1991). Such an interpretation was based on the almost exclusively Gondwanan distribution of abelisauroids and complete absence of this clade in Laurasia (except Europe during the Cretaceous; Carrano and Sampson 2008). In a recent and comprehensive phylogenetic analysis, Tortosa et al. (2014) recovered all European abelisauroids, including the Albian taxon *Genusaurus*, as abelisaurids. More specifically, they were also able to identify a group of medium-sized European taxa that form a subclade with latest Cretaceous Indo-Malagasy taxa including *Majungasaurus* from Madagascar and *Indosuchus* and *Rajasaurus* from India. This is strong evidence that at least some of the latest Cretaceous European abelisauroids were the product of European-Gondwanan interchange.

In some cases, there is evidence for European taxa being linked to particular regions of Gondwana. For example, *Arcovenator* is more closely related to the Indo-Malagasy abelisaurids than to the South America taxa (Tortosa et al. 2014). This might look surprising on account of the impressive paleogeographic separation of Europe and India/Madagascar during the latest Cretaceous. However, similar affinities are also documented in the case of the African-European adapisoriculid euarchontans from the Paleocene and their closest relative, the Maastrichtian *Deccanolestes* from India (Prasad et al. 2010; Smith et al. 2010), and Indian affinities were also reported in the case of south-Pyrenean neoselachian assemblages (e.g., Soler-Gijon and López-Martínez 1998; Kriwet et al. 2007). Furthermore, recent evidence suggests that the madtsoiid snake *Menarana* was present in both Spain and Madagascar (LaDuke et al. 2010). On the other hand, other taxa show slightly different affinities with particular portions of Gondwana. The European bothremydid turtles, for instance, are more closely related to African-South American clades to the exclusion of the Indo-Malagasy Kurmademydini (Gaffney et al. 2006; Rabi et al. 2012). In conclusion, the Europe-Gondwana paleobiogeographic connections during the latest Cretaceous appear to have been complex.

#### Interactions between Late Cretaceous Europe and other bioprovinces – origin, timing and route of faunal connections

From the above overview, it is clear that the evolution of the Late Cretaceous European faunas was shaped both by local endemic evolution of an older, Early Cretaceous faunal stock and by several different immigration events throughout the Late Cretaceous, originating from different surrounding (or even more distant) landmasses. The endemic stock probably represents the hallmark feature of the Late Cretaceous European bioprovince, differentiating it from other contemporaneous faunal assemblages. This core assemblage evolved in isolation and in some instances diversified taxonomically and ecologically, and contributed to a great extent to the uniqueness of the Late Cretaceous European vertebrate bioprovince.

During the Late Cretaceous, the local evolution of this ‘European core’ was augmented with immigration waves originating from North America, Asia, and Gondwana. These waves introduced newcomer taxa from three different directions: 1) from central and eastern Asia, across the Turgai Strait; 2) from (mainly western) North America, across the Western Interior Seaway and the opening of the Atlantic Ocean; and 3) from Gondwana, across the (Neo)Tethys Ocean. Although these marine barriers were thought to be rather impenetrable to continental faunal dispersal in the Late Cretaceous, it appears that occasionally they could have been breached by different taxa, in form of chance dispersals involving random individual components of the source faunas instead of entire modules (geo-dispersal; Lieberman and Eldredge 1996; Lieberman 2003). The exact timing and route of these dispersals are difficult to establish, but some constrains can be set based on the phylogenetic affinities and temporal and spatial distribution of the clades involved.

There was considerable interchange between North America and Europe during the Late Cretaceous. Based on the available evidence, it appears that only western North American (Laramidian) clades were involved into these dispersal events, whereas the (admittedly much more poorly known) eastern Appalachian faunas do not appear to have contributed to the European faunas. Synthesizing the current information, it seems that albanerpetontids, batrachosauroidid urodeles, polyglyphanodontin and chamopsiid lizards, alligatoroid and crocodyloid crocodyliforms, lambeosaurine hadrosaurids, and ‘peradectid’ metatherians were introduced from Laramidia into Europe after the post-Barremian biogeographical separation of the two bioprovinces. Some North American dispersals must have occurred during the ‘middle’ Cretaceous (albanerpetontids, borioteiioid lizards), whereas others occurred during the Late Cretaceous (batrachosauroidids, alligatoroids), and some during the late Maastrichtian itself (crocodyloids, lambeosaurines, metatherians). The earliest reconstructed dispersal appears to have preferentially led to eastern Europe, whereas the later (post-Santonian) dispersal events were restricted to western Europe. The significance of this pattern is as yet unclear, and it is also possible that it represents the product of random differential survival/extinction in the different European landmasses.

Compared to the North American faunal links, those between Europe and Asia have been outlined only more recently. The most important Late Cretaceous European taxa showing Asian affinities include different ceratopsians, diverse non-hadrosaurid hadrosauroids, basal lambeosaurine hadrosaurids, velociraptorine dromaeosaurids, and zhelestid eutherians. At least three waves of dispersal can be hypothesized based on the current fossil record: one prior to the completion of the Turgai Strait (pre-Turonian) that brought Cenomanian hadrosauroids and zhelestids to Europe, a slightly later one during the Coniacian–Santonian that explains the presence of different ceratopsians and derived non-hadrosaurid hadrosauroids in Europe, and a third sometime in the latest Cretaceous. This third event may in actuality be a series of events, one around the Campanian–Maastrichtian boundary that delivered taxa such as velociraptorines (and possibly alvarezsaurids) into eastern Europe, and a second in the middle Maastrichtian that brought lambeosaurines to western Europe. Minor to moderate relative drops in sea-level are documented both near the Campanian/Maastrichtian boundary and in the middle Maastrichtian in the eastern European epicontinental seaways, which would have been optimal times for such latest Cretaceous range expansions across marine barriers.

Some further patterns seem to characterize the Asian-European faunal dispersals. Movement of groups with relatively lower dispersal ability, such as mammals (due to their small size), is restricted to the earliest Late Cretaceous, before the completion of the Turgai Strait in the Turonian. Subsequent to this major paleogeographic event, only different groups of dinosaurs (with assumedly higher dispersive potential) were able to migrate from Asia to Europe. This stands in contrast with the Laramidian faunal connections, in which small-sized taxa (amphibians, lizards, and mammals) figure more prominently and appear to have taken part in all identified migration events.

Furthermore, taxa introduced from Asia into the more distant, cratonic western European landmasses (aralosaurins, leptoceratopsids, zhelestids) lived preferentially in coastal, mainly mesic continental environments, such as those that dominated the Central Asiatic areas bordering the Turgai Strait and experiencing repeated marine incursions (Nessov et al. 1994). On the other hand, ‘bagaceratopsids’ (and protoceratopsids, in general), velociraptorine dromaeosaurids and mononykine alvarezsaurids are more commonly found in semiarid deposits of Asia (e.g., You and Dodson 2004; Eberth 2010), and in Europe have been reported exclusively from the eastern Tethyan archipelago. This might suggest, as a working hypothesis, that Asian immigration into Europe took place along distinct routes into eastern and western Europe, being controlled by different sets of filtering effects: taxa adapted to more humid environments were able to use coastal plains opened up by sea-level fluctuations in the stable cratonic areas to disperse towards cratonic western Europe, while habitually more continental-bound taxa required firmer emergent land bridges and thus might have used the tectonically active mountain chains of southeastern Europe, northern Anatolia, and the southern margins of Central Asia to move westward into the Tethyan archipelago.

Faunal interactions with Gondwana are the third major source of European immigrants during the Late Cretaceous. Well-supported cases of European taxa with Gondwanan affinities include a series of fish groups (lepisosteiforms, characiforms, and mawsoniid coelacanths), neobatrachian frogs, bothremydid turtles, sebecosuchian crocodyliforms, and derived abelisaurid theropods. One hallmark feature of Gondwana-Europe faunal interchanges in the Late Cretaceous is the immigration of different freshwater fishes which arrived in Europe through at least two dispersals, one pre-Campanian (involving lepisosteiforms), and the other near the Campanian-Maastrichtian boundary (involving characiforms and mawsoniids), both of which probably required establishment of land bridges with fluvial networks linking Europe and Africa. Neobatrachians and foxemydine bothremydids were introduced into Europe probably during the Turonian–Coniacian. Because members of both clades are known in Upper Cretaceous deposits of Africa (e.g., de Broin et al. 1974; Gaffney et al. 2006; Báez et al. 2009), this landmass appears the most plausible starting point for their immigration; the presence of bothremydines in the Middle East may suggest that at least the turtle dispersal occured through an eastern route, across the Anatolian and Apulian platforms (Rabi et al. 2012). Soon after the Santonian, representatives of the foxemydines made their appearance on the Ibero-Armorican landmass, in the lower Campanian of southern France, suggesting that the group successfully colonized the southern European archipelago and was able to spread between the different landmasses despite its terrestrial lifestyle. Sebecosuchian crocodyliforms (*Doratodon*) were also part of this Turonian-Coniacian immigraton wave targeting eastern Europe, but their area of origin is less well constrained; a more distant, westernmost Gondwanan origin appears likely, since members of this group are yet to be reported from Africa while being both abundant and diverse in South America.

The majungasaurine abelisaurids from the Ibero-Armorican landmass are closely related to Indo-Malagasy taxa, suggesting that their ancestors arrived in southwestern Europe from central Gondwana sometime during the late Campanian. The route of this dispersal event is unclear, since it appears to have circumvented the eastern European Tethyan areas to directly reach southwestern Europe. It is possible that Africa was a stepping-stone between Indo-Madagascar and Iberia towards the end of the Cretaceous, but the requisite African abelisaurids supporting such a link have yet to be found. Such an Indo-Malagasy-to-Africa-to Europe immigration pattern has been also hypothesized for the adapisoriculids of the Paleocene (Prasad et al. 2010; Smith et al. 2010).

One of the most interesting patterns emerging from the fossil record is that Europe appears to have been a net receiver of immigrants from North America, Asia, and Gondwana. Faunal interchanges involving ‘European core’ taxa traveling in the opposite directions have yet to be documented. Previous reports of potential European immigration to these areas, such as characiform fishes migrating from Europe to North America (Newbrey et al. 2009), kogaionids represented in Appalachia (eastern North America; Denton et al. 1996) and palaeobatrachids appearing in Laramidia (Estes and Sanchíz 1982a), or European zhelestids having a faunal link with Malagasy taxa (Averianov et al. 2003), cannot be strongly supported, either because the suggested timing of these events occurs before such taxa are recorded in Europe or because material outside of Europe has been incorrectly identified as sharing affinities with European taxa (e.g., Grandstaff et al. 1992; Kielan-Jaworowska et al. 2004; Rose 2006; Wuttke et al. 2012; Denton 2014). Therefore, it appears that the European archipelago was essentially a paleobiogeographic cul-de-sac during the Late Cretaceous, limited to receiving several waves of immigrants but not sharing its ‘core’ taxa with other parts of the world.

#### Late Cretaceous faunal evolution in continental Europe

The first overviews of the Late Cretaceous European faunas made by Nopcsa (1915, 1923a) emphasized two features he perceived as their essential characteristics: faunal homogeneity across Europe, both in space (geographically) and time (stratigraphically), and simple descent from an older, Early Cretaceous stock. The large amount of new data that has become available following Nopcsa’s early reviews, which is synthesized here, offers a radically different view. It suggests an intricate evolutionary history of these European faunas, shaped both by endemic local and regional evolution as well as by a complex array of immigration events, randomly distributed in space and time. Essentially, the local faunas of different European landmasses evolved under different, sometimes completely unrelated constrains and were shaped as much as by historical contingencies (in part depending on their specific paleogeographic position, tectonic setting, or paleoenvironmental conditions) as by overarching factors of control (eustasy, paleoclimatic or biologic evolution).

The major barrier in understanding evolution of the Late Cretaceous continental vertebrate faunas from Europe is the patchiness of the available fossil record. Since Late Cretaceous Europe can hardly be regarded as one contiguous and homogenous paleobioprovince (e.g., Buffetaut and Le Loeuff 1991; Rage 2002; Pereda-Suberbiola 2009; Weishampel et al. 2010), using faunal information derived from one age and one particular landmass alongside that derived from another age and landmass (i.e., Santonian of Hungary to Maastrichtian of Romania) to infer major general trends in the Late Cretaceous faunal evolution of the bioprovince is fraught with uncertainty. Nevertheless, even with this caveat, it is possible to recognize some evolutionary events and even infer some broader trends. Furthermore, since the Ibero-Armorican landmass appears to have the most complete and continuous continental vertebrate fossil record from Late Cretaceous Europe, it can be used as a temfig for comparison to faunal changes on other landmasses. Finally, comparing European data to those derived from other major continental landmasses of the Late Cretaceous may also complement our understanding of Late Cretaceous European faunal evolution.

The Cenomanian faunas (especially the better known ones of western Europe) mark the beginning of the transition from more widespread, Euramerican or Neopangean faunal assemblages to those typical of the Late Cretaceous. As emphasized by Vullo et al. (2007), the appearance of taxa such as basal hadrosauroids or basal marsupialiforms in the European fossil record marks the emergence of typical Late Cretaceous faunas. The presence of a marked faunal change around the Early/Late Cretaceous boundary, during which a more typical Late Cretaceous assemblage begins to appear, has also been reported in western North America (e.g., Cifelli et al. 1997; Kirkland et al. 1997; Jacobs and Winkler 1998) and South America (e.g., Coria and Salgado 2005; Calvo et al. 2006), and a similar change might be also recognized in eastern Asia. Despite this rough temporal coincidence, the causes of the ‘mid’-Cretaceous faunal turnover were probably different in the different landmasses. In North America, it appears to have been related to tectonic factors that established land connections with Asia through Beringia, and thus enabled large-scale faunal invasions towards Laramidia (e.g., Cifelli et al. 1997), whereas in South America it may have been due to floral changes linked to the rise of the angiosperms, although this is far from certain (e.g., Coria and Salgado 2005). The causes of the turnover in Europe are also currently unclear, but they might be related with the prolongation of Aptian-Albian faunal connections with Asia, connections suggested previously by Upchurch et al. (2002).

It is remarkable, nonetheless, that despite the incipient faunal turnover the Cenomanian faunas of Europe essentially retain an Early Cretaceous composition. Both solemydid and dortokid turtles are already known from the late Early Cretaceous of the bioprovince (Joyce et al. 2011; Pérez-García et al. 2014), as are all major Cenomanian crocodyliform clades (e.g., Martin and Delfino 2010). These Cenomanian assemblages also extend the Early Cretaceous fossil record of ornithocheirid pterosaurs (Barrett et al. 2008), as well as that of dromaeosaurids (Sweetman 2004), carcharodontosaurids (Ortega et al. 2010), nodosaurids (Blows 1998; Kirkland et al. 2013; Blows and Honeysett 2014), and titanosauriforms (Le Loeuff 1993; Le Loeuff et al. 2013) among dinosaurs, all these clades well represented during earlier timeslices of in Europe. Although some of these taxa eventually disappear soon after the Early/Late Cretaceous boundary (e.g., carcharodontosaurids, primitive hadrosauroids), most of the clades positively identified in the European Cenomanian will continue to evolve through the Late Cretaceous and some will even eventually survive the Cretaceous-Paleogene boundary extinction event.

The Turonian–Coniacian is still the ‘dark age’ of Late Cretaceous Europe. Despite its extremely poor fossil record, this interval must have witnessed the birth of the unique, endemic vertebrate assemblages of latest Cretaceous Europe. Although fossils are rare, it is clear that “ghost lineages” of many typical European clades – palaeobatrachids, solemydids, dortokids, atoposaurids, hylaeochampsids, struthiosaurines – must have been evolving *in situ* during this time, because members of these groups are reported both from older and from younger deposits in Europe. Based on interpretation of the footprint record, sauropods (most probably titanosaurs) were also surviving in the eastern European Tethyan archipelago, even if these might have disappeared in western, cratonic Europe. During the Turonian–Coniacian, these incumbent European clades were continuing to evolve and likely were giving rise to endemic subgroups that would characterize the latest Cretaceous.

At the same time that European clades were evolving *in situ*, the European faunas were remodeled to a great extent by immigration. Interchange with Asia most likely brought zhelestids during the Turonian and bagaceratopsids, derived non-hadrosaurid hadrosauroids, and perhaps leptoceratopsids during the Coniacian–?early Santonian (Sereno 2000; Sues and Averianov 2009; Prieto–Márquez 2010a,b; Averianov and Sues 2012).

Such faunal exchange probably coincided with periods of significant sea-level fall of the Turgai Strait during the early Turonian and late Coniacian (Baraboshkin et al. 2003), allowing the westward dispersal of zhelestids, neoceratopsians, and derived non-hadrosaurid hadrosauroids.

Faunal exchanges also intensified with Gondwana and Laramidia during this time, introducing neobatrachian frogs, ancestors of foxemydin bothremydids, derived albanerpetontids, borioteiioid lizards, and other groups. These dispersals apparently targeted the eastern European islands rather than the western cratonic areas and most involved small-bodied taxa. This might suggest the presence of northerly dispersal routes from North America that circumvented western Europe through the Fennosarmatian and Ukrainean landmasses (e.g., Ziegler 1988), preconfiguring the De Geer Route of Brikiatis (2014). Existence of such high-latitude routes is concordant with paleoclimatic and paleooceanographic data indicating a climatic maximum from the Cenomanian to the Coniacian (e.g., Norris et al. 2001) and a significant sea-level drop in the North Atlantic-Arctic regions during the mid-Turonian (Miller et al. 2003).

Regardless of the exact details of this largely inferred Turonian–Coniacian evolutionary stage, it is clear that by the beginning of the Santonian the typical European latest Cretaceous assemblages were becoming well established across the continent. By this time, dortokids, hylaeochampsids and struthiosaurines spread towards the eastern European Tethyan archipelago, and ‘kallokibotionins’ and rhabdodontids were also present. Moreover, intraclade diversification of the hallmark European group Rhabdodontidae was also underway, with the separation of distinct western (*Rhabdodon*) and eastern (*Mochlodon*, *Zalmoxes*) phylogenetic lines (Ősi et al. 2012a). Together with immigrants from different sources, this European endemic core built up the first reasonably well-known later Late Cretaceous vertebrate fauna, that from the Santonian of Iharkút, Hungary (see section **B**, above), which offers a glimpse into the composition of the European continental vertebrate faunas after about 15 million years of evolution in relative isolation. Unfortunately, as Iharkút yields the only well-documented example of European Santonian faunas, dissimilarities in faunal composition across Europe and existence of possible intra-European provinciality are still difficult to detect. Nevertheless, because the Hungarian fauna lacks leptoceratopsids and hadrosauroids, both taxa documented from coeval but more poorly sampled deposits elsewhere in Europe, it seems as if at least some provinciality was occurring.

During the Santonian, faunal connections between Europe and other landmasses persisted, although with reduced intensity compared to pre-Santonian times. Apparently, faunal exchanges with Asia ceased by the Santonian, in accordance with regional paleogeographic and sedimentological data that support a generalized drowning of the Russian Platform and Turgai Strait areas starting in the Santonian, and a peak transgression beginning in the early Campanian and continuing into the Maastrichtian (Baraboshkin et al. 2003; see also Miller et al. 2003), all of which would have complicated interchange across land.

Only a few taxa, such as some lepisosteiforms, appear to have been introduced from Africa during the Santonian, and despite a major drop in North Atlantic sea-level during this time (Miller et al. 2003), few, if any, migrants seem to have arrived from North America. Unlike during the Turonian–Coniacian, the Santonian arrivals seem to have targeted exclusively the western European cratonic areas, where some of these immigrant groups (such as alligatoroids) remained for the rest of the Late Cretaceous. Based on the current evidence, which is admittedly scanty, it seems as if the more westerly Alboran-Iberian route was used for trans-Tethyan dispersals during the Santonian, whereas a more southern route (a predecessor of the Thulean Route; Brikiatis 2014) operated across the Atlantic, between North America and the Ibero-Armorican landmass.

The early Campanian European faunas are rather similar to the Santonian ones, save for the appearance of additional Gondwanan and North American taxa in western Europe. These faunas are characterized by a generalized foxemydin-struthiosaurine-rhabdodontid composition, a core component of the typical Late Cretaceous European pattern (e.g., Holtz et al. 2004a). It has often been emphasized that titanosaurs, another hallmark European taxon within Laurasia, are missing in the known pre-late Campanian faunal assemblages (e.g., Le Loeuff 1993; Le Loeuff and Buffetaut 1995; Buffetaut et al. 1997b), and this absence was used to suggest the presence of a pre-late Campanian ‘sauropod hiatus’ in Europe, or alternatively the exclusion of titanosaurs from the coastal, ‘estuarine’ environments that are represented by the major early Campanian localities of Muthmannsdorf (Austria) and Villeveyrac (southern France). However, footprints document the presence of sauropods (probably titanosaurs) in the Santonian of the eastern European Tethyan archipelago (Nicosia et al. 2000b), and it is thus possible that their absence in certain landmasses is due to local faunal differences and/or paleoecological segregation instead of a regional extinction event.

The early Campanian faunas demonstrate significant faunal disparity between the different landmasses of the European archipelago. The very poor Swedish record shows the continued presence of possible leptoceratopsids in the northern cratonic landmasses of Europe (Fennosarmatia). Meanwhile, the southwestern cratonic European assemblages have yielded palaeobatrachid frogs, foxemydin bothremydid and possible solemydid turtles, basal alligatoroids and small-sized abelisauroids, as well as core European taxa (struthiosaurines and rhabdodontids); exotic elements include the Gondwanan lepisosteiform and the North American basal alligatoroid immigrants. Finally, the Tethyan, Austroalpine record of the early Campanian documents a high degree of faunal continuity with the older Hungarian assemblages, as supported by the presence of kallokibotionin and dortokid turtles, ‘megalosaur’-grade tetanurans, struthiosaurines, rhabdodontids and azhdarchids, which even occasionally are the same genera (*Struthiosaurus*, *Mochlodon*). It appears, therefore, that the major continental landmasses of early Campanian Europe already hosted distinct faunal assemblages, which are variants of the same basic faunal temfig of this island archipelago that have been shaped by local evolution and occasional faunal exchanges within Europe and/or with other continents. Furthermore, the Santonian–early Campanian Austroalpine faunas provide the first opportunity to track local faunal evolution across an age boundary and show that the basic features of the local insular faunas can be conserved across a few millions years at the least.

Nevertheless, isolation of the different insular faunas was not complete during the early Campanian. Minor-to-moderate levels of faunal interchange can be detected, as shown, for example, by the appearance of the foxemydin turtles (probable Austroalpine immigrants) in southwestern Europe by the early Campanian. These exchanges, however, were occasional and most probably the results of chance dispersal, since they involved only isolated faunal elements and not entire faunal modules. There is very little order or consistency to these exchanges. For example, although the dortokids and foxemydines are both groups adapted to freshwater habitats (Pérez-García et al. 2012b; Rabi et al. 2013a), foxemydines are represented by the same genus (*Foxemys*) in Hungary and southern France during the Santonian to early Campanian, while dortokids from the two areas appear to belong to distinct, eastern and western phylogenetic lines (Rabi et al. 2013a).

The ‘mid’-Campanian to early Maastrichtian represents a distinct stage in the evolution of the European continental vertebrates. During this time, faunal evolution occurred along the same general lines as those already seen in the Santonian and early Campanian: fairly distinctive local faunas undergoing *in situ* change over time, overprinted by immigrations from Gondwana and, to a far lesser degree, North America. Batrachosauroidid urodeles are the only definitive North American immigrant group that appears in the late Campanian in southern France, and it is possible that some basal alligatoroids of Ibero-Armorica also had North American affinities.

Gondwanan immigration was much more extensive during this time, with southern migrants including such groups as characiform and mawsoniid fishes, bothremydine turtles, and derived majungasaurine abelisaurids, along with perhaps some derived titanosaurs and madtsoiid snakes.

The exact timing and succession of the ‘mid’-Campanian to early Maastrichtian immigration events is unclear. Many of the aforementioned taxa make their first appearance during the late Campanian, but it is conceivable that they arrived in Europe slightly earlier and are missing from the fossil record due to sampling and/or paleoecological biases. It is clear, however, that the vast majority of these arrivals can be constrained as occurring prior to the late Campanian, except perhaps for some of the fishes (characiforms and coelacanths) that are first reported from Maastrichtian units (Cavin et al. 2005; Otero et al. 2008). Similar to what happened during the Santonian, all Campanian immigration events targeted southwestern Europe (the Ibero-Armorican landmass), with most migrant groups remaining restricted to these areas for the remainder of the Cretaceous. Two major sea-level drops during the Campanian, one in the mid-Campanian and another close to the Campanian/Maastrichtian boundary, may have been favorable moments for faunal migrations. The mid-Campanian drop in sea level is particularly well suited to explain the arrival of various North American and Gondwanan taxa in southwestern Europe.

Towards the end of the ‘mid’ Campanian–early Maastrichtian evolutionary stage, faunal connections with Asia appear to have been renewed. Derived velociraptorine dromaeosaurids (*Balaur*), and potentially derived alvarezsaurids appear in the eastern Tethyan areas (Transylvanian landmass) by the early Maastrichtian. Their appearance may be related to regional sea-level drops in the Russian Platform during the Maastrichtian and the rise of the Pontide orogenic and volcanic chains along the northern margin of the Tethys (Baraboshkin et al. 2003; Nikishin et al. 2011). Dispersal along this southern, actively tectonic orogenic segment, connected to the Carpathian areas in the west through the Balkan Orogen would explain why Asian immigrants were apparently restricted to the eastern European Tethyan landmasses during this time.

Faunal endemism and provinciality continue to characterize the late Campanian–early Maastrichtian faunas of Europe. It appears that despite large-scale compositional similarities (the shared presence of the iconic rhabdodontids, struthiosaurines, and some other clades), each major landmass featured a partly endemic assemblage. Regional endemism was especially marked between the southwestern cratonic and southeastern Tethyan areas of Europe (see also Weishampel et al. 2010). Not a single species has been found in both Transylvania and Ibero-Armorica during this time. The individual character of these two regional assemblages is also indicated by many exclusively distributed taxa: ‘kallokibotionins’, borioteiioids, hadrosauroids, and kogaionids in Transylvania vs. bothremydids, solemydids, varanoids, alligatoroids, abelisauroids, and zhelestids in Ibero-Armorica. Provinciality is also supported by the fact that even widespread taxa such as dortokids and rhabdodontids are represented by species belonging to clearly distinct eastern and western phylogenetic lineages in the two landmasses (e.g., Ősi et al. 2012a; Rabi et al. 2013a). Nevertheless, a certain degree of faunal continuity can be documented between the Santonian–early Campanian Austroalpine and the latest Campanian–early Maastrichtian Transylvanian faunas of the eastern European Tethyan archipelago, as they uniquely share ‘kallokibotionins’, borioteiioids, and representatives of the eastern groups of dortokids and rhabdodontids. While the Austroalpine landmass became completely submerged by the late Campanian, other areas of the Tethyan archipelago of Europe witness the emergence of entirely novel faunas, as derived non-hadrosaurid hadrosauroids appear to dominate on the Adriatic-Dinaric Carbonate Platform (although this dominance may represent a consequence of paleoecological-paleoenvironmental bias).

The early–late Maastrichtian boundary is marked by an important faunal turnover in Late Cretaceous European faunas. This was first discussed by Le Loeuff et al. (1994) and has often been described as a major event in which hadrosaurids replaced titanosaurs as the major herbivores of the continental faunas. According to this scenario, global environmental changes related to the important ‘mid’-Maastrichtian sea-level drop and subsequent floral modifications allowed (or drove) the rise of hadrosaurids at the expense of titanosaurs. This scenario, as originally proposed, must be emended based on the currently available fossil record: although titanosaurs do appear to have undergone a demise by the late Maastrichtian in southern France, they were present and even relatively diverse in the south-Pyrenean assemblages up to the end of the Maastrichtian (e.g., Vila et al. 2012, 2013a), and continued to exist besides hadrosauroids up to the late Maastrichtian in Transylvania (e.g., Vremir 2010; Csiki and Vremir 2011). A more nuanced scenario for this turnover event holds that titanosaur-nodosaurid-rhabdodontid faunas were replaced with hadrosaur-titanosaur faunas (e.g., Buffetaut et al. 1997b; Galobart et al. 2012; Vila et al. 2013a). This scenario better describes the succession of faunal assemblages on the Ibero-Armorican landmass, but does not apply to Transylvania, where rhabdodontids (*Zalmoxes*) and nodosaurids apparently lived alongside hadrosauroids and titanosaurs into the late Maastrichtian, suggesting there was no major turnover between the early and late Maastrichtian. It is clear, therefore, that faunal evolution on the different European landmasses was still shaped by local factors and conditions during the very end of the Cretaceous.

Although some of the scenarios may not fully explain faunal changes during the Maastrichtian, there clearly was an important faunal turnover between the early-late Maastrichtian, at least in some parts of Europe. This is best illustrated by herbivorous dinosaurs of the Ibero-Armorican landmass, where body fossil and footprint evidence clearly document the arrival and rise to dominance of hadrosauroids in local assemblages north and south of the Pyrenees (e.g., Vila et al. 2013a). This faunal change coincides with a substantial regional sea-level drop in the Atlantic (Miller et al. 2003), an extensive regression in the Russian Platform-Turgai Strait areas (Baraboshkin et al. 2003; Nikishin et al. 2011), and emersion of several Tethyan carbonate platforms (e.g., Otoničar 2007), events that also enabled the preservation of a much more extensive continental fossil record during this time by increasing the amount of emergent land (see also Figs 1, 3). Accordingly, suitable but probably discontinuous continental connections became available between cratonic Europe and both North America (the De Geer Route of Brikiatis 2014) as well as Asia (across the Turgai Strait) from the late Maastrichtian onwards, allowing dispersal between the different landmasses.

Based on the available evidence, two major faunal waves reached Europe during the late Maastrichtian, both originating on Laurasian landmasses. New North American arrivals included crocodyloids, lambeosaurin hadrosaurs, and ‘peradectid’ metatherians, whereas tsintaosaurin and aralosaurin lambeosaurines were introduced from Asia. All of these newcomers targeted the western, cratonic areas of Europe, suggesting that the available dispersal routes of the time circumvented the newly emerging orogenic chains of the southern, Tethyan regions. Remarkably, faunal changes appear to have ceased with Gondwana during the late Maastrichtian, because no southern immigrant can be identified with certainty. Substantial trans-Tethyan faunal contacts are hypothesized to have resumed around the Cretaceous-Paleogene boundary, but these involved the migration of European taxa to Africa, the reverse of the Late Cretaceous pattern (Gheerbrant and Rage 2006).

The final stage of the Late Cretaceous faunal evolution in Europe is represented by the end-Cretaceous extinction event (covered in a separate section, below). It is important to underline here that despite earlier claims to the contrary (e.g., Galbrun 1997), the last Cretaceous vertebrate remains in Europe are closely associated with the Cretaceous/Paleogene boundary (e.g., López-Martínez 2001; López-Martínez et al. 2001) and that, despite the ‘mid’-Maastrichtian faunal turnover, apparently there was no major diversity decline in the European continental vertebrate assemblages of Europe towards this boundary (e.g., Le Loeuff 2012; Vila et al. 2012, 2013a).

### Late Cretaceous island life

Islands have long been acknowledged as fundamental to our understanding of how evolution shapes the living world. These are often described as “natural laboratories of evolution”, allowing tightly-controlled study of complex interplaying sets of physical and biotic factors that control the processes of evolution. Since the work of Wallace and Darwin in the nineteenth century (Darwin 1859; Wallace 1892) islands have remained a prominent research topic in evolutionary, ecological and biogeographical studies (e.g., Whittaker and Fernández-Palacios 2007). Using islands, biologists have been able to study topics such as taxon originations, adaptive radiations, eco-morphological diversification, niche shifts, and ecosystem structuring. Their importance was highlighted most famously by MacArthur and Wilson (1967) in their seminal work on island biogeography and by the establishment of the concept of the “island rule” (Foster 1964).

Much of the early work on island evolution focused on the present-day world, but islands of the past (insular paleofaunas and paleoenvironments) also provide critical information. In particular, recent research on Cenozoic island faunas has already provided an impressive amount of data on this topic, and has contributed significantly to a more profound understanding of the basic patterns, trends and processes that control island biogeography (e.g., De Vos et al. 2007; Van der Geer et al. 2010). However, exploration of older, pre-Cenozoic insular ecosystems is still at a very preliminary stage, despite the fact that documentation of the first examples of Mesozoic insular ecosystems and their particularities dates as far back in time than that of the Cenozoic ones.

Incidentally, the “birthplace” of Mesozoic island paleobiogeography studies is the Maastrichtian Transylvanian landmass. While studying the local vertebrate assemblages of the Hațeg Basin, Nopcsa (1915, 1923a, 1934) identified several distinctive features of these faunas. These included the generally small size of taxa, low taxonomic diversity, markedly endemic composition, and the presence of species that were, in his opinion, relictual, reminiscent of older and more primitive evolutionary stages in their respective lineages. Nopcsa, an early embracer of fig tectonics (see Weishampel and Reif 1984), was able to comprehend the idea that Mesozoic Europe had a very different paleogeography than modern Europe. Thus, in order to explain their paleobiological peculiarities, he hypothesized that the Maastrichtian Transylvanian vertebrates had lived on an island. The Hațeg assemblage was thus the first identified example of a Mesozoic island ecosystem. Unfortunately, but perhaps not unexpectedly, given the unorthodox nature of Nopcsa’s interpretations, his idea of a Mesozoic island fauna in Romania was either subsequently overlooked or was cited as an exotic and unique example with no counterparts. The ‘Hațeg Island’ was not viewed as part of a larger pattern.

Nopcsa first suggested that the Transylvanian faunas lived on an island soon after the first celebrated studies of the unusual Plio-Pleistocene mammal faunas of the Mediterranean islands (e.g., Forsyth Major 1902; Bate 1903, 1906). Nopcsa (1923a) drew parallels in support of the insular nature of the Hațeg fauna from the dwarf elephant and hippopotamus-bearing faunas of Crete and Sicily, pointing out several striking examples of parallel evolution between such taxonomically and ecologically different groups (proboscidean, hippopotamid and bovid mammals vs. ornithopod, sauropod and ankylosaurian dinosaurs). Despite his intuitive and insightful “taxon-free” (see Damuth et al. 1992) characterization and comparison of these insular paleofaunas, Nopcsa’s work went largely unappreciated. Whereas Cenozoic island paleobiogeography continued to develop into a flourishing field of scientific enquiry (see review in Van der Geer et al. 2010), similar work on Mesozoic islands was lacking. Only recently has there been renewed interest in the recognition and study of Mesozoic island ecosystems (e.g., Weishampel et al. 1991; Dalla Vecchia 2001, 2003, 2008; Stilwell et al. 2005; Benton et al. 2006, 2010; Krause et al. 2006; Sander et al. 2006; Chatterjee and Scotese 2010; Buffetaut et al. 2011a; Van der Berg et al. 2012; Brusatte et al. 2013a).

Because Europe became a vast island archipelago during the Late Cretaceous, it is a model area for the study of Mesozoic island life and island paleobiogeography. Here, phenomena, processes, patterns and trends identified in extant (e.g., MacArthur and Wilson 1967; Whittaker and Fernández-Palacios 2007) or Cenozoic (e.g., Van der Geer et al. 2010) insular settings can be corroborated in significantly older ecosystems, which had a radically different taxonomic composition. The occurrence of insular characteristics in the European archipelago was discussed extensively by Nopcsa (1923a, 1934) in the early days of island paleobiogeography, and they were studied more recently in the case of the Transylvanian ecosystems (e.g., Weishampel et al. 1991, 1993, 2003, 2010; Csiki and Grigorescu 2007; Benton et al. 2010; Brusatte et al. 2013a). They have also been suggested for several other European Cretaceous landmasses (e.g., Vullo et al. 2007; Dalla Vecchia 2009; see further details below). In the following section, we briefly synthesize the current data on the evolution, ecology, and assembly of these peculiar European faunas, and discuss the common components of Cretaceous ‘island life’ in Europe.

Several distinct characteristics of Late Cretaceous European island life can be identified based on comparison of the European record to extant and earlier, Cenozoic island ecosystems. These can be grouped into two main categories: assemblage-level features (large-scale characteristics of faunal composition that are influenced by insularity) and taxon-level features (modifications affecting individual taxa due to their island habitat). Features of the first category include broad-scale compositional aspects of local assemblages, such as low overall local diversity (alpha diversity), high degrees of endemism and marked provinciality (high between-site beta diversity, but only moderate total-among-site gamma diversity), and presence of a large number of relictual taxa. Meanwhile, features of the second category include body size variations, adaptive morphological changes, and life history and metabolic shifts relative to closely related mainland (non-insular) taxa.

#### Insularity-related features of the European Late Cretaceous vertebrate assemblages

Low overall diversity was one of the main features of the latest Cretaceous Transylvanian assemblages that Nopcsa (1915, 1923a) identified as being tied to their insular island setting. He noted that only about 10 megafaunal components—species of turtles, crocodyliforms, dinosaurs, pterosaurs—made up the Hațeg fauna, and most of these taxa were present recurrently across the different areas of Europe. Although Nopcsa did take a position of extreme taxonomic lumping in advancing this idea, low generic diversity of the European Late Cretaceous vertebrate assemblages was still the dominant viewpoint accepted by later reviewers such as Lapparent (1947) and Weishampel (1990). New discoveries and taxonomic reinterpretations have shown, however, that diversity was much higher than previously acknowledged (compare the faunal lists in Weishampel [1990] and Weishampel et al. [2004], respectively), and that much of this diversity remained ‘hidden’ for a long time, being represented by small-sized taxa (i.e., amphibians, small reptiles, mammals).

However, taxic diversity of the different European Late Cretaceous assemblages remains relatively low despite all the new research and discoveries. The local diversity (considered as simple, raw taxic diversity: the number of taxa represented) of the richest latest Cretaceous faunal assemblages from Europe (Iharkút for the Santonian, Ősi et al. 2012b; Transylvania for the Maastrichtian, Weishampel et al. 2010; this review) can be compared with that of some of the most important more or less contemporaneous vertebrate assemblages from Asia or North America (Djadochta and Barun Goyot formations of Mongolia, and the Kaiparowits, Dinosaur Park, Judith River, Horseshoe Canyon or Scollard formations of western North America; Eberth 1997a,b; Benton et al. 2000; Currie and Koppelhus 2005; Titus and Loewen 2013). Not only are the Asia and North American faunas richer taxonomically at the species or genus level than those of Europe, but they also comprise a larger number of major clades. As discussed in this review, European faunas generally lack a large number of (sometimes remarkably species-rich) clades such as chondrichthyans, derived cryptodirans, most derived lizard clades other than borioteiioideans, tyrannosauroids, neoceratopsians, pachycephalosaurs, ankylosaurids, metatherians and non-zhelestid eutherians.

Low local diversity is often considered an important feature of insular assemblages due to both the widely recognized species-area effect (e.g., MacArthur and Wilson 1967; Brown 1995; Lomolino 2000) and the difficulties inherent to the colonization process of islands or other insular settings (inaccessibility, limited dispersive abilities of the different groups of organisms). Moreover, taxic diversity on islands can be further influenced by random factors that may have a more profound effect in shaping diversity than they do on larger landmasses (e.g., Lomolino and Weiser 2001). Whether the relatively low taxic diversity documented on the Late Cretaceous landmasses of Europe represents a genuine signal or is an artefact of sampling represents an important topic for future research. At this point in time, however, the European assemblages seem to be similar to the better-studied extant and earlier Cenozoic island assemblages in exhibiting low taxic diversity.

Although local diversity on the individual Late Cretaceous European landmasses was not high, overall vertebrate biodiversity of the European bioprovince was apparently quite substantial (Fig. 12). This is due to the high degree of endemicity of the local faunas, leading to a pronounced provinciality across Europe (high beta diversity). When these low-diversity insular faunas are summed together, they result in moderately high gamma diversity across Europe. The vast amount of data concerning local endemism and European provinciality has been summarized in this review, but one prime example is worth expanding on here to illustrate this point. Among Cretaceous continental vertebrates, dinosaurs are often considered animals with high dispersal potential due to their relatively large size and locomotor abilities. Many Late Cretaceous dinosaurs from North America, such as *Tyrannosaurus*, *Triceratops*, and *Edmontosaurus*, had wide ranges that encompassed hundreds or thousands of kilometers (documented by, e.g., Lehman 1987, 1997, 2001; Gates et al. 2010; Sampson et al. 2010; Titus and Loewen 2013), and migratory behavior has been hypothesized for many dinosaur groups (e.g., Fricke et al. 2011). In Late Cretaceous Europe, however, most known dinosaur genera and all species appear restricted to particular landmasses (islands). Therefore, the total diversity of Late Cretaceous European dinosaurs is high. Such a marked difference in degree of dinosaur provinciality between western North America and Europe can be accounted for by the nature of barriers promoting the provinciality; whereas latitudinal, climatic, orographic or ecological barriers have been invoked to explain the relatively subtle provinciality seen in North America, the more extreme provinciality in Europe was due to significantly less permeable barriers, reflecting the prominently archipelago-like paleogeography of the region.

The archipelago paleogeography of Late Cretaceous Europe also explains another outstanding feature of its faunas: the large number of relictual taxa. These are holdovers of more archaic evolutionary lineages that originated long prior to the Late Cretaceous, and which are more basal than members of the same major clades that were living contemporaneously in Asia and North America. Many of these are known from Transylvania (Weishampel et al. 2010) as well as from other European islands. They include meiolaniform turtles (which are otherwise distributed mainly in the Lower Cretaceous to Pleistocene of Gondwanan landmasses; Rabi et al. 2013b; Sterli et al. in press), basal borioteiioids and atoposaurids (whose European members are the youngest records of these groups; Martin et al. 2010, 2014; Nydam 2013), zhelestid mammals (which are closely related to basal Cenomanian taxa from Central Asia; Archibald and Averianov 2012), kogaionid multituberculates (that represent a basal branch of the derived cimolodontan clade, which originated in the late Early Cretaceous; Csiki and Grigorescu 2006), struthiosaurine ankylosaurs (which stemmed from an early-diverging line of nodosaurids that split during the late Early Cretaceous: Ősi 2005; Kirkland et al. 2013), basal non-hadrosaurid hadrosauroids (remnants of lineages that diverged before the split between saurolophines and lambeosaurines in the pre-Santonian; Sues and Averianov 2009; Prieto-Márquez 2010a), and rhabdodontids (which must have diverged from other iguanodontians by at least the Late Jurassic; Weishampel et al. 2003; McDonald 2012; Ősi et al. 2012a).

The archaic, relict nature of the Late Cretaceous European faunas is due not only to the survival of certain basal (‘primitive’) taxa, but to the dominance of of these species. These ‘living fossils’ were not simply exotic minutiae of the European asssemblages, but instead made up the core of the local faunas. Rhabdodontids are a prime example, as this clade must have originated tens of millions of years prior to the latest Cretaceous but then flourished in Late Cretaceous Europe, comprising the primary large-bodied herbivores on landmasses such as Transylvania. The prospering of so many relict taxa—belonging to several different lineages with markedly dissimilar evolutionary histories, ecological requirements, and lifestyles—suggests that these ancient lineages were sheltered in refugia all across Late Cretaceous Europe, a pattern that is again concordant with an insular, archipelago-type setting.

#### Insularity-related adaptations of the European Late Cretaceous island-dwelling taxa

Along with entire faunas, individual European Late Cretaceous taxa also exhibit peculiarities related to their insular habitat. The most widely cited, and apparently widespread, of these are changes in body size compared to mainland taxa and close relatives. Body size changes have been widely observed in insular island habitats, and were considered so ubiquitous that they were claimed to represent the effects of a generalized evolutionary law – the ‘island rule’ of Foster (1964). This ‘rule’ holds that small-sized taxa tend to become larger on islands, whereas large-sized taxa tend to become smaller, often evolving into ‘dwarves’ (see also Lomolino 2005). Although the universality of this ‘rule’ has been challenged (e.g., Meiri et al. 2004, 2008, 2011; Itescu et al. 2014), there is still evidence for body size shifts (especially dwarfing) in insular habitats for at least certain taxa, both extant (e.g., Bromham and Cardillo 2007; Welch 2009; Meiri et al. 2011) and fossil (e.g., Van der Geer et al. 2010; Herridge and Lister 2012).

**Figure 13.**
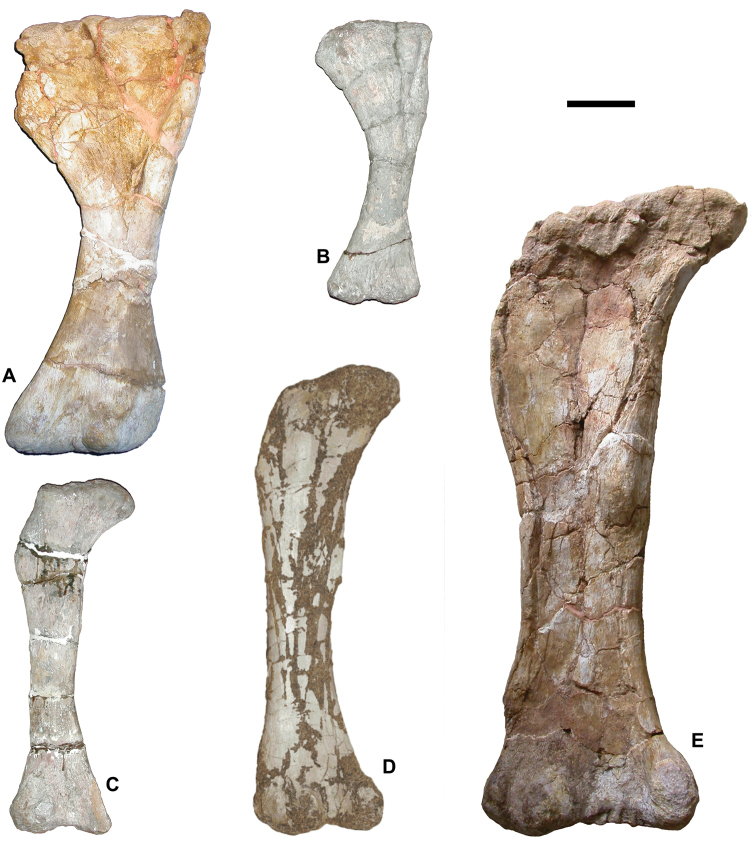
Body-size disparity in Late Cretaceous European titanosaurs, as illustrated by their appendicular elements (specimens figured at scale). **A, E** *Ampelosaurus atacis* (late Campanian–early Maastrichtian, Bellevue, Aude, southern France): **A** Left humerus (MDE C3-86), anterior view **E** Right femur (MDE C3-87; reversed), posterior view **B, C** *Magyarosaurus dacus* (early Maastrichtian, Ciula Mare, Haţeg Basin, Romania) **B** Left humerus (LPB (FGGUB) R.1047), anterior view **C** Left femur (LPB (FGGUB) R.1046), posterior view **D** *Lirainosaurus astibiai* (late Campanian–early Maastrichtian, Laño, Basque Country, northern Spain), left femur (MCNA 7468), posterior view. Scale bar equals 10 cm. Photograph **D** courtesy by Verónica Díez Díaz.

It has long been noted that the European Late Cretaceous faunas include many small-sized representatives belonging to clades that have a larger mean body size elsewhere in the world (Fig. 13). Many of these are dinosaurs. Recent descriptions of the still poorly known Cenomanian vertebrates from the Czech Republic (Fejfar et al. 2005) and France (Vullo et al. 2007) emphasized the small size of the recovered taxa, interpreted as a consequence of the insular environments these animals lived in. Dwarfism among dinosaurs has also been identified in the ‘mid’-Cretaceous eastern European Tethyan island areas (Dalla Vecchia 2003), and numerous examples of small-sized dinosaurs have come to light in the much more extensive Santonian–Maastrichtian fossil record of Europe. The most famous of these European dwarfed dinosaurs come from the Maastrichtian (and possibly uppermost Campanian) of Transylvania. Nopcsa (1915, 1923a) first noted the small size of some species, and his hypothesis of island dwarfing in these dinosaurs has been corroborated by many recent studies. The Transylvanian dwarfed dinosaurs include the hadrosauroid *Telmatosaurus* (Weishampel et al. 1993), the rhabdodontid *Zalmoxes* (Weishampel et al. 2003; but see Ősi et al. 2012a), the titanosaurian *Magyarosaurus* (Jianu and Weishampel 1999; Stein et al. 2010; Fig. 13), and the nodosaurid *Struthiosaurus* (Ősi et al. 2014a). Importantly, the reality of small adult body size was supported through osteohistological studies of all these taxa (Benton et al. 2010; Stein et al. 2010; Ősi et al. 2014a), contradicting previous assertions that observed small body size might represent a taphonomic or preservational artefact with preferential preservation of smaller juveniles rather than a real paleobiological pattern (e.g., Le Loeuff 2005b). Interestingly, the predatory dromaeosaurids (*Balaur*) from the same assemblage do not appear to have underwent significant body size reduction, a pattern that compares well with that recorded in the case of herbivores and carnivores in present-day insular habits (Brusatte et al. 2013a).

Small adult body size has also been reported in several other European dinosaurs of the Late Cretaceous. Both Austrian and Hungarian species of *Mochlodon* were described as dwarfed rhabdodontids by Ősi et al. (2012a), an assertion also supported by their osteohistology. This is also the case for the recently reported Hungarian material of *Struthiosaurus* (Ősi and Prondvai 2013), and other specimens referred to this genus from across Europe are small as well (Pereda-Suberbiola and Galton 2009). Small adult body size and osteohistological data suggest that the Ibero-Armorican titanosaur *Lirainosaurus* has reduced its body size compared to other titanosaurs (Company 2011). Body fossils demonstrate that not all Ibero-Armorican hadrosaurids were small (e.g., Fondevilla et al. 2013), but the more extensive trackway record shows that these taxa were smaller on average than those outside of Europe (Vila et al. 2013a). Many small-bodied hadrosauroids are also found in more poorly sampled faunas across Europe (including in Bulgaria, Germany, Crimea; Wellnhofer 1994; Nessov 1995), which mirrors the pattern shown by the better-preserved, definitively small-sized hadrosauroids from Transylvania.

One remarkable aspect of these suggested body-size changes concerns their speed. Probably the most impressive case is that of the late Maastrichtian lambeosaurines from the Ibero-Armorican landmass. Although most Asian tsintaosaurins and North American lambeosaurins were not gigantic, they attained often considerable body size. According to the fossil record, they would have transformed into moderate-to-small-sized taxa soon after their arrival on the Ibero-Armorican landmass, probably within 2 million years. Better quantifying the speed of these body size changes could offer interesting insights into still hidden aspects of insular adaptations during the Cretaceous. Regardless of the exact rates of change, the swiftness of the process should not neccessarily be surprising, because increased rates of morphological changes are known to occur in present-day insular settings (e.g., Millien 2006).

Besides body-size changes, insularity also affected the morphology of island-dwelling Late Cretaceous European taxa through alterations to the general body plans, in order to accommodate the new colonists to their novel habitat. A baseline expectation of possible modifications is documented in the Cenozoic fossil record of island species, including shifts to more graviportal but dynamically more stable stances in primitively cursorial taxa such as bovids and cervids (e.g., Köhler and Moya-Sola 2001; Van der Geer et al. 2006), shifts to a more slender body plan or shortening of the legs in the case of more massive animals such as proboscideans, hippopotami, and suids (e.g., Van der Geer et al. 2010), or changes to dentition (e.g., Jordana and Köhler 2011; Jordana et al. 2012).

Similar possible island-dwelling morphological adaptations have also been reported from the latest Cretaceous of Europe. These include the more cursorial stance of the struthiosaurine *Hungarosaurus* (Ősi and Makádi 2009) compared to those in other nodosaurids, the distally shortened hindlimbs showing extensive fusion between individual bones in the dromaeosaurid *Balaur* (Brusatte et al. 2013a), and the more slender, less graviportal built reported in hadrosauroids such as the Crimean ‘*Orthomerus*’ *weberae* (Nessov 1995) and the Adriatic *Tethyshadros* (Dalla Vecchia 2009a). In the case of *Tethyshadros*, a complex of modifications affecting both forelimbs and hind limbs (tightened and more elongated hind limb, closely appressed manus elements acting as support during quadrupedal walking) were interpreted by Dalla Vecchia (2009a) as adaptations to moving across rough landscape, a likely setting within a tectonically active insular environment. A similar scenario was put forth for the predatory *Balaur* by Brusatte et al. (2013a).

Finally, island life-related adaptations in Cenozoic mammals are known to affect metabolic status, life history strategies, growth rate, sense organs, and even neurological activity (e.g., Raia et al. 2003; Palombo et al. 2008; Köhler and Moya-Sola 2009). Most of these changes are difficult to identify in the fossil record, but there are some indications that these types of changes are seen in European Late Cretaceous island-dwelling taxa. One of the most striking examples concerns the distinct slowdown of the growth rate, and possibly correlated reduction in metabolic rate, in several dwarfed (*Magyarosaurus*, *Lirainosaurus*; Stein et al. 2010; Company 2011) or more normal-sized (*Ampelosaurus*; Klein et al. 2012) titanosaurs. Similar, although less pronounced, decreases in growth rate were also proposed for the ornithopods *Zalmoxes* and *Telmatosaurus* (Benton et al. 2010). Finally, a protracted cyclical growth period was identified in the case of the giant terrestrial bird *Gargantuavis* (Chinsamy et al. 2014), reminiscent of that seen in extant and subfossil insular ground-dwelling birds, which supports the idea that *Gargantuavis* was adapted to an island habitat.

### Events at the Cretaceous-Tertiary boundary in the European Archipelago

The best-dated latest Maastrichtian fossiliferous continental deposits of Europe are from the northern and southern Pyrenean areas of the Ibero-Armorican domain (see above). Furthermore, these deposits are covered locally by Paleocene continental deposits in the southern Pyrenean areas (López Martínez et al. 1998, 1999, 2001; López Martínez 2003b) and thus allow a fairly reliable assessment of the impacts of the Cretaceous/Paleogene (K-Pg) boundary events in Europe and correlations with K-Pg extinction patterns reported from other parts of the world.

#### Dinosaurs

There were changes in dinosaurian faunas that occurred during the Maastrichtian in at least part of Europe, most notably the Ibero-Armorican Domain in the southwest. Some groups of dinosaurs, such as rhabdodontids, declined and probably became extinct at the beginning of the late Maastrichtian in this area (López Martínez et al. 2001; Pereda-Suberbiola et al. 2004), although probably not in other parts of Europe such as Transylvania (e.g., Smith et al. 2002). As noted first by Le Loeuff et al. (1994), during the late Maastrichtian a vertebrate assemblage dominated by hadrosaurids largely replaced an early Maastrichtian assemblage dominated by titanosaurian sauropods. This replacement, however, did not cause a complete disappearance of titanosaurs on this landmass (Buffetaut and Le Loeuff 1997). Struthiosaurines also apparently became less common during the late Maastrichtian in southwestern Europe (e.g., Riera et al. 2009). All of these changes may have coincided with sea-level changes during a marine regression (Le Loeuff et al. 1994).

Changes were clearly afoot in the Maastrichtian dinosaur faunas of Europe, and it is thought that some of these may have been related to the ultimate extinction of non-avian dinosaurs at the end of the Cretaceous. Based on the last occurrence of *in situ* eggshells, some authors have suggested previously that non-avian dinosaurs disappeared in Europe well before the K-Pg boundary, perhaps more than two million years earlier (Colombo 1996; Galbrun 1997). In a similar vein, López Martínez (2003a) noted that at least a one-meter *gap* separated the stratigraphically youngest dinosaur fossils from the first Paleocene level, marked by an isotopic ^13^C anomaly, which may indicate a local extinction before the end of the Cretaceous. Other workers suggested that European dinosaurs were decreasing in diversity prior to the K-Pg boundary and argued for a gradual and diachronous extinction of non-avian dinosaurs across the continent (López Martínez et al. 1999, 2001; López Martínez 2003b).

The wealth of recent data from the southern Pyrenees falsifies these hypotheses and shows that dinosaurs definitely lived in at least parts of Europe during the last few hundred thousand years of the Cretaceous and that their diversity was not decreasing markedly before their extinction. Most of this information comes from the upper levels of the Tremp Formation of northern Spain, which has been extensively sampled over the past decade (Riera et al. 2009; Vila et al. 2012, 2013a). Importantly, this ever-improving end-Cretaceous record in Spain has great potential to complement the heavily North America-dominated record of dinosaur evolution during the final few million years prior to the K-Pg boundary.

The Tremp record includes the richest and stratigraphically youngest succession of dinosaur footprints in Europe, including 25 localities in the C29r magnetochron, within approximately the last 400,000 years of the Cretaceous. The uppermost unequivocal evidence of dinosaur tracks, attributable to the ornithopod ichnotaxon *Hadrosauropodus*, occurs 14 m below the K-Pg boundary (Vila et al. 2013). In the same area, the so-called Reptile Sandstone, a conspicuous 7 meter-thick level that occurs about 10 m under the base of the Danian Vallcebre limestones (Oms et al. 2007), has yielded hadrosaurid skeletal remains and isolated figs of a bothremydid turtle (Blanco et al. 2013). These finds, together with specimens of the lambeosaur *Canardia* from marine deposits of Haute-Garonne in southern France (Bilotte et al. 2010; Prieto-Márquez et al. 2013) are among the stratigraphically youngest dinosaur remains found in Europe to date. These finds definitively show that dinosaurs were living in this region of Europe during the final few hundred thousand years of the Cretaceous and most likely would have witnessed the bolide impact at the K-Pg boundary.

The Tremp succession includes numerous hadrosauroid bones and footprints very close to the boundary, which indicate that these large dinosaurs were locally thriving during the latest Cretaceous. The same is apparently true for other groups of dinosaurs. Vila et al. (2012) have reassessed the diversity of the latest Ibero-Armorican titanosaurs within a precise and clear chronostratigraphic framework. They showed that the youngest sauropod tracks occur in C29r, demonstrating that, like hadrosaurids, sauropods persisted in Europe during the final few hundred thousand years of the Cretaceous. The stratigraphically youngest skeletal records of sauropods are slightly older and include two indeterminate taxa that fall within the uppermost part of magnetochron C30n, in the latest Maastrichtian (~0.4–1.5 Ma before the K-Pg boundary).

The European non-avian theropod record is much more limited than those of hadrosaurids and sauropods but current data suggest that there was no significant decrease in theropod diversity near the very end of the Cretaceous (Ősi et al. 2010; Torices et al. in press). An apparent drop in diversity observed by Sellés et al. (2014a) on the Iberian Peninsula is probably an artifact due to inaccurate dating of some sites. New age assignments for a number of localities from southern Pyrenees support high taxonomic theropod diversity during the late Maastrichtian (Sellés and Vila, submitted). This is in agreement with global diversity and morphological disparity measures indicating no major changes in theropod biodiversity during the latest Cretaceous elsewhere (Upchurch et al. 2011; Brusatte et al. 2012, 2014).

The European record of latest Cretaceous birds is also poor. However, the current data show that both enantiornithines and ornithurines existed in the Limburg area during the late Maastrichtian (Dyke et al. 2008), and enantiornithines are also known from possibly ‘mid’- to upper Maastrichtian beds in Transylvania (Wang et al. 2011b; Dyke et al. 2012). The stratigraphically youngest record of the probably ornithurine giant bird *Gargantuavis* is early Maastrichtian in age (Buffetaut and Angst 2013) but is unclear whether this endemic Ibero-Armorican taxon persisted into the late Maastrichtian. However, it clearly appears that neither enantiornithines (Longrich et al. 2011; Feduccia 2014), nor *Gargantuavis* (Buffetaut 2002) survived the end-Cretaceous extinction event. Unfortunately, the taxonomically indeterminate status of late Maastrichtian ornithurine fossils from Europe precludes any assessment of potential survival or extinction.

In summary, the local-scale data from the Tremp Basin, together with other information about the evolution of dinosaur diversity in Europe through time (as summarized in this review), suggest that the diversity of dinosaurs did not experience any marked decline at the end of the Cretaceous in Europe. Although more precise radiometric dates would help better interpret how the very last surviving European non-avian dinosaurs evolved in concert with latest Cretaceous climate and sea-level changes as well as volcanism, it is at least clear that dinosaurs survived in Europe into the final 400,000 years of the Cretaceous. This mirrors the pattern observed in North America (e.g., Sheehan et al. 2000; Pearson et al. 2002; Brusatte et al. 2014). As far as can be ascertained, the Pyrenean fossil record is compatible with a sudden dinosaur extinction at the K-Pg boundary. This event was preceded by important faunal changes in certain parts of Europe that do not foreshadow the disappearance of non-avian dinosaurs but were instead dispersal-driven and perhaps sea-level driven faunal turnovers, and the extinction of non-avian dinosaurs does not seem to be a gradual culmination of any of these trends.

#### Other vertebrates

It is interesting to compare this pattern of sudden dinosaurian extinction with the evolutionary trends observed for other groups of Late Cretaceous continental vertebrates. These trends for most groups are less well understood than those for dinosaurs. This is due to the cumulative effects of low taxonomic resolution in case of many of these clades, less reliable dating of the fossiliferous beds available from parts of Europe other than northern Spain (including uncertainties concerning the position of the K-Pg boundary itself), and a poor fossil record close to the boundary, especially in the overlying lower Paleocene. With these caveats in mind, we summarize what is currently known about the latest Cretaceous evolutionary and extinction patterns of various continental clades, but recognize that these are liable to change with new discoveries.

Pterosaurs are known to have survived until the end of the Cretaceous in Europe. Ornithocheirid pterosaurs, the common pterosaurian clade of the ‘mid’-Cretaceous, were replaced by azhdarchids and went extinct during the middle Late Cretaceous, with a possible case of isolated late survival into the Campanian of Russia. Azhdarchids, which represent the final flourishing of pterosaurs both in Europe and other parts of the northern continents, range into the late Maastrichtian, at least in Transylvania (e.g., Vremir 2010) and Spain (Company et al. 1999; Dalla-Vecchia et al. 2013) before disappearing completely from the fossil record. The Spanish record includes specimens from magnetochron C29r (Dalla Vecchia et al. 2013), definitively showing that giant azhdarchids existed at the very end of the Cretaceous, and are therefore on the list of taxa that died out at, or close to, the K-Pg boundary.

Crocodyliform evolution across the K-Pg boundary is relatively poorly understood. Vasse and Hua (1998) were unable to identify a clear-cut extinction event at the boundary, although they noted the gradual replacement of more basal mesoeucrocodylians by eusuchians near the end of the Late Cretaceous (see also Buscalioni et al. 2003; Buscalioni and Vullo 2008; Martin and Delfino 2010). Recent reinterpretations, however, identified a definitive disruption in the composition of the European crocodyliform assemblages that roughly coincides with the K-Pg boundary. Hylaeochampsids (including *Allodaposuchus*) extend into the late Maastrichtian in Spain (Puértolas-Pascual et al. 2014) and possibly Romania (Delfino et al. 2008a; Vasile et al. 2011b), but have yet to be recorded from Paleocene or younger beds. Furthrmore, although there are basal alligatoroids in the Paleogene of Europe, these do not appear to be closely related to the European latest Cretaceous forms, and more likely represent post-Cretaceous immigrants (e.g., Narváez and Ortega 2011). These observations suggest that the most common and widespread crocodyliform taxa of latest Cretaceous Europe may have disappeared at or near the K-Pg boundary, or perhaps slightly earlier for the alligatoroids (as none has yet been found in the late Maastrichtian of Europe).

The fossil record of the latest Cretaceous squamates is rather meager, taxonomically problematical, and chronostatigraphically poorly constrained. Madtsoiids are still present in the ‘middle’ to upper Maastrichtian of Romania (Vasile et al. 2013), but completely disappear from the European fossil record after the Cretaceous (e.g., Rage 2012). The stratigraphically youngest European borioiteiioids (*Bicuspidon hatzegiensis*) and paramacellodids (*Becklesius*) are also known from the Maastrichtian of Romania (Folie and Codrea 2005; Vasile et al. 2011b), but their precise ages are uncertain and it is not clear whether they extended to the K-Pg boundary. Regardless, because both of these groups are unknown from the Paleocene of Europe, it is likely that they went extinct during the K-Pg boundary event (Rage 2012). Although they are not currently sampled, it is possible that other lizard groups such as iguanids and teiids might have survived into the Paleocene in Europe, as they appear again in the Eocene record (Rage 2012). This situation may mirror that reported in the latest Cretaceous of North America, where borioteiioids became extinct at the K-Pg boundary whereas some other lineages of lizards survived into the Paleocene (Longrich et al. 2012).

Amphibians potentially exhibit a remarkable rate of survival across the Cretaceous-Paleogene boundary in Europe. Blain et al. (2010) noted that virtually all well-known amphibian taxa (such as the robust-snouted *Albanerpeton*, as well as discoglossid and palaeobatrachid frogs) from the late Maastrichtian Blasi 2 site in northern Spain survived in Europe into the Paleogene or even later. Discoglossids and albanerpetontids are also reported from the late Maastrichtian of Romania (Vasile et al. 2011b). Another group, batrachosauroidids, are known from both the Campanian and Paleocene of Europe and thus in all appearences extended across the boundary (Rage 2012). It is conceivable, however, that some of these Paleocene taxa might have been reintroduced from North America or elsewhere after the K-Pg boundary, and do not represent local lineages surviving the extinction. This was not the case for palaeobatrachids, however, which represent an endemic European group.

European turtles exhibit an unusual bipartite pattern across the K-Pg boundary. More terrestrially adapted taxa such as *Kallokibotion* and solemydids disappear at, or slightly before, the K-Pg boundary. *Kallokibotion* is known from the upper Maastrichtian of the Transylvanian Basin in Romania (Codrea and Vremir 1997), and solemydids occur at least up to the mid-Maastrichtian (e.g., Lapparent de Broin and Murelaga 1999; Lapparent de Broin 2001; Joyce et al. 2011). However, neither group is known from deposits younger than Cretaceous, and after the K-Pg event they are replaced by modern terrestrial turtle clades. On the other hand, the more aquatic bothremydids and dortokids exhibit a different pattern. Bothremydids occur at last in the late Maastrichtian in northern Spain (e.g., Marmi et al. 2012b), and probably disappeared in Europe after the Cretaceous, when they were replaced by the closely related aquatic taphrosphyrines beginning in the Paleocene (e.g., Gaffney et al. 2006). The dortokids, however, extend together with *Kallokibotion* into the late Maastrichtian of Transylvania, Romania (Vremir 2010), survived the K-Pg boundary event, and are represented in the upper Paleocene–lower Eocene of Transylvania by taxa apparently closely related to the Late Cretaceous forms (Lapparent de Broin et al. 2004; Rabi et al. 2013a; Vremir 2013). As such, dortokids are one of the few cases where survival of a major terrestrial vertebrate subclade across the K-Pg boundary can be definitively documented by fossils.

Mammals also have a bipartite pattern of extinction and survival across the K-Pg boundary in Europe. The stratigraphically youngest zhelestids have been reported from the Maastrichtian of northern Spain (Pol et al. 1992) and southern France (Tabuce et al. 2004). Tabuce et al. (2013) demonstrated that the French forms are from magnetochrons 31r-31n, thus dating to the late (but not latest) Maastrichtian. Kogaionid multituberculates are reported from ‘middle’ to upper Maastrichtian deposits of the Haţeg Basin (Codrea et al. 2002; Smith et al. 2002; Vasile et al. 2011b). Therefore, according to the currently available data, it seems that representatives of both clades were present until very close to the K-Pg boundary. However, their fates across the boundary were strikingly different: whereas zhelestids went extinct near the K-Pg boundary and are not known from Cenozoic beds, kogaionids show a moderate taxonomic and geographic range extension during the same time interval, and they were present in the Paleocene of Spain, France, Belgium and Romania (e.g., Vianey-Liaud 1986; Gheerbrant et al. 1999; Peláez-Campomanes et al. 2000). It appears that kogaionids underwent a burst of dispersal in western Europe sometimes around the boundary, probably aided by marine regressions and emergence of land areas across central and western Europe at the end of the Maastrichtian and after the K-Pg boundary (Csiki and Grigorescu 2002, 2006). Their dispersal was soon followed by their gradual demise and progressive replacement by more derived neoplagiaulacid immigrants, culminating in their disappearance by the end of the Paleocene.

#### Patterns of extinction and survival near the Cretaceous–Paleogene boundary

This survey of the latest Cretaceous record of non-dinosaurian continental vertebrates from Europe reveals remarkable similarities with the patterns of dinosaur evolution during the same time interval. It appears that European faunas were profoundly remodelled around the K-Pg boundary. The faunal changes affected not only dinosaurs, but also different groups of turtles, lizards, snakes, crocodyliforms, pterosaurs and mammals. Furthermore, extinctions appear to have been rather sudden and clustered temporally near the K-Pg boundary.

Taken together, the available data clearly suggest that a catastrophic extinction event affected the latest Cretaceous continental vertebrate assemblages of the European archipelago. This is similar to what is seen in the much more extensive North American fossil record (e.g., Sheehan and Fastovsky 1992; Sheehan et al. 2000; Pearson et al. 2002; Longrich et al. 2011, 2012; Brusatte et al. 2014; Wilson 2014). At face value, this demonstrates that both large continental landmasses and more fragmented archipelagos were similarly decimated at the end of the Cretaceous, lending further credence to the universality of a sudden mass extinction at this time.

Unfortunately, the European latest Cretaceous fossil record is still plagued by rather uneven sampling and poor chronostratigraphic constraints. This stands in contrast to the rich, well-sampled, and stratigraphically well-constrained record of North America, which has allowed scientists to understand high-resolution evolutionary trends in vertebrate evolution and extinction (e.g., Hartman et al. 2002; Wilson et al. 2014a). Many latest Cretaceous vertebrate taxa from Europe are still known from single occurrences, their exact chronostratigraphic position is poorly constrained, and/or the records come from different isolated landmasses (islands) and thus possibly experienced markedly different ecological constrains and evolutionary histories. Accordingly, no quantitative assessment of European faunal trends prior to, across, and after the K-Pg boundary is yet possible, especially not at low taxonomic levels. However, a few comparisons with the North American record will be attempted here, in order to point out potential similarities and differences. Since extremely few genera (let alone species) described from the latest Cretaceous are known to have crossed the K-Pg boundary into the Paleocene, discussions will be focused mainly at higher taxonomic levels.

The most obvious similarity Europe and North America is the extinction of all the latest Cretaceous non-avian dinosaurs, along with various bird clades. According to our current understanding, not a single representative of a latest Cretaceous bird clade in Europe crossed the K-Pg boundary; some ornithurines might represent an exception to this pattern, but the available data is simply insufficient to either prove or reject such a hypothesis. This pattern is extremely similar to the case of North America, where all archaic, non-neornithine bird lineages sampled in the latest Cretaceous disappeared at or near the K-Pg boundary (Longrich et al. 2011). Interestingly, large ground birds of the latest Cretaceous (*Gargantuavis*) went extinct completely in Europe, only to be replaced by the similarly large-sized gastornithines starting in the Paleocene. Whereas *Gargantuavis* was a rare component of the latest Cretaceous continental assemblages, the gigantic ground birds of the Paleocene fluorished in the post-extinction recovery ecosystems, filling in the niche of the top herbivores in a pattern reminiscent of that on prehistoric large islands (Madagascar, New Zealand; Angst et al. 2014).

Mammals also experienced major extinction around the K-Pg boundary in both Europe and North America. Overall diversity of mammals in Europe was very low at both higher and lower taxonomic levels even during the latest Cretaceous, with only 3 families represented, out of which one (Zhelestidae) went extinct near the end of the Cretaceous. Meanwhile, the multituberculate kogaionids survived the boundary events, and it is possible that an individual genus (*Hainina*) may have crossed the boundary (Csiki and Grigorescu 2000). Metatherians may or may not have extended across the boundary in Europe, depending on the phylogenetic relationships of *Maastrichtidelphys*. The high survival rate of the European multituberculates contrasts with the patterns observed in North America and Asia. In North America, multituberculates apparently were more profoundly affected by the K-Pg extinction event than eutherians (e.g., Wilson 2014). In Central Asia, the abundant endemic djadochtatherian multituberculates of the latest Cretaceous disappear in the latest Cretaceous and were replaced during the Paleogene by taeniolabidoids of probably North American origin (Kielan-Jaworowska and Hurum 2001). It is not clear whether this replacement began during the latest Cretaceous or at the boundary, because reasonably complete fossils of late Maastrichtian Asian multituberculates have yet to be reported. Regardless, there was a clear turnover between latest Cretaceous and Paleogene multituberculates in Asia, unlike the case in Europe.

Turtle survival patterns in Europe also differ strikingly from those reported in North America, where turtle assemblages were little affected by the K-Pg extinction event and where most Cretaceous lineages, even many individual genera, extended from the Cretaceous into the Paleogene (e.g., Hutchinson and Archibald 1986; Jasinski et al. 2011; Holroyd et al. 2014). In Europe, by contrast, out of the four major turtle lineages present during the late Maastrichtian, three (bothremydines, meiolaniform ‘kallokibotionins’, solemydids) disappeared. Furthermore, although dortokids survived the extinction, they apparently went extinct locally in western Europe (e.g., Pérez-García et al. 2014), and are reported only from eastern Europe during the Paleocene (Romania; Lapparent de Broin et al. 2004). Altogether, the relative diversity loss of European turtles around the K-Pg boundary was significantly higher than in North America, as summarized by Pérez-García (2012b), contra Laurent et al. (2002b).

Latest Cretaceous European crocodyliforms were also affected by the end-Cretaceous events, despite earlier suggestions to the contrary (e.g., Vasse and Hua 1998). Earlier accounts held that crocodyliforms were relatively unaffected by the K-Pg extinction event in both Europe and North America (e.g., Sullivan 1987; Buffetaut 1990), but this assessment have been changed with new discoveries and taxonomic reinterpretations. It is now understood that the impressive Late Cretaceous crocodyliform diversity dropped significantly in all landmasses, with the disappearance of many notosuchian lineages in Gondwana (e.g., Kellner et al. 2014) and of neosuchians in North America (e.g., Lucas 1992; see also Brochu 1997) and Europe, where the late-surviving atoposaurids and hylaeochampsids as well as the basal alligatoroids all vanished. Europe, therefore, exhibits generally similar patterns of crocodyliform loss near or at the K-Pg boundary as North America and other landmasses.

Latest Cretaceous European squamates also demonstrate similar evolutionary trends to those in North America. The disappearance of the borioteiioids parallels their extinction in North America and Asia (Longrich et al. 2012). Anguids and iguanids seem to have extended across the K-Pg boundary in Europe, just as in North America, where these clades exhibit some of the highest rates of cross-boundary survival of any squamates (Longrich et al. 2012). Amphisbaenians seem to first appear in Europe during the early Paleocene (Folie et al. 2013); earlier reports of potential Cretaceous forms (Rage 1999) have since been dismissed (Augé 2012). This is also the case in North America, where amphisbaenians appear after the boundary and, together with anguids and iguanids, dominate the early–middle Paleocene squamate assemblages.

Unlike all previously discussed groups, amphibians appear to have crossed the K-Pg boundary in Europe with few losses. All of the major higher-level taxa known in the latest Cretaceous survived into the Paleogene. Discoglossids, palaeobatrachids, batrachosauroidids, and possibly salamandrids are reported from Paleocene deposits of Europe (Rage 2012) and must have survived the extinction. Albanerpetontids are present in the Cretaceous and reappear in the European fossil record in the Oligocene. As the Cenozoic taxa are members of the ‘robust-snouted’ clade also known from the latest Cretaceous, they were most likely local survivors that have remained unsampled in the Paleocene–Eocene (Venczel and Gardner 2005; Gardner and Böhme 2008). This is very similar to the situation in North America, where albanerpetontids and most salamandrids were little affected by the end-Cretaceous extinction event (e.g., Sheehan and Fastovsky 1992; Wilson et al. 2014b).

Ecological selectivity of the European extinctions around the K-Pg boundary is also similar to that seen in North America, where Sheehan and Fastovsky (1992) noted the preferential survival of aquatic taxa compared to the terrestrial ones, perhaps because the aquatic taxa were part of detritus-based rather than plant-based food chains (Sheehan and Hansen 1986). Among turtles, the largely terrestrial solemydids and meiolaniforms went extinct around the K-Pg boundary, whereas the aquatic turtles fared better. This pattern is reminiscent the turtle extinction patterns in North America (Holroyd et al. 2014). With the exception of crocodyliforms, all other European groups affected by high extinction rates (squamates, mammals, dinosaurs) are exclusively terrestrial, whereas among crocodyliforms both terrestrial (atoposaurid) and aquatic (hylaeochampsid) taxa were eliminated. Conversely, amphibians – including predominantly aquatic (batrachosauroidids, discoglossids, palaeobatrachids) or secretive (albanerpetontids; Gardner 2001; Gardner and Böhme 2008; Maddin et al. 2013) forms – show high rates of survival, again similar to the pattern described for North America (e.g., Sheehan and Fastovsky 1992; Wilson et al. 2014b).

Among mammals, the complete demise of the insectivorous zhelestid eutherians and survival of the (at least partly) larger-sized and probably omnivorous (e.g., Wilson 2013) kogaionid multituberculates is somewhat counterintuitive because it is often thought that environmental disturbances around the K-Pg boundary favoured the survival of taxa that were secretive (e.g., Robertson et al. 2004) and/or dependent on secondary or tertiary productivity (e.g., Sheehan and Hansen 1986; Sheehan and Fastovsky 1992). It also departs from the pattern of the North American mammal turnover around the K-Pg boundary, where the mainly insectivorous eutherians show lower extinction rates than the dominantly omnivorous multituberculates, and where smaller taxa with more generalized diets seem to have preferentially survived (Wilson 2013, 2014).

In conclusion, it appears that many patterns of animal evolution and extinction around the K-Pg boundary are similar in Europe and North America, despite the relatively poorer quality of the European record and the fact that it currently allows only coarse assessments. These similarities include relatively high extinction rates during the late Maastrichtian, clustered near or at the K-Pg boundary. Groups of organisms strongly affected by the mass extinction in North America (and occasionally in other landmasses with a less well documented K-Pg boundary fossil record), such as non-avian dinosaurs, archaic birds, crocodyliforms, squamates, and mammals were also heavily affected in Europe. Furthermore, the ecological selectivity of the extinction events is largely similar on both landmasses, as the extinction affected terrestrial taxa more severely than more aquatic taxa. There are, however, certain differences worth noting between the extinction patterns seen in the two areas, especially in the case or turtles and mammals, and identifying the underlying causes may contribute significantly to a more profound understanding of the K-Pg extinction event. Overall, however, the European fossil record appears consistent with the scenario of sudden extinction around the K-Pg boundary, followed by a profound restructuring of continental ecosystems during the Paleocene, as in North America and elsewhere.
